# Rapid Disruption of Dishevelled Activity Uncovers an Intercellular Role in Maintenance of Prickle in Core Planar Polarity Protein Complexes

**DOI:** 10.1016/j.celrep.2018.10.039

**Published:** 2018-11-06

**Authors:** Margarida Ressurreição, Samantha Warrington, David Strutt

**Affiliations:** 1 Department of Biomedical Science, University of Sheffield, Western Bank, Sheffield, S10 2TN, UK

**Keywords:** planar polarity, planar cell polarity (PCP), signaling, disheveled, prickle, Drosophila, genetic tools

## Abstract

Planar polarity, the coordinated polarization of cells in the plane of a tissue, is important for normal tissue development and function. Proteins of the core planar polarity pathway become asymmetrically localized at the junctions between cells to form intercellular complexes that coordinate planar polarity between cell neighbors. Here, we combine tools to rapidly disrupt the activity of the core planar polarity protein Dishevelled, with quantitative measurements of protein dynamics and levels, and mosaic analysis, to investigate Dishevelled function in maintenance of planar polarity. We provide mechanistic insight into the hierarchical relationship of Dishevelled with other members of the core planar polarity complex. Notably, we show that removal of Dishevelled in one cell causes rapid release of Prickle into the cytoplasm in the neighboring cell. This release of Prickle generates a self-propagating wave of planar polarity complex destabilization across the tissue. Thus, Dishevelled actively maintains complex integrity across intercellular junctions.

## Highlights

• Inducible genetic tools can efficiently disrupt Dishevelled activity *in vivo*
• Dishevelled activity continuously promotes core planar polarity complex stability
• Prickle is maintained in intercellular complexes cell non-autonomously by Dishevelled
• Unbound Prickle results in intercellular propagation of complex destabilization

## Graphical Abstract

**Figure:**
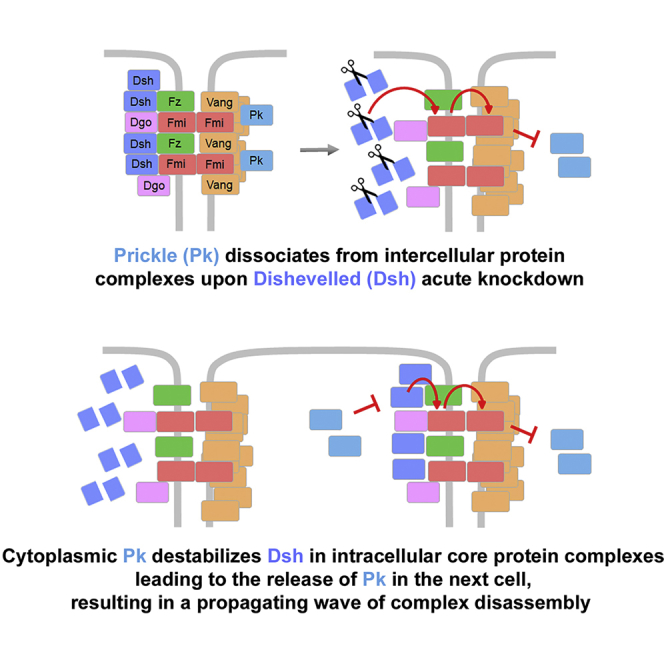

## Teaser

Ressurreição et al. have developed tools to rapidly disrupt protein function in *Drosophila* and used these to investigate the function of the protein Dishevelled in planar polarity. They find a continuous requirement for Dishevelled in core planar polarity complex maintenance with its loss causing relocalization of Prickle in neighboring cells.

## Introduction

Planar polarity is the coordination of cell polarity within the plane of a tissue (Goodrich and Strutt, 2011, Butler and Wallingford, 2017). It is most obviously manifested by the orientation of trichomes and bristles in *Drosophila* or hair structures in the inner ear and skin of vertebrates (Goodrich and Strutt, 2011, Devenport, 2016). Importantly, disruptions in planar polarity have been linked to congenital birth defects and cancer (Butler and Wallingford, 2017).

**Figure 1:**
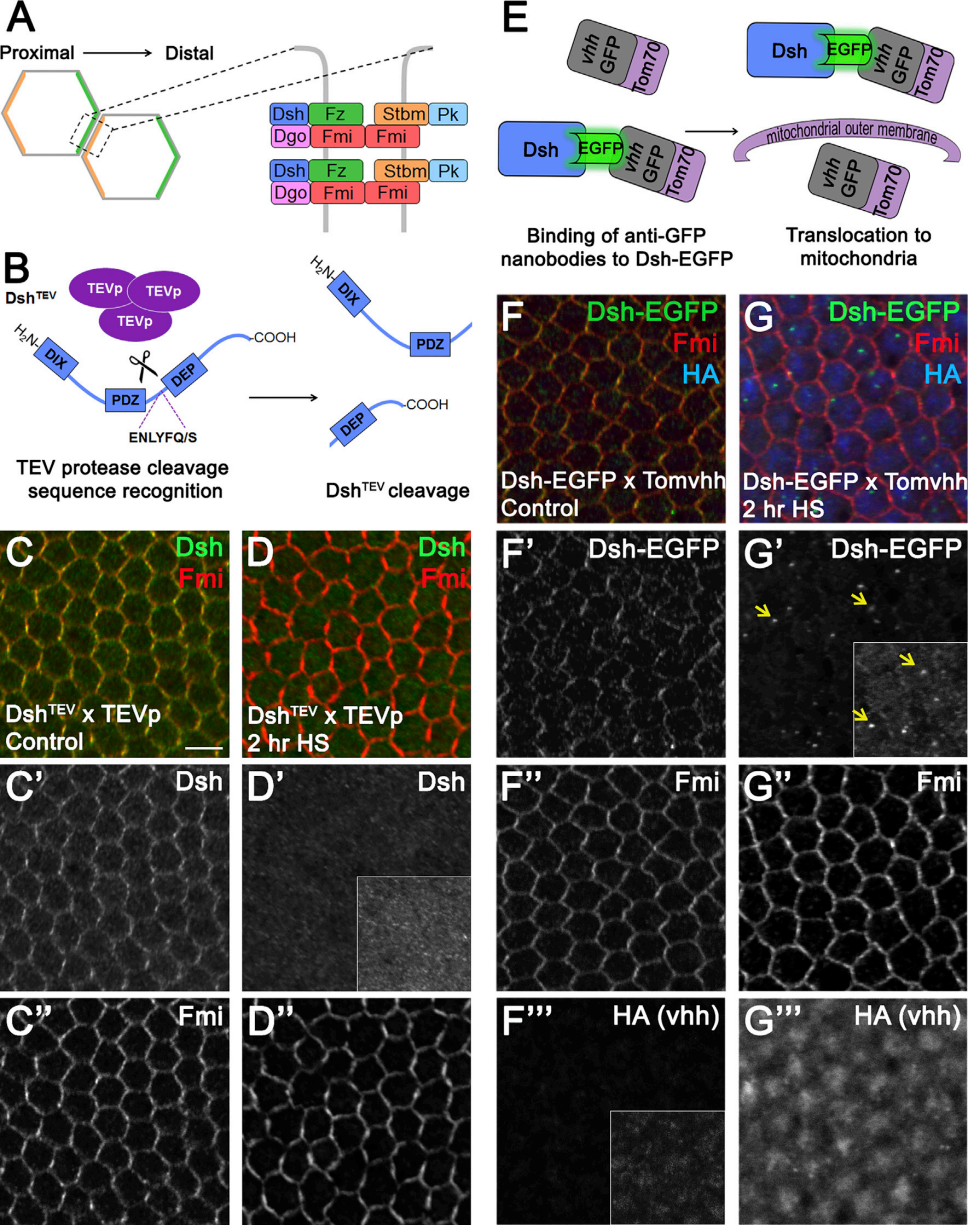
*In Vivo* Disruption of Dishevelled in the *Drosophila* Pupal Wing (A) Graphical representation of cells of the wing epithelium depicting the asymmetric distribution of the “core” planar polarity proteins. (B) Schematic representation of Dsh^TEV^ disruption by heat shock-induced TEVp-induced cleavage at introduced TEVp cleavage sites. (C and D) Twenty-eight hours APF wing epithelium heterozygous for *dsh*^*TEV*^ and *hs-TEVp* transgenes in a *dsh*^*V26*^-null background. (C) In the absence of heat-shock, no *hs-TEVp* is expressed, and the *dsh*^*TEV*^ transgene rescues the *dsh*-null phenotype, shown by the asymmetric localization of Dsh^TEV^ (green, C′) and endogenous Fmi (red, C″). (D) After expression of TEVp by a 2 hr heat shock at 38°C, Dsh^TEV^ localization is absent from the cell membrane (D′), but Fmi localization is maintained (D″). Scale bar, 5 μm. (E) Dsh-EGFP disruption on the basis of targeting with anti-GFP nanobodies fused to the Tom70 mitochondrial translocation signal. Upon Tom70-HA-vhhGFP production via heat shock, Dsh-EGFP is displaced from cell junctions. (F and G) Twenty-eight hr APF wing epithelium heterozygous for *dsh-EGFP* and *hs-Tom70-HA-vhhGFP* in a *dsh*^*V26*^-null background. (F) In the absence of heat shock, Dsh-EGFP (green, F′) and Fmi (red, F″) are asymmetrically localized, and there is no detection of Tom70-HA-vhhGFP by immunolabelling (blue, F‴). (G) After expression of Tom70-HA-vhhGFP by a 2 hr heat shock at 38°C, Dsh-EGFP disappears from the cell membrane and is seen in punctate cytoplasmic spots (yellow arrows, G′), while asymmetric Fmi labeling is maintained at the cell membrane (G″). Tom70-HA-vhhGFP signal (blue) is strongly detected by immunolabelling and is restricted to the cytoplasmic region of the cells (G‴). Insets in (D′), (F‴) and (G′) are increased intensity regions. See also Figure S1.

At the molecular level, planar polarity is defined as the asymmetric subcellular distribution of planar polarity proteins. During *Drosophila* wing development, the six proteins of the “core” planar polarity pathway (“core proteins” hereafter) self-organize along the proximodistal axis into stable asymmetric intercellular complexes (Figure 1A) of variable stoichiometry (Strutt et al., 2016). The transmembrane protein Frizzled (Fz) and the cytoplasmic proteins Dishevelled (Dsh) and Diego (Dgo) co-localize at distal junctions, while the fourpass transmembrane protein Strabismus (Stbm; also known as Van Gogh [Vang]) and the LIM-domain protein Prickle (Pk) co-localize proximally. The atypical cadherin Flamingo (Fmi; also known as Starry Night [Stan]) localizes both proximally and distally, bridging the two halves of the complex (reviewed in Strutt and Strutt, 2009). For intercellular complexes to form and distribute to opposite cell ends, activity of all six core proteins is required to enable feedback interactions that are thought to amplify cellular asymmetry (Strutt and Strutt, 2009, Warrington et al., 2017).

Dsh (Dvl in mammals) is a multifunctional protein that is a key cytoplasmic component of both the core planar polarity and Wnt signaling pathways and consequently has been intensely studied. Nevertheless, key questions remain regarding its cellular functions, and in particular its role in planar polarity feedback regulation is not well understood.

Although classical loss- and gain-of-function approaches were key in identifying the members of the core planar polarity complex, these techniques are not always suitable for dissecting molecular mechanisms, because of the effects of pleiotropy and adaptation (Hoeller et al., 2014, Warrington et al., 2017). To circumvent these limitations, technologies have been developed that can regulate *in vivo* protein function in a rapid and temporally controlled manner. These have proved successful in many biological systems including *Drosophila,* providing insights into developmental processes (Harder et al., 2008, Pauli et al., 2008, Caussinus et al., 2011, Warrington et al., 2017). In this study we optimized tools based on protein cleavage using tobacco etch virus protease (TEVp) (Harder et al., 2008, Pauli et al., 2008), and degradation or relocalization with anti-GFP nanobodies (Caussinus et al., 2011, Harmansa et al., 2017), to acutely disrupt Dsh activity *in vivo*.

## Results

### Disruption of Dsh Activity *In Vivo* in the *Drosophila* Pupal Wing Epithelium

We optimized two technologies based on either TEVp-induced cleavage or targeting by anti-GFP nanobodies (vhhGFPs) (Figures 1B, 1E, and S1B). The vhhGFPs were HA-tagged and fused to either the Tom70 import signal or Rpn10, which act to relocalize target proteins to the mitochondria (Robinson et al., 2010) or proteasome (Janse et al., 2004), respectively (Figures 1E and S1B). For TEVp-mediated knockdown, TEVp recognition sites were introduced into the Dsh coding sequence (Figure 1B). TEVp and the vhhGFP fusions were acutely expressed under control of the *hsp70* promoter in transgenic flies, via heat shock at 38°C (Figure S1A). In the absence of heat shock (control conditions), Dsh^TEV^ and Dsh-EGFP rescued the *dsh*-null phenotype, resulting in normal planar polarization (Figures 1C,1F, and S1C, compare with Figure S2J). As a further control, HA antibody labeling confirmed that the vhhGFP fusions were not expressed when pupae were maintained at 25°C in the absence of heat-shock (Figures 1F‴ and S1C‴). To exclude non-specific effects, *w*^*1118*^ flies were exposed to the same heat-shock regimes, and vhhGFPs and TEVp were produced in the absence of Dsh-EGFP or Dsh^TEV^, respectively (Table S1; Figures S3G–S3J); in all cases examined there was no significant change in core protein localization.

To rapidly disrupt Dsh^TEV^ via TEVp cleavage, we administered a 2 hr heat shock (38°C). Under these conditions and using an antibody that detects the C terminus of Dsh, Dsh^TEV^ localization at cell junctions was lost (Figures 1D and 1D′), while Fmi still remained asymmetrically localized at the cell membrane (Figure 1D″). This was accompanied by a significant loss in total cellular levels of Dsh protein, indicating that the cleaved protein was degraded (Figures S1E, S1F, and S2A).

Similarly, substantial depletion of Dsh-EGFP from junctions via Tom70-HA-vhhGFP and Rpn10-HA-vhhGFP expression was observed after 2 hr or 90 min heat shock, respectively (Figures 1G and S1D). HA labeling of Tom70-HA-vhhGFP was restricted to the cytoplasm (Figure 1G‴), while Dsh-EGFP was detected primarily in punctate spots within the cell (Figure 1G′, yellow arrows). However, Tom70-HA-vhhGFP did not reduce Dsh-EGFP total cellular levels (Figures S1E, S1F, and S4A), most likely because of sequestration of Dsh-EGFP to mitochondria. Upon production of Rpn10-HA-vhhGFP, Dsh-EGFP membrane localization was abolished, with HA labeling present throughout the cell (Figures S1D). This was accompanied by a significant loss in total cellular levels of Dsh-EGFP protein, indicating that the targeted protein is degraded (Figures S1E, S1F, and S4A).

We then compared the efficiency of depletion of Dsh using *UAS-TEVp* or *UAS-dsh-RNAi* (Figures S1A and S1G–S1L). Our attempts to knock down Dsh using RNAi were less efficient than the TEVp or vhhGFP methods (Figures S1G–S1J). Dsh was still detectable at cell junctions 6 hr after RNAi expression using two separate RNAi transgenes (Figures S1G–S1J). Furthermore, 4 hr of induced *GAL4/UAS* driven TEVp expression was not sufficient to abolish Dsh^TEV^ localization (Figures S1K and S1L).

### Targeted Disruption of Dsh Activity Differentially Affects the Transmembrane Core Complex Components

**Figure 2:**
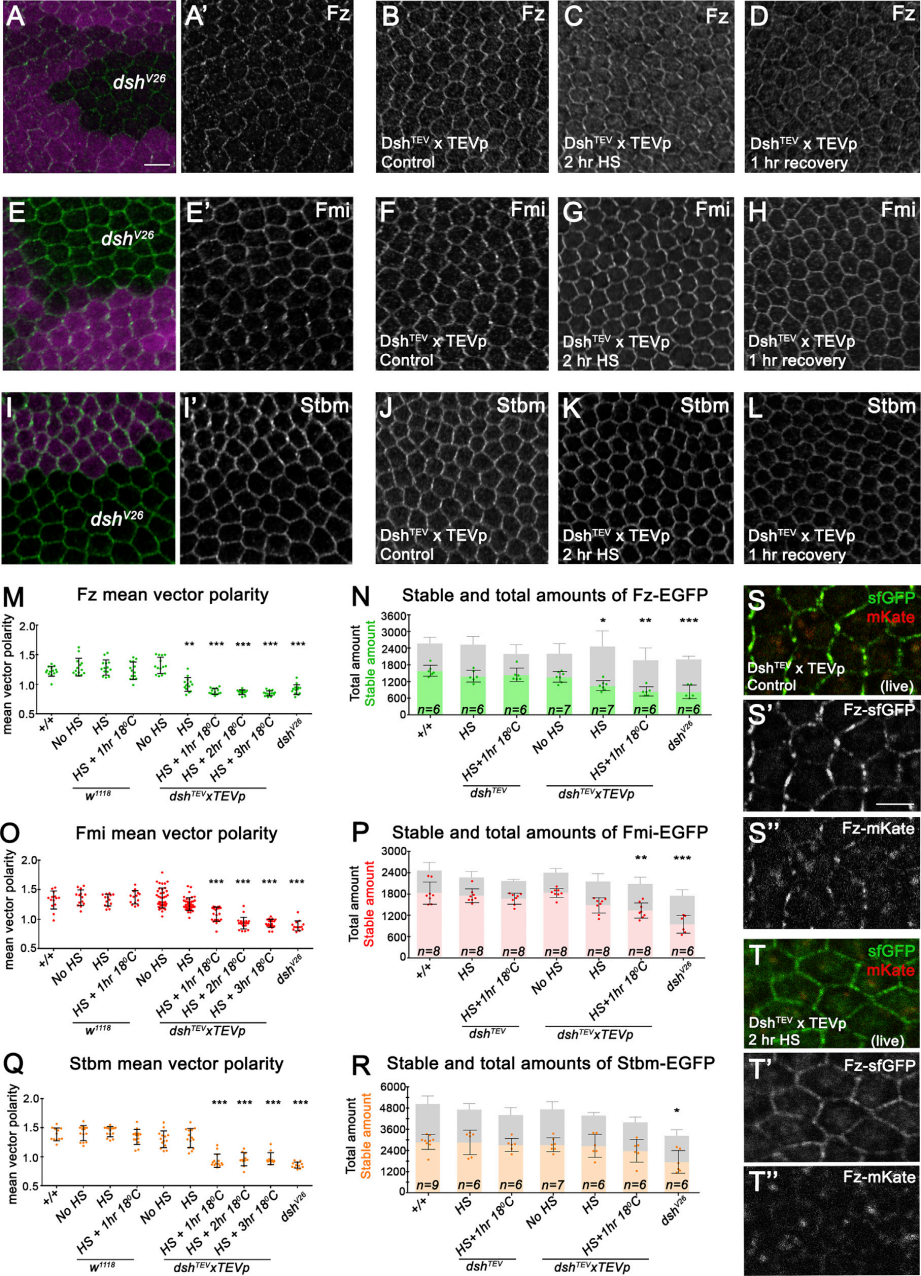
Dishevelled Activity Maintains the Asymmetry and Stability of the Transmembrane Core Planar Polarity Proteins (A, E, and I) Twenty-eight hr APF wing epithelia containing clones of *dsh*^*V26*^-null mutant tissue (loss of RFP, magenta), immunolabelled for endogenous Fz (A), Fmi (E), and Stbm (I). Scale bar, 5 μm and the same hereafter. (B–L) Twenty-eight hr APF wing epithelia showing localization of Fz (B–D), Fmi (F–H), or Stbm (J–L) before cleavage of Dsh^TEV^ (B, F, and J), immediately after (C, G, and K), and 1 hr after (D, H, and L). (M, O, and Q) Fz (M), Fmi (O), and Stbm (Q) polarity measurements before and after cleavage of Dsh^TEV^ and in *dsh*^*V26*^ and wild-type tissue. Error bars are SD; each data point represents one wing. (N, P, and R) FRAP experiments, showing stable amounts and total amounts of Fz-EGFP (N), Fmi-EGFP (P), or Stbm-EGFP (R) before and after cleavage of Dsh^TEV^. Error bars are SD; n, number of wings. For (M–R), ANOVA with Tukey-Kramer multiple-comparison test was used to compare all samples. Shown are comparisons of each experimental genotype to no heat-shock conditions and +/+ to *dsh*^*V26*^ clone tissue. ^∗^p ≤ 0.05, ^∗∗^p ≤ 0.01, and ^∗∗∗^p ≤ 0.001. See also Table S2. (S and T) Twenty-eight hr APF wing epithelium showing localization of Dsh^TEV^ in a *dsh*^*V26*^-null background, before and after cleavage by TEVp in the presence of the Fz “fluorescent timer” protein (Fz-mKate-sfGFP). (S) In the absence of heat-shock both “new” Fz (sfGFP) (S′) and “old” Fz (mKate) (S″) are asymmetrically localized at the junctions. (T) After a 2 hr heat shock, sfGFP is still detectable at the cell membrane and cytoplasm (T′), while mKate is reduced at the membrane (T″). See also Figure S2 and Tables S2 and S3.

After confirming that our techniques were able to remove Dsh protein from junctions within 2 hr, we went on to examine the effects on the polarity and stability of the transmembrane core proteins (Fz, Fmi, and Stbm). We examined their polarization prior to cleavage of Dsh^TEV^ (Figures 2B, 2F, and 2J), immediately after (Figures 2C, 2G, and 2K), and 1 hr after (Figures 2D, 2H, and 2L) and compared it with complete removal of *dsh* (Figures 2A, 2E, and 2I). Immunolabelling confirmed a normal Dsh asymmetric profile prior to disruption, and its absence from the cell junctions immediately after and 1 hr after (Figures S2P and S2R). Notably, total cellular levels or junctional levels of Fz, Fmi, or Stbm were not significantly altered (Figures S2B–S2D, S2N, S2P, and S2R). However, Fz polarity was significantly reduced immediately after Dsh disruption and continued to decrease thereafter, reaching levels similar to those in *dsh*-null tissue (Figure 2M). This was accompanied by a significant reduction in the stable amount of Fz, quantified using FRAP of Fz-EGFP (Strutt et al., 2016) (Figure 2N). To measure immediate effects on Fz turnover we also used a Fz “fluorescent timer” construct fused to superfolder GFP (sfGFP) and mKate chromophores, which discriminates between “newer” (sfGFP) and “older” (mKate) pools of Fz protein, because of the different maturation rates (Barry et al., 2016). Live imaging showed an increase in sfGFP and a decrease in mKate fluorescence at junctions upon Dsh deletion, confirming an increased Fz turnover (Figures 2S and 2T).

Fmi and Stbm responded more slowly to the absence of Dsh, as their polarization and stability were unchanged immediately after disruption (Figures 2O–2R; Table S1). However, 1 hr after Dsh disruption, polarity readouts for both proteins were significantly reduced (Figures 2O, 2Q; Table S1). Although Fmi stability levels were also significantly reduced 1 hr after knockdown (Figure 2P), the same was not observed for Stbm (Figure 2R). The changes in Fmi and Fz polarity were also recapitulated using Rpn10-HA-vhhGFP and Tom70-HA-vhhGFP (Figures S2F and S2G; Table S1). Imaging of Fmi at increased resolution immediately after knockdown showed an increased sparseness of Fmi containing puncta, suggesting a loosening of the complex (Figures S2H and S2I). Our findings reveal a hierarchical relationship between the transmembrane proteins, where Fz behavior is more tightly coupled to the presence of Dsh than that of Fmi and Stbm.

### Pk Translocates to the Cytoplasm upon Dsh Disruption, Independently of Dynamin Function

**Figure 3:**
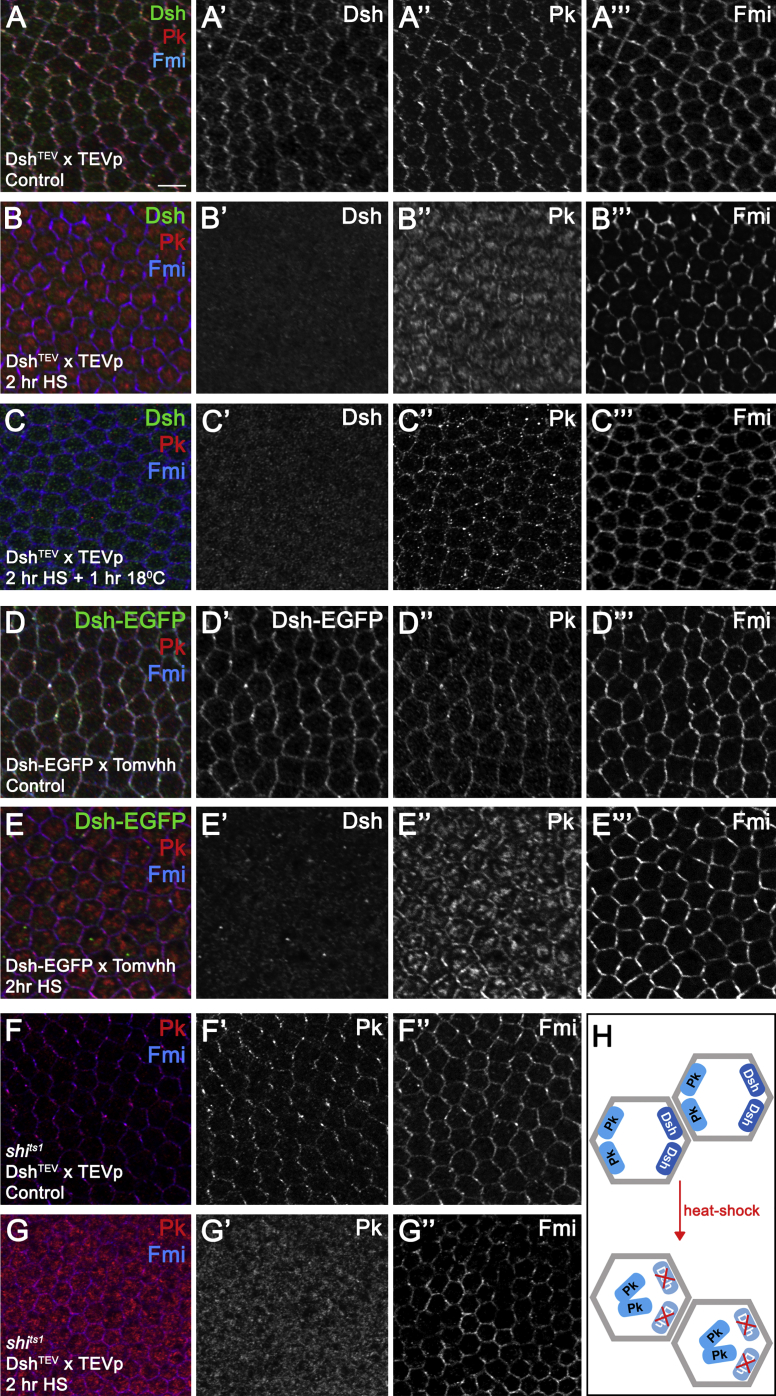
Dishevelled Disruption Leads to the Translocation of Prickle to the Cytoplasm (A–C) Twenty-eight hr APF wing epithelia showing Pk localization before (A), immediately after (B), or 1 hr after (C) Dsh^TEV^ cleavage, showing Pk (red), Dsh (green), and Fmi (blue). (B) Note the cytoplasmic fraction of Pk (B″) after Dsh^TEV^ cleavage, while junctional localization of Fmi is maintained (B‴). (C) One hr after Dsh^TEV^ cleavage, Pk shows spotty junctional localization (C″), negligible junctional Dsh is detected (C′), and Fmi shows reduced asymmetry (C‴). Scale bar, 5 μm. (D and E) Twenty-eight hr APF wing epithelia expressing Dsh-EGFP in a *dsh*^*V26*^-null background before (D) and after (E) sequestration of Dsh-EGFP using *hs-Tom70-HA-vhhGFP*. (D) In the absence of heat-shock Dsh-EGFP (D′), Pk (D″), and Fmi (D‴) localize asymmetrically. (E) Sequestration of Dsh-EGFP leads to Dsh-EGFP disappearance from the cell junctions (E′), Pk localization to the cytoplasm (E″), while Fmi maintains its asymmetry (E‴). (F and G) Effect of cleavage of Dsh^TEV^ using *hs-TEVp* in a *dsh*^*1*^*shi*^*ts1*^ background. (F) At 18°C, Pk (F′) and Fmi (F″) are asymmetrically localized. (G) Immediately after 2 hr heat shock at 38°C, Dsh^TEV^ cleavage still results in Pk localization to the cytoplasm (G′) even though Dynamin-dependent endocytosis should be blocked under these conditions, while Fmi remains membrane associated (G″). (H) Schematic representation depicting the relocation of Pk after Dsh disruption. See also Figure S3.

Pk and Dgo showed the expected junctional asymmetric localization prior to disruption of Dsh (Figures 3A, 3D, S2N, and S3A). After Dsh disruption, Dgo remained associated with the cell membrane (Figure S2O). This phenotype is different from that observed in *dsh*-null clones, where the Dgo levels at the cell membrane are strongly reduced (Figures S2L and S2M).

Strikingly, immediately after Dsh disruption, Pk moves to the cytoplasm (Figures 3B, 3E, 3H, and S3B). This Pk translocation is dynamic, recovering 1 hr after Dsh disruption (Figure 3C), when a spotty symmetric membrane association can be detected, similar to that detected in *dsh*-null tissue (Figures S2K and S2U). We hypothesize that the rapid recovery of Pk localization to junctions may be because Pk contains a prenylation motif that allows it to associate with membranes independently of core protein complexes. Overall cellular levels of Pk did not change significantly (Figures S2E).

We then asked if Pk translocation is mechanistically dependent on endocytosis, as Pk is known to destabilize Fz-EGFP by endocytic mechanisms (Warrington et al., 2017). Interestingly, blocking endocytosis alone using *shi*^*ts1*^ at 29°C partially disrupts localization of Pk, but not the other core proteins, although Pk still largely maintains its asymmetry at junctions (Figures S3K–S3O). Induction of Dsh acute knockdown concomitantly with blocking of endocytosis in the pupal wing does not prevent translocation of Pk to the cytoplasm, suggesting an endocytosis-independent mechanism (Figures 3F, 3G, S3C, and S3D). Moreover, cytoplasmically relocalized Pk does not co-localize with the endosome marker Rab5 or with the nuclear marker DAPI (Figures S3E and S3F).

### Pk Translocation into the Cytoplasm Produces a Propagating Wave of Destabilization of Core Protein Complexes

**Figure 4:**
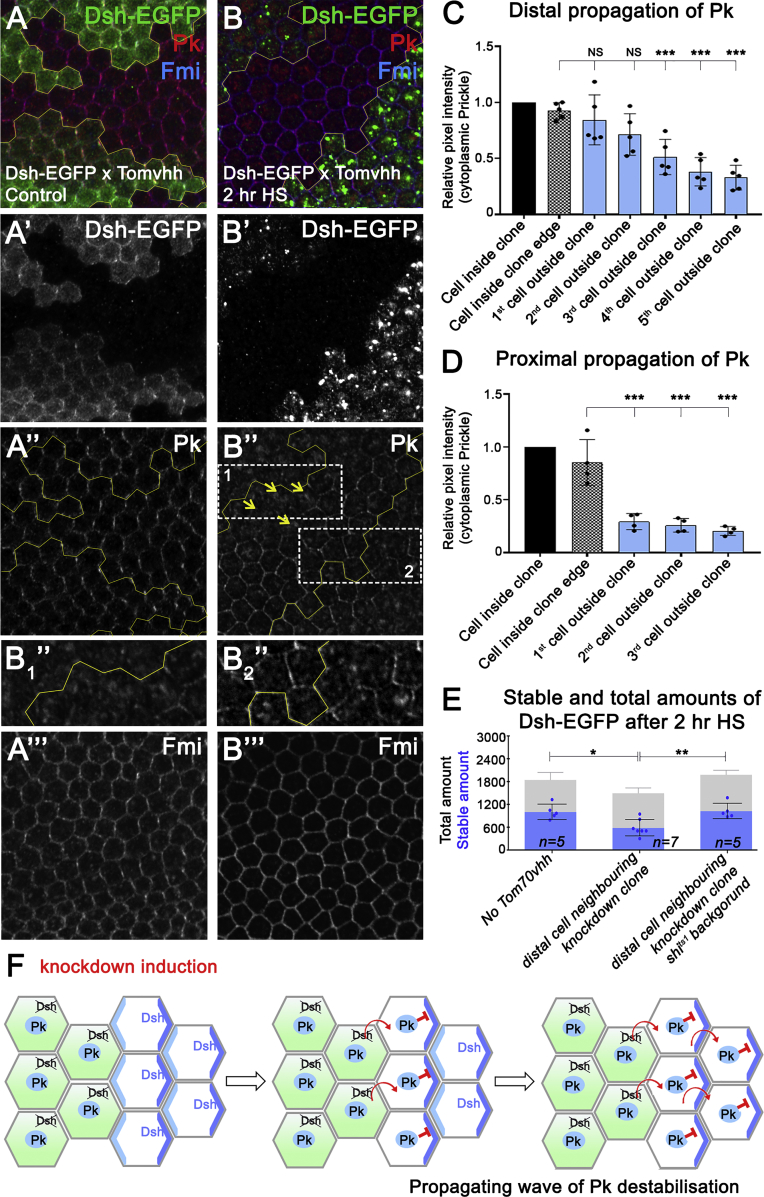
Cytoplasmic Translocation of Pk Results in a Propagating Wave of Destabilization of Core Protein Complexes (A and B) Twenty-eight hr APF pupal wings carrying clones of Dsh-EGFP, in a *dsh*^*V26*^-heterozygous background, with Tom70-HA-vhhGFP expressed in all cells (clone boundaries marked by yellow lines). (A) In the absence of heat shock, Dsh-EGFP (A′), Pk (A″), and Fmi (A‴) are all asymmetrically localized inside (and outside in the case of Pk and Fmi) Dsh-EGFP clones. (B) Immediately after heat shock, Dsh-EGFP in clones becomes punctate and is absent from the cell membranes (B′). Pk localization (B″) becomes cytoplasmic inside clones (white rectangle 1, inset B_1_″) and propagates distally to cells outside of the Dsh-EGFP expressing tissue (yellow arrows), where Dsh is not targeted by vhhGFP expression. Pk localization remains junctional proximal to Dsh-EGFP clones (white rectangle 2, inset B_2_″). Fmi remains associated with the cell junctions (B‴). Scale bar, 5 μm. (C and D) Quantification of cytoplasmic Pk inside Dsh-EGFP clone knockdown tissue and its propagation through cells distal (C) or proximal (D) to a clone. Error bars are SD; each data point represents one wing. ANOVA with Dunnett’s multiple comparison test was used to compare the first cell inside the clone edge (column 2) with all other samples; ^∗∗∗^p ≤ 0.001. (E) Dsh-EGFP stable amounts determined by FRAP analysis, showing control cells (left column), cells lacking Tom70-vhhGFP that are immediately distal to cells in which Dsh-EGFP has been sequestered by Tom70-HA-vhhGFP (middle column, heterozygous for Dsh-EGFP and Tom70-vhhGFP), and similar cells that are also hemizygous for *shi*^*ts1*^ and thus should be inhibited for Dynamin-dependent endocytosis (right column). Error bars are SD; n, number of wings. ANOVA with Tukey-Kramer multiple-comparison test was used to compare all genotypes; ^∗^p ≤ 0.05 and ^∗∗^p ≤ 0.01. See also Table S2. (F) Schematic representation of Pk release upon Dsh disruption in clonal tissue and the further destabilization of Dsh by cytoplasmic Pk creating a propagating wave of polarity destabilization. See also Figure S4 and Table S2 and S3.

To understand how Dsh affects Pk junctional localization, we used mosaic analysis to determine if the effect was cell autonomous or non-autonomous. We generated clones of Dsh-EGFP expression in a background uniformly heterozygous for the *hs-Tom70-HA-vhhGFP* transgene (Figures 4A and 4B). Even though endogenous Dsh is present in addition to Dsh-EGFP, Pk translocation to the cytoplasm still occurs after heat shock-induced expression of Tom70-HA-vhhGFP (Figure 4B″; note that endogenous Dsh levels do not change; see Figure S4A). Notably, targeting of Dsh-EGFP with Tom70-HA-vhhGFP resulted in a dominant and cell-non-autonomous effect on Pk junctional localization, such that Pk also translocates to the cytoplasm in adjacent tissue lacking Dsh-GFP (Figure 4B″). The translocation of Pk into the cytoplasm propagates distally (but not proximally) away from the Dsh-EGFP tissue (Figures 4B–4D), while junctional levels of Pk in cells proximal to Dsh-EGFP knockdown tissue are similar to wild-type levels (Figure S4B). Importantly, the distal propagation of Pk release from complexes supports a model in which Dsh localized at the distal edge of one cell non-autonomously promotes Pk localization in intercellular complexes at the proximal edge of the juxtaposed neighboring cell (see Figure 4F).

To further corroborate the ability of Dsh to cell-non-autonomously influence Pk even in the absence of rapid disruption, we carried out FRAP on boundaries of null mutant *dsh* clones. We found that EGFP-Pk on cell junctions inside Dsh expressing cells that neighbor *dsh*-mutant cells had significantly reduced stability, compared with EGFP-Pk on cell junctions inside *dsh*-mutant cells that neighbor cells with Dsh activity (Figures S4C–S4I). However, Pk did not translocate into the cytoplasm, and only mild effects on junctional levels of Pk were observed around *dsh* clones (Figure S4J). Taken together, we conclude that a Dsh-dependent signal passes across cell junctions to non-autonomously maintain Pk stability and localization in core protein intercellular complexes.

Notably, the distal propagation of cytoplasmic Pk release results in destabilization of Dsh-EGFP in core protein complexes in a Dynamin-dependent manner (Figure 4E). This is consistent with our previous observation that ectopic induction of Pk expression results in cell-autonomous destabilization of Fz-Dsh complexes in a process requiring Dynamin activity (Warrington et al., 2017).

Finally we asked whether the effect of Dsh disruption on Pk localization is an indirect consequence of changes in the cell cytoskeleton that might result from the removal of Dsh. We examined the distribution of F-actin, β-tubulin, and E-cadherin immediately after Dsh disruption (Figures S4K–S4M). No changes were evident in the distribution or levels of these molecules. We also attempted to recapitulate the release of Pk from cell junctions by perturbing the actin and tubulin cytoskeletons. Neither pharmacological reduction of F-actin with latrunculin A nor genetic disruption of microtubule density via KLP10A and Katanin co-expression altered Pk membrane association (Figures S4N–S4S).

Overall we have shown that Dsh positively regulates Pk localization in core protein intercellular complexes in a cell-non-autonomous manner. Moreover, Pk that becomes relocalized to the cytoplasm is capable of destabilizing Dsh in a Dynamin-dependent manner, leading to a wave of destabilization of core protein complexes across the tissue (Figure 4F).

## Discussion

Defining the roles of individual components in signaling networks can be a significant challenge. This is particularly so when the network is not a simple linear pathway, if components play more than one role in the cell (pleiotropy), and if there is “adaptation” such that over time, the pathway adjusts to the effects of experimental manipulations. However, in many cases, these difficulties can be bypassed through methods that rapidly alter protein activities (Hoeller et al., 2014, Harmansa et al., 2017).

Consistent with this, we have recently shown that spatiotemporal activation of gene expression is an effective tool for dissecting feedback interactions during planar polarity patterning in the *Drosophila* wing (Warrington et al., 2017). In this work, we now use methods for rapidly disrupting protein function to probe the role of the Dsh protein in planar polarity.

Our main finding is that Dsh regulates Pk membrane association in core planar polarity complexes, acting cell-non-autonomously to prevent its relocalization to the cytoplasm. Notably, this role for Dsh is specifically revealed when Dsh is rapidly depleted from core protein complexes but not in the simple *dsh* loss-of-function situation, when instead a largely mobile fraction of Pk is seen associated with cell junctions. We speculate that a Dsh-dependent signal normally passes between cells via the core protein complexes to maintain Pk recruitment. When this signal is disrupted, Pk rapidly leaves the junctions. However, in the long-term absence of Dsh, Pk can return to cell junctions, where we speculate it weakly associates with cell membranes by virtue of being prenylated (Strutt et al., 2013b).

What might be the nature of the intercellular signal from Dsh to Pk? We suggest it passes via the Fmi homodimers that form between cells, as numerous lines of evidence indicate these are essential for cell-cell signaling in planar polarity (reviewed in Strutt and Strutt, 2009). A simple possibility is that Dsh binding to Fz induces a conformational change in the complex that passes via the Fmi homodimers to alter the conformation of bound Stbm, thus creating a Pk binding site. Such molecular signaling events mediated by allostery are common features of ligand-receptor interactions (Nussinov et al., 2013). In support of the model that the Dsh signal is transduced via a change in Fz behavior, we note that following Dsh disruption, Fz distribution and stability is altered faster than those of Fmi and Stbm (Figure 2).

A related mechanism is suggested by our recent observations that the core proteins incorporate into intercellular complexes non-stoichiometrically and that all components contribute to complex stability (Strutt et al., 2016, Warrington et al., 2017). We interpret these findings as suggesting that core complex stability is dependent on a phase transition mediated by multivalent interactions between the core proteins, with Dsh playing a critical role (Warrington et al., 2017). The rapid destabilization of Fz after Dsh depletion may be a result of loss of multivalent binding interactions mediated between the different domains of Dsh, as also occurs in Wnt signalosome assembly (Gammons and Bienz, 2018), over time leading to a reduction in multivalent binding interactions between Fmi and Stbm. This would thus produce a gradual “loosening” of the complex that would result in release of Pk from its binding interactions with Fmi and Stbm. Some support for this model comes from our observation that super-resolution microscopy immediately after Dsh disruption shows subtle changes in the size and distribution of Fmi in junctional puncta (Figure S2I).

A striking observation is that if Dsh fails to maintain Pk recruitment in intercellular complexes, free Pk can destabilize Dsh in the same cell, leading to release of Pk in the neighboring cell and a wave of core planar polarity complex destabilization (Figures 4B and 4F). This observation both supports our previous work showing that physiological levels of Pk can effectively destabilize Fz-Dsh complexes at cell junctions (Warrington et al., 2017) and highlights the importance of sequestering Pk into “proximal” complexes, to prevent unregulated activity of Pk within the cell. We propose that Fmi mediates essential intercellular signals from Fz-Dsh in “distal” core complexes that actively maintain Pk in “proximal” core protein complexes. In turn, this promotes the effective segregation of distal and proximal complexes to opposite cell ends, driven in part by destabilizing feedback interactions between Pk and Dsh in the same cell.

## STAR★Methods

### Key Resources Table

**Table:** 

REAGENT or RESOURCE	SOURCE	IDENTIFIER
**Antibodies**		

Rabbit anti-GFP, affinity purified	Abcam	cat#ab6556; RRID: AB_305564
Mouse monoclonal anti-β-actin AC-40	Sigma-Aldrich	cat#A4700; RRID: AB_476730
Rat monoclonal anti-HA 3F10	Sigma-Aldrich	cat#12158167001; RRID: AB_390918
Mouse anti-β-tubulin E7 s	DSHB	RRID: AB_2315513
Mouse monoclonal anti-Fmi #74	DSHB (Usui et al., 1999)	RRID: AB_2619583
Rat anti-Pk, affinity purified	David Strutt (Strutt et al., 2013a)	N/A
Rabbit anti-Dsh, affinity purified (western blotting)	David Strutt (Strutt et al., 2006)	N/A
Rat anti-Dsh (immunolabelling)	David Strutt (Strutt et al., 2006)	N/A
Rabbit anti-Stbm (western blotting)	Tanya Wolff (Rawls and Wolff, 2003)	N/A
Rabbit anti-Stbm (immunolabelling)	David Strutt (Warrington et al., 2013)	N/A
Rat anti-Stbm (immunolabelling)	David Strutt (Strutt and Strutt, 2008)	N/A
Rabbit anti-Fz, affinity purified	David Strutt (Bastock and Strutt, 2007)	N/A
Rat anti-E-Cad	DSHB (Oda et al., 1994)	N/A
Rabbit anti-Rab5	Abcam	cat#ab13253; RRID: AB_2569809

**Chemicals, Peptides, and Recombinant Proteins**		

16% paraformaldehyde solution (methanol free)	Agar Scientific	cat#R1026
Phalloidin Alexa-568	ThermoFisher	cat#A12380
NucBlue (DAPI)	ThermoFisher	cat#R37605
Triton X-100	VWR	cat#28817.295; CAS: 9002-93-1
Tween-20	VWR	cat#437082Q; CAS: 9005-64-5
Methyl cellulose	Sigma-Aldrich	cat#M0387; CAS: 9004-67-5
Latrunculin A	ThermoFisher	cat#L12370; CAS: 76343-93-6
DMSO	Sigma-Aldrich	cat#D9170; CAS: 67-68-5
Schneider’s medium	ThermoFisher	cat#21720-024
Glycerol	VWR	cat#284546F; CAS: 56-81-5
DABCO	Fluka	cat#33480; CAS: 280-57-9
Normal Goat Serum	Jackson Labs	cat#005-000-121
Halocarbon 700 Oil	Halocarbon Products Corp.	CAS: 9002-83-9

**Experimental Models: Organisms/Strains**		

*stbm*^*6*^	Tanya Wolff (Wolff and Rubin, 1998)	FlyBase: FBal0062423
*dsh*^*V26*^	Norbert Perrimon (Perrimon and Mahowald, 1987)	FlyBase: FBal0003140
*dsh*^*1*^	Bloomington Drosophila Stock Center	FlyBase: FBal0003138; RRID: BDSC_5298
*dgo*^*380*^	Suzanne Eaton (Feiguin et al., 2001)	FlyBase: FBal0141190
*pk*^*pk-sple13*^	David Gubb (Gubb et al., 1999)	N/A
*shi*^*ts1*^	Bloomington Drosophila Stock Center	FlyBase: FBal0015610; RRID: BDSC_7068
*fz-EGFP*	David Strutt (Strutt et al., 2016)	N/A
*EGFP-pk*	David Strutt (Strutt et al., 2016)	N/A
*fmi-EGFP*	David Strutt (Strutt et al., 2016)	N/A
*P[acman]-dsh-EGFP*	This paper	N/A
*P[acman]-stbm-EGFP*	David Strutt (Strutt et al., 2016)	N/A
*P[acman]-EGFP-dgo*	David Strutt (Strutt et al., 2016)	N/A
*P[acman]-dsh*^*3xTEV*^	This paper	N/A
*P[CaSpeR]-hs-Tom70-HA-vhhGFP*	This paper	N/A
*P[CaSpeR]-hs-Rpn10-HA-vhhGFP*	This paper	N/A
*ActP-FRT-polyA-FRT-fz-mKate-sfGFP*	This paper	N/A
*P[CaSpeR]-hs-TEVp*	Reinhard Schuh (Harder et al., 2008)	N/A
*pUAS-TEVp*	Reinhard Schuh (Harder et al., 2008)	N/A
*pUAS-KLP-10A, pUAS-Katanin-60*	Christian Dahmann (Widmann and Dahmann, 2009)	N/A
*pUAS-dshNIG*^*18361R-2*^	National Institute of Genetics	Stock ID: 18361R-2 NIG078563.2
*pUAS-dsh*^*WIZ*^	David Strutt (Bastock and Strutt, 2007)	N/A
*hs-FLP*	Bloomington Drosophila Stock Center	FlyBase: FBti0002044; RRID: BDSC_6
*Ubx-FLP*	Bloomington Drosophila Stock Center	FlyBase: FBti0150334; RRID: BDSC_42718
*Actin-GAL4*	Bloomington Drosophila Stock Center	FlyBase: FBti0012293; RRID: BDSC_4414
*tubulin-GAL80*^*ts*^	Bloomington Drosophila Stock Center	FlyBase: FBti0027796; RRID: BDSC_7019
*Ubi-mRFP-nls*	Bloomington Drosophila Stock Center	FlyBase: FBti0129786; RRID: BDSC_30852

**Software and Algorithms**		

NIS Elements AR version 4.60	Nikon	N/A
Image Lab version 4.1	BioRad Laboratories	N/A
ImageJ version 2.0.0	https://fiji.sc	N/A
MATLAB_R2014b	Mathworks	N/A
GraphPad Prism version 7.0c	GraphPad Software, Inc.	N/A
G^∗^Power version 3.1	http://www.gpower.hhu.de	N/A
Tissue Analyzer	https://grr.gred-clermont.fr/labmirouse/software/WebPA/	N/A
Polarity measurement scripts (MATLAB)	David Strutt (Strutt et al., 2016)	N/A

### Contact for Reagent and Resource Sharing

Further information and requests for resources and reagents should be directed to and will be fulfilled by the Lead Contact, David Strutt (d.strutt@sheffield.ac.uk).

### Experimental Model and Subject Details

*Drosophila melanogaster* flies were grown on standard cornmeal/agar/molasses media at 18°C or 25°C, unless otherwise described.

### Method Details

#### Molecular Biology

*P[acman]-dsh-EGFP* and *P[acman]-dsh*^*3xTEV*^ were generated from *P[acman]-dsh* (Strutt et al., 2016) via recombineering (Wang et al., 2009). *P[acman]-dsh-EGFP* is a fusion of EGFP to the C terminus of Dsh, made as previously described (Strutt et al., 2016), and for *P[acman]-dsh*^*TEV*^ three TEVp sites were inserted between the codons encoding amino acids 379-380. *ActP-FRT-polyA-FRT-fz-sfGFP-mKate*, is a fusion of sfGFP and mKate to the C terminus of Fz. *P[CaSpeR]-hs-Tom70-HA-vhhGFP* and *P[CaSpeR]-hs-Rpn10-HA-vhhGFP* are fusions of amino acids 1-50 of *Drosophila Tom70* or the full-length *Drosophila Rpn10* coding sequence respectively to the *vhhGFP* coding sequence, separated by a linker containing a single HA-tag sequence. Full sequences and cloning details are available upon request.

#### Fly genetics

Fly strains are described in the Key Resources Table. Mutant alleles are described in FlyBase. *stbm*^*6*^, *dsh*^*V26*^ *pk*^*pk-sple13*^ and *dgo*^*380*^ are null alleles and unable to give rise to functional proteins; *dsh*^*1*^ contains a missense mutation in the DEP domain which has been reported to be a strong mutation for planar polarity activity. *shi*^*ts1*^ is a thermosensitive Dynamin mutation which allows normal endocytosis to occur at the permissive temperature (18°C) and is less active at the restrictive temperature (29°C).

*P[acman]* constructs were integrated into the genome via ΦC31-mediated recombination into the *attP40* landing site. For the acute knockdown of Dsh we used genomic rescue constructs *P[acman]-dsh*^*3xTEV*^ and *P[acman]-dsh-EGFP*, and genetically encoded effector transgenes expressed under heat-shock promoter control *pCaSpeR-hs-TEVp* (Harder et al., 2008), *pCaSpeR-hs-Tom70-HA-vhhGFP* and *pCaSpeR-hs-Rpn10-HA-vhhGFP*. Two *UAS-dsh-RNAi* transgenes (*dsh*^*NIG18361R-2*^ [RNAi1] and *dsh*^*WIZ*^ [RNAi2] (Bastock and Strutt, 2007)) and a *UAS-TEVp* (Harder et al., 2008) line were conditionally expressed using *Act-GAL4, tub-GAL80*^*ts*^. Mitotic clones were produced using the FLP/FRT system with *Ubx-FLP* or *hs-FLP*. The fluorescent timer transgene *ActP-FRT-polyA-FRT-fz-sfGFP-mKate* was used to study Fz turnover. FRAP experiments used knock-ins of *EGFP-pk*, *fmi-EGFP* and *fz-EGFP* or genomic rescue constructs for *P[acman]-stbm-EGFP* and *P[acman]-EGFP*-*dgo,* in *stbm*^*6*^ or *dgo*^*380*^ mutant backgrounds respectively (Strutt et al., 2016). *UAS-KLP10A* and *UAS-Katanin-60* were used to depolymerize and sever microtubules (Widmann and Dahmann, 2009). Transgenics were generated by Genetivision and BestGene.

Genotypes for experiments were:

Figure 1
(C-D) *dsh*^*V26*^*/Y; P[acman]-dsh*^*3xTEV*^*/P[CaSpeR]-hs-TEVp*
(F-G) *dsh*^*V26*^*/Y; P[acman]-dsh-EGFP/P[CaSpeR]-hs-Tom70-HA-vhhGFP*
Figure 2
(A,E,I) *dsh*^*V26*^ *FRT19A/ubi-mRFP-nls FRT19A; Ubx-FLP/+*
(B-D, F-H and J-L) *dsh*^*V26*^*/Y; P[acman]-dsh*^*3xTEV*^*/P[CaSpeR]-hs-TEVp*
(M, O and Q) w^1118^
*dsh*^*V26*^*/Y; P[acman]-dsh*^*3xTEV*^*/P[CaSpeR]-hs-TEVp*
*dsh*^*V26*^ *FRT19A/ ubi-mRFP-nls FRT19A; Ubx-FLP/+*
(N) *w*^*1118*^*; fz-EGFP/+*
*dsh*^*V26*^*/Y; P[acman]-dsh*^*3xTEV*^*/+; fz-EGFP/+*
*dsh*^*V26*^*/Y; P[acman]-dsh*^*3xTEV*^*/P[CaSpeR]-hs-TEVp; fz-EGFP/+*
*dsh*^*V26*^ *FRT19A/ ubi-mRFP-nls FRT19A; Ubx-FLP/+; fz-EGFP/+*
(P) *w*^*1118*^*; fmi-EGFP/+*
*dsh*^*V26*^*/Y; P[acman]-dsh*^*3xTEV*^*/fmi-EGFP*
*dsh*^*V26*^*/Y; P[acman]-dsh*^*3xTEV*^*/fmi-EGFP; P[CaSpeR]-hs-TEVp/+*
*dsh*^*V26*^ *FRT19A/ubi-mRFP-nls FRT19A; Ubx-FLP/fmi-EGFP*
(R) *w*^*1118*^*; P[acman]-stbm-EGFP stbm*^*6*^*/+*
*dsh*^*V26*^*/Y; P[acman]-dsh*^*3xTEV*^*/P[acman]-stbm-EGFP stbm*^*6*^
*dsh*^*V26*^*/Y; P[acman]-dsh*^*3xTEV*^*/P[acman]-stbm-EGFP stbm*^*6*^*; P[CaSpeR]-hs-TEVp/+*
*dsh*^*V26*^ *FRT19A/ubi-mRFP-nls FRT19A; Ubx-FLP/P[acman]-stbm-EGFP stbm*^*6*^
(S-T) *Ubx-FLP, dsh*^*V26*^*/Y; P[acman]-dsh*^*3xTEV*^*/P[CaSpeR]-hs-TEVp; ActP-FRT-polyA-FRT-fz-sfGFP-mKate/+*
Figure 3
(A-C) *dsh*^*V26*^*/Y; P[acman]-dsh*^*3xTEV*^*/P[CaSpeR]-hs-TEVp*
(D-E) *dsh*^*V26*^*/Y; P[acman]-dsh-EGFP/P[CaSpeR]-hs-Tom70-HA-vhhGFP*
(F-G) *dsh*^*1*^*, shi*^*ts1*^*, hs-FLP/Y; P[acman]-dsh*^*3xTEV*^*/P[CaSpeR]-hs-TEVp*
Figure 4
(A-D) *UbxFLP, dsh*^*V26*^*/w*^*1118*^*; P[acman]-dsh-EGFP FRT40/FRT40; P[CaSpeR]-hs-Tom70-HA-vhhGFP/+*
(E) *UbxFLP, dsh*^*V26*^*/Y; P[acman]-dsh-EGFP FRT40/P[CaSpeR]-hs-Tom70-HA-vhhGFP FRT40*
*dsh*^*1*^*, shi*^*ts1*^*, hs-FLP/Y; P[acman]-dsh-EGFP FRT40/hs-Tom70-HA-vhhGFP4 FRT40*
Figure S1
(C-D) *dsh*^*V26*^*/Y; P[acman]-dsh-EGFP/P[CaSpeR]-hs-Rpn10-HA-vhhGFP*
(E-F) *dsh*^*V26*^*/Y; P[acman]-dsh*^*3xTEV*^*/P[CaSpeR]-hs-TEVp*
*dsh*^*V26*^*/Y; P[acman]-dsh-EGFP/P[CaSpeR]-hs-Tom70-HA-vhhGFP*
*dsh*^*V26*^*/Y; P[acman]-dsh-EGFP/P[CaSpeR]-hs-Rpn10-HA-vhhGFP*
(G-H) *w*^*1118*^*/Y; Act-GAL4, tub-GAL80*^*ts*^*/+; UAS-dsh*^*NIG18361R-2*^*/+*
(I-J) *w*^*1118*^*/Y; Act-GAL4, tub-GAL80*^*ts*^*/+; UAS-dsh*^*WIZ*^*/+*
(K-L) *dsh*^*V26*^*/Y; P[acman]-dsh*^*3xTEV*^*/pUAS-TEVp*
Figure S2
(A-E) *w*^*1118*^*/Y; +/P[CaSpeR]-hs-TEVp*
*dsh*^*V26*^*/Y; P[acman]-dsh*^*3xTEV*^*/P[CaSpeR]-hs-TEVp*
(F-G) *w*^*1118*^
*dsh*^*V26*^*/Y; P[acman]-dsh-EGFP/+*
*dsh*^*V26*^*/Y; P[acman]-dsh-EGFP/P[CaSpeR]-hs-Tom70-HA-vhhGFP*
*dsh*^*V26*^*/Y; P[acman]-dsh-EGFP/P[CaSpeR]-hs-Rpn10-HA-vhhGFP*
*dsh*^*V26*^ *FRT19A/ubi-mRFP-nls FRT19A; Ubx-FLP/+*
(H-I) *dsh*^*V26*^*/Y; P[acman]-dsh*^*3xTEV*^*/P[CaSpeR]-hs-TEVp*
(J-K) *dsh*^*V26*^ *FRT19A/ubi-mRFP-nls FRT19A; Ubx-FLP/+*
(L-M) *dsh*^*V26*^ *FRT19A/ubi-mRFP-nls FRT19A; Ubx-FLP/P[acman]-EGFP-dgo dgo*^*380*^
(N-O) *dsh*^*V26*^*/Y; P[acman]-dsh*^*3xTEV*^*/ P[acman]-EGFP-dgo dgo*^*380*^*; P[CaSpeR]-hs-TEVp/+*
(P-R) *dsh*^*V26*^*/Y; P[acman]-dsh*^*3xTEV/*^*P[CaSpeR]-hs-TEVp*
(S) *w*^*1118*^*; P[acman-EGFP-dgo dgo*^*380*^*/+*
*dsh*^*V26*^*/Y; P[acman]-dsh*^*3xTEV*^*/P[acman-EGFP-dgo dgo*^*380*^
*dsh*^*V26*^*/Y; P[acman]-dsh*^*3xTEV*^*/P[acman]EGFP-dgo dgo*^*380*^*;P[CaSpeR]-hs-TEVp/+*
(T) *w*^*1118*^*; EGFP-pk/+*
*dsh*^*V26*^ *FRT19A/ubi-nls-RFP FRT19A; Ubx-FLP/P[acman]EGFP-pk*
(U) *dsh*^*V26*^ *FRT19A/ubi-nls-RFP FRT19A; Ubx-FLP/P[acman]EGFP-pk*
Figure S3
(A-B) *dsh*^*V26*^*/Y; P[acman]-dsh-EGFP/P[CaSpeR]-hs-Rpn10-HA-vhhGFP*
(C-D) *dsh*^*1*^*, shi*^*ts1*^*, hs-FLP/Y; P[acman]-dsh-EGFP/P[CaSpeR]-hs-Tom70-HA-vhhGFP*
(E-F) *dsh*^*V26*^*/Y; P[acman]-dsh*^*3xTEV*^*/P[CaSpeR]-hs-TEVp*
(G) *w*^*1118*^
(H) *w*^*1118*^*; P[CaSpeR]-hs-TEVp /P[CaSpeR]-hs-TEVp*
(I) *w*^*1118*^*; P[CaSpeR]-hs-Tom70-HA-vhhGFP /P[CaSpeR]-hs-Tom70-HA-vhhGFP*
(J) *w*^*1118*^*; P[CaSpeR]-hs-Rpn10-HA-vhhGFP /P[CaSpeR]-hs-Rpn10-HA-vhhGFP*
(K-O) *shi*^*ts1*^*, hs-FLP/Y*
Figure S4
(A) *dsh*^*V26*^*/w*^*1118*^*; P[acman]-dsh-EGFP/P[CaSpeR]-hs-Tom70-HA-vhhGFP*
*dsh*^*V26*^*/w*^*1118*^*; P[acman]-dsh-EGFP/P[CaSpeR]-hs-Rpn10-HA-vhhGFP*
(B) *UbxFLP, dsh*^*V26*^*/w*^*1118*^*;P[acman]-dsh-EGFPFRT40/FRT40;P[CaSpeR]-hs-Tom70-HA-vhhGFP/+*
(C-F, I) *dsh*^*V26*^ *FRT19A/ubi-mRFP-nls hs-FLP FRT19A; FRT42 EGFP-Pk/FRT42 pk-sple*^*13*^
(J) *dsh*^*V26*^ *FRT19A/ubi-mRFP-nls FRT19A; Ubx-FLP/+*
(K-M) *UbxFLP, dsh*^*V26*^*/w*^*1118*^*;P[acman]-dsh-EGFPFRT40/FRT40;P[CaSpeR]-hs-Tom70-HA-vhhGFP/+*
(N) *w*^*1118*^*; Act-GAL4, tub-GAL80*^*ts*^*/+; UAS-KLP-10A, UAS-Katanin-60/+*
(O) *w*^*1118*^
(P-S) *w*^*1118*^*; EGFP-pk*

#### Temperature regimes for protein activity disruption

Pupae were aged for 28 hr at 25°C unless otherwise stated, except for genotypes with *hs-TEVp* on the third chromosome in which pupae were aged for twice as long at 18°C, as the transgene is leaky at 25°C. Heat-shocks were performed by placing pupae in plastic vials in a water bath at 38°C for up to 2 hr. Longer heat-shock regimes were not possible due to pupal lethality. Afterward, pupae were either immediately dissected or left to recover at 18°C for 1, 2 or 3 hr prior to use. From the same population control pupae were set aside, and aged to 28 hr APF without heat shock.

#### Immunostaining and antibodies

Dissection and staining procedures were performed as previously reported (Strutt et al., 2016). Briefly, pupae were fixed in 4% paraformaldehyde in PBS and wings removed. Dissected wings were fixed for 30-45 min at room temperature, depending on antibody combinations. Wings were blocked for 1 hr in PBS containing 0.2% Triton X-100 (PTX) and 10% normal goat serum prior to antibody incubation. Primary antibodies were incubated overnight at 4°C, and secondary antibodies either for 4 hr at room temperature or overnight at 4°C, in PTX with 10% normal goat serum, all washes were in PTX. After immunolabelling, wings were post-fixed in 2% paraformaldehyde in PTX for 15 min and mounted in 10% glycerol, 1xPBS, containing 2.5% DABCO (pH7.5). For super-resolution imaging, wings were mounted in Vectorshield. Primary antibodies for immunolabelling were affinity purified rabbit anti-GFP (ab6556, Abcam, UK), rat monoclonal anti-HA 3F10 (Sigma-Aldrich), affinity purified rat anti-Pk (Strutt et al., 2013b), rat anti-Dsh (Strutt et al., 2006), rabbit anti-Dsh (Strutt et al., 2013a), rat anti-Stbm (Strutt and Strutt, 2008), rabbit anti-Stbm (Warrington et al., 2013), mouse monoclonal anti-Fmi (Flamingo #74, DSHB (Usui et al., 1999)), affinity-purified rabbit anti-Fz (Bastock and Strutt, 2007), mouse anti-β-tubulin E7 (DSHB) and rat monoclonal anti-DE-cad (DSHB) (Oda et al., 1994). Actin was visualized using Alexa-568-conjugated phalloidin (Molecular Probes).

#### Western blotting

Pupal wings were processed for western blotting as previously described (Strutt et al., 2016). Briefly, pupal wings at 28 hr APF were dissected in 1x PBS, placed directly in 2x sample buffer on ice at a concentration of 1 wing per 10 μL and vortexed for 5 s and boiled at 95°C for 5 min to solubilise proteins. Afterward samples were vortexed for 10 s and stored at −20°C for use within a week. Before use, lysates were thawed, vortexed and 2 pupal wing equivalents were run on Tris-Bis precast gels (Invitrogen) and transferred using a wet apparatus onto a Hybond ECL nitrocellulose membrane (GE Healthcare). Membranes were blocked for 1 hr at room temperature in 5% skimmed milk in PBS containing 0.1% Tween-20. Primary antibodies were incubated overnight at 4°C and secondary antibodies at room temperature for 3 hr, in PBS containing 0.1% Tween-20 and 5% skimmed milk. Proteins were detected using affinity purified rabbit anti-Dsh antibody (Strutt et al., 2006), mouse monoclonal anti-Fmi #74 (DHSB, (Usui et al., 1999), affinity purified rabbit anti-Fz (Bastock and Strutt, 2007), rabbit anti-Stbm (Rawls and Wolff, 2003), affinity purified rat anti-Pk (Strutt et al., 2013a) or rat anti-β-actin AC-40 (Sigma-Aldrich), and HRP-conjugated secondary antibodies (DAKO). Detection was performed using Supersignal West Dura (Pierce) and a Bio-Rad ChemiDoc XRS+ was used for imaging. Band intensities from three separate experiments were quantified using ImageJ. Values were normalized to actin loading controls and then non-heat-shocked controls. Statistical analysis was carried out using unpaired t tests or one way-ANOVA.

#### Cytoskeleton disruption assays

Wings from *EGFP-pk* 6 hr APF prepupae were dissected in 1% PBS. To disrupt the actin cytoskeleton, a final concentration of 2μM Latrunculin A was added to the prepupal wings for 15 min. Wings were washed twice in PBS and mounted in methyl cellulose in Schneider’s medium, for live imaging within 5 min or stained for F-actin with Phalloidin-Alexa568. 6 hr APF prepupae were used as at this stage the developing wings are not enveloped by an impermeable cuticle. Severing of microtubules was performed by co-expression of *UAS-KLP10A* and *UAS-Katanin-60* (Widmann and Dahmann, 2009) at 25°C using *Act-Gal4, tub-Gal80*^*ts*^ and pupal wings dissected at 28 hr APF.

#### Heat-shock induction of tools to disrupt protein activity

All pupae were aged for 28 hr at 25°C unless otherwise stated. Heat-shocks were carried out in a water-bath at 38°C for up to 2 hr. Note that longer heat-shock regimes cause lethality. After heat-shock, pupae were either dissected, live-imaged, or left to recover at 18°C for 1, 2 or 3 hr. For experiments to block dynamin-dependent endocytosis, pupae hemizygous for *shi*^*ts1*^ were aged at 18°C, then heat-shocked for 2 hr.

#### Imaging

All confocal micrographs of fixed pupal wings were captured posterior to the L4 vein region of the pupal wing. Fixed pupal wings were imaged on a Nikon A1R GaAsP confocal microscope using a 60x NA1.4 apochromatic lens, with a pixel size of 70 nm, and a pinhole of 1.2 AU. 9 Z-slices separated by 150 nm were imaged, and then the 3 brightest slices around junctions were selected and averaged for each channel in ImageJ. Super-resolution imaging was carried out using a Zeiss LSM 710 AiryScan with a 63x lens.

For live imaging, white prepupae were collected and aged for 28 hr at 25°C (or the equivalent time at different temperatures (Warrington et al., 2017)). Briefly, a small piece of cuticle was removed from above the pupal wing, and the exposed wing was mounted in a drop of Halocarbon 700 oil in a glass-bottomed dish. For FRAP analysis, images were 256 × 256 pixels, with a pixel size of 100 nm, 60x 1.2 NA oil objective and a pinhole of 1.2 AU. For FRAP, regions of interest (ROIs) of ∼3 μm^2^ were selected as ellipses that covered 2/3 of proximal-distal membranes, where planar polarity proteins localize, with the exception of the experiment in Fig.S4I where any junctions on the boundary were bleached. Three prebleach images were taken at 2 frames/sec, and ROIs were then bleached using a 488 nm Argon laser at 85% with eight laser passes (one second total time), which resulted in 55%–70% bleaching. Immediately following bleaching, five images were taken at 5 s intervals, followed by 10 images at 10 s intervals, 10 images at 15 s intervals and 8 images at 30 s intervals. Laser power was adjusted to maintain constant power at the lens between different imaging sessions. When only EGFP was being imaged, a 488 nm laser and a long pass GFP filter were used. For samples expressing both EGFP and mRFP, a 488 nm laser and a 525-550 band pass filter were used to detect EGFP. After imaging, ROIs were manually reselected in ImageJ and quantitated, in addition to four unbleached regions to control for acquisition bleaching. Stable amounts were compared using ANOVA.

### Quantification and Statistical Analysis

#### FRAP processing

Data analysis was conducted as previously described (Strutt et al., 2011). Briefly, ImageJ was used to manually reselect up to 8 bleached regions in each image for each time point. The laser-off background was subtracted, and the values were then corrected for acquisition bleaching and normalized against the average of the prebleach values. Data were then plotted on an xy graph using Prism (v7 Graphpad), bleached regions within the same wing were averaged and a one-phase exponential curve was fitted for each wing. Multiple wings were then combined and a one phase exponential association curve was fitted. To determine the stable amount, the mean intensity of the ROIs from the three prebleach images was measured in ImageJ, and averaged per wing. The intensity was then corrected for distance from the coverslip as previously described (Strutt et al., 2016), and this value multiplied by the stable fraction (1-y[max]) for each wing. The stable amounts were then averaged across wings, and results were plotted on a scatter graph along with the mean and standard deviation. Statistical tests were performed using one-way ANOVA, with Dunnett’s multiple comparison test to compare a control to the rest of the genotypes in the experiment, Tukey-Kramer’s multiple comparison test to compare all genotypes within an experiment and the Holm-Šídák multiple comparison test for paired comparisons within an experiment. p values calculated in Prism 7 are reported on the graph as asterisks (^∗^ = p ≤ 0.5, ^∗∗^ = p ≤ 0.01, ^∗∗∗^ = p ≤ 0.001). See also Table S2 and S3 for the raw data and results of the statistical tests.

Based on the mean intensity and standard deviation of a control set of wings, we aimed for a sample size of at least 6 wings per genotype. This would allow detection of differences of 20% in the means, in a pairwise comparison (calculated using G Power).

Each experiment was performed on multiple wings from different pupae, which represent biological replicates (n, number of wings). For each wing, 8 ROIs were selected for FRAP analysis, and these were treated as technical replicates and were averaged per wing to produce a y[max] and a stable amount per wing. Data were excluded if the ROI recovery curve failed the ‘replicates test for lack of fit’ in Prism, or if the wing moved out of focus during the course of imaging. In total 22 wings were discarded across all the genotypes.

#### Statistics

The overall intensities and stable and unstable amounts for multiple genotypes were compared using one-way ANOVA, to take into account the sample variation across the genotypes analyzed and to avoid multiple t test analysis. Post hoc tests were used to compare individual samples: Dunnett’s multiple comparison test was used to compare the control to the rest of the genotypes in the experiment; Tukey-Kramer’s multiple comparison test to compare all genotypes within an experiment; and Holm-Šídák’s multiple comparison test was sometimes used to compare genotypes pairwise. Where a post hoc test was used this is described in the Figure legends, and multiplicity adjusted p values calculated in Prism are reported on the graph as asterisks (^∗^ = p ≤ 0.5, ^∗∗^ = p ≤ 0.01, ^∗∗∗^ = p ≤ 0.001).

#### Polarity measurement

A MATLAB script was used to determine the angle of maximum asymmetry for each cell (see (Strutt et al., 2016) for MATLAB scripts). The mean vector polarity was then averaged for all cells in the image to give a mean vector polarity per wing (asymmetry ratio on plots). Results were averaged across wings and compared using ANOVA with Tukey’s multiple comparison test.

#### Quantification of Pk propagation

Pk cytoplasmic levels were selected in immunolabelled confocal images inside and outside acute knockdown clone tissue using ImageJ. A circular shape was drawn inside each cell (without touching the membrane) and the same shape used throughout all measurements. The mean intensity was determined for each cell, and averaged to give a mean intensity per wing. Results were averaged across wings and ANOVA with Dunnett’s multiple comparison test was used to compare cells away from the clone boundary to cells just inside the clone.

#### Quantification of Pk membrane levels

Pk membrane levels were selected in immunolabelled confocal images inside and outside acute knockdown tissue using ImageJ. A line was drawn on each vertical cell junction and the same shape used throughout all measurements. A minimum of 5 cell membrane measurements were taken per wing and averaged to give a mean intensity per wing. Results were averaged across wings and compared using a paired parametric t test or a paired one-way ANOVA.

## Supplemental Information

Document S1. Figures S1–S4 and Tables S1–S3

Document S2. Article plus Supplemental Information
