# Supplementary material for: Rapid Disruption of Dishevelled Activity Uncovers an Intercellular Role in Maintenance of Prickle in Core Planar Polarity Protein Complexes
Source: Cell Rep. 2018 Nov 6;25(6):1415–1424.e6. doi: 10.1016/j.celrep.2018.10.039 (PMC6231328; doi:10.1016/j.celrep.2018.10.039)
Supplement: Document S1. Figures S1–S4 and Tables S1–S3 [file mmc1.pdf]

Cell Reports, Volume *25*

Supplemental Information

Rapid Disruption of Dishevelled Activity Uncovers an Intercellular Role in Maintenance of Prickle in Core Planar Polarity Protein Complexes

Margarida Ressurreição, Samantha Warrington, and David Strutt

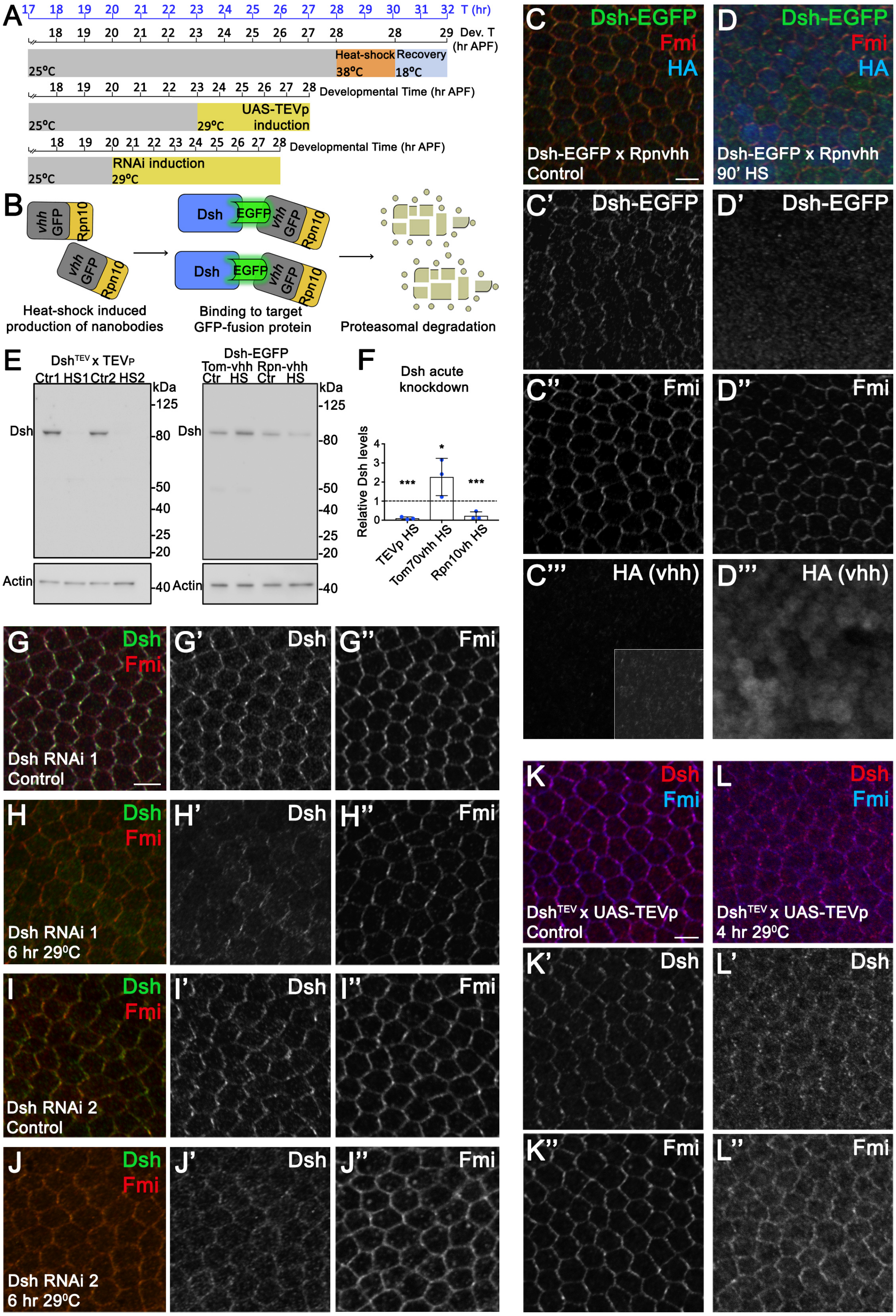

A
T (hr)
Dev. T (hr APF)
Heat-shock 38°C
Recovery 18°C
25°C
Developmental Time (hr APF)
UAS-TEVp induction
29°C
RNAi induction
B
Heat-shock induced production of nanobodies
Binding to target GFP-fusion protein
Proteasomal degradation
Dsh
EGFP
vhh GFP
Rpn10
E
Dsh^TEV x TEVp
Ctr1 HS1 Ctr2 HS2
Dsh-EGFP
Tom-vhh Rpn-vhh
Ctr HS Ctr HS
kDa
Dsh
Actin
F
Dsh acute knockdown
Relative Dsh levels
TEVp HS
Tom70vhh HS
Rpn10vh HS
C
Dsh-EGFP
Fmi
HA
Dsh-EGFP x Rpnvhh Control
D
Dsh-EGFP x Rpnvhh 90' HS
HA (vhh)
G
Dsh RNAi 1 Control
H
Dsh RNAi 1 6 hr 29°C
I
Dsh RNAi 2 Control
J
Dsh RNAi 2 6 hr 29°C
K
Dsh^TEV x UAS-TEVp Control
L
Dsh^TEV x UAS-TEVp 4 hr 29°C

Figure S1. Disruption of Dishevelled activity in the *Drosophila* pupal wing. Related to Figure 1.

(A) Diagram representing the temperature regimes employed to knockdown Dishevelled acutely, via RNAi or UAS-TEVp in the 28 hr APF wing epithelium. 'Dev. T' is the developmental time equivalent at 25°C.

(B) Dsh-EGFP disruption based on targeting with anti-GFP nanobodies fused to the proteosomal subunit Rpn10. Upon Rpn10-HA-vhhGFP production via heat-shock, Dsh-EGFP is targeted for degradation.

(C,D) 28 hr APF wing epithelium heterozygous for *dsh-EGFP* and *hs-Rpn10-HA-vhhGFP* in a dsh^{V26} null background. (C) No heat-shock, showing native GFP (green, C'), endogenous Fmi expression (red, C'') and HA labelling (blue, C'''). Inset is a region in C''' where intensity was digitally increased to show there is negligible HA expression. (D) Disruption of Dsh-EGFP by Rpn10-HA-vhhGFP expression after induction by a 90 min heat-shock at 38°C. (D') is GFP, (D'') endogenous Fmi expression and (D''') HA labelling showing strong cytoplasmic signal corresponding to expression of Rpn10-HA-vhhGFP. Scale bars = 5µm and are the same hereafter.

(E) Western blots showing levels of either Dsh^{TEV} or Dsh-GFP proteins before and after acute knockdown via heat-shock (38°C) when in the presence of *hs-TEVp, hs-Tom70-HA-vhhGFP* or *hs-Rpn10-HA-vhhGFP,* in a dsh^{V26} background. The left blot shows two independent experiments before and after Dsh^{TEV} cleavage by TEVp. Lanes labelled Ctr are Dsh^{TEV} in the presence of *hs-TEVp* without heat-shock induction, showing only one band at the expected molecular weight for Dsh. Lanes labelled HS are of Dsh^{TEV} in the presence of *hs-TEVp* after a 2 hr heat-shock and 1 hr recovery at 18°C, showing negligible detection of Dsh. The blot on the right shows Dsh-GFP before and after induction of Tom70-HA-vhhGFP or Rpn10-HA-vhhGFP. Lanes labelled Ctr are of Dsh-EGFP in the presence of the two differently anchored anti-GFP nanobodies, without heat-shock induction. Lanes labelled HS are of Dsh-EGFP in the presence of Tom70-HA-vhhGFP or Rpn10-HA-vhhGFP after a 2 hr or a 90 min heat-shock at 38°C, respectively. Note that Dsh-EGFP is still present after the production of Tom70-HA-vhhGFP as this presumably relocates Dsh-EGFP from the membrane to the mitochondrial network. Meanwhile Dsh-EGFP is visibly reduced after the production of Rpn10-HA-vhhGFP as this should relocate Dsh-EGFP to proteasomes.

(F) Quantification of the relative change in either Dsh^{TEV} or Dsh-EGFP protein levels when disrupted using TEVp, Tom70-HA-vhhGFP or Rpn10-HA-vhhGFP. Each data point is an average of the replicates of three independent experiments, normalised to actin and then to the control. Error bars are standard deviation. Unpaired t-tests were used to compare experimental and control samples, ***$p \leq 0.001$.

(G-J) 28 hr APF wing epithelia from male flies carrying two different RNAi lines against *dsh* transcripts and *Act-GAL4, tub-GAL80^ts^*. (G,I) When maintained at 25°C for 28 hr, wing tissue shows normal asymmetric planar polarised localisation of endogenous Dsh (G', I') and Fmi (G'', I''). (H) Upon a temperature shift regime to 29°C for 6 hr to induce the production of $dsh^{NIG18361R-2}$ RNAi, Dsh labelling is still evident, albeit patchy (H') and Fmi labelling is still present and asymmetric (H''). (J) Upon a temperature shift to 29°C for 6 hr to induce dsh^{WIZ} RNAi expression, Dsh (J') and Fmi (J'') labelling is still detectable at the cell membrane.

(K-L) 28 hr APF wing epithelia heterozygous for Dsh^{TEV}, *UAS-TEVp* and *Act-GAL4,* $tub\text{-}GAL80^{ts}$*,* in a dsh^{V26} null background. (K) Pupae maintained at 25°C for 28 hr show a normal asymmetric localisation of Dsh^{TEV} (K') and Fmi (K'') suggesting that TEVp production is negligible under these conditions. (L-L'') Upon a temperature shift to 29°C for 4 hr, Dsh (L') and Fmi (L'') labelling is still asymmetrically present at the cell membrane, however localisation is weaker and more diffuse.

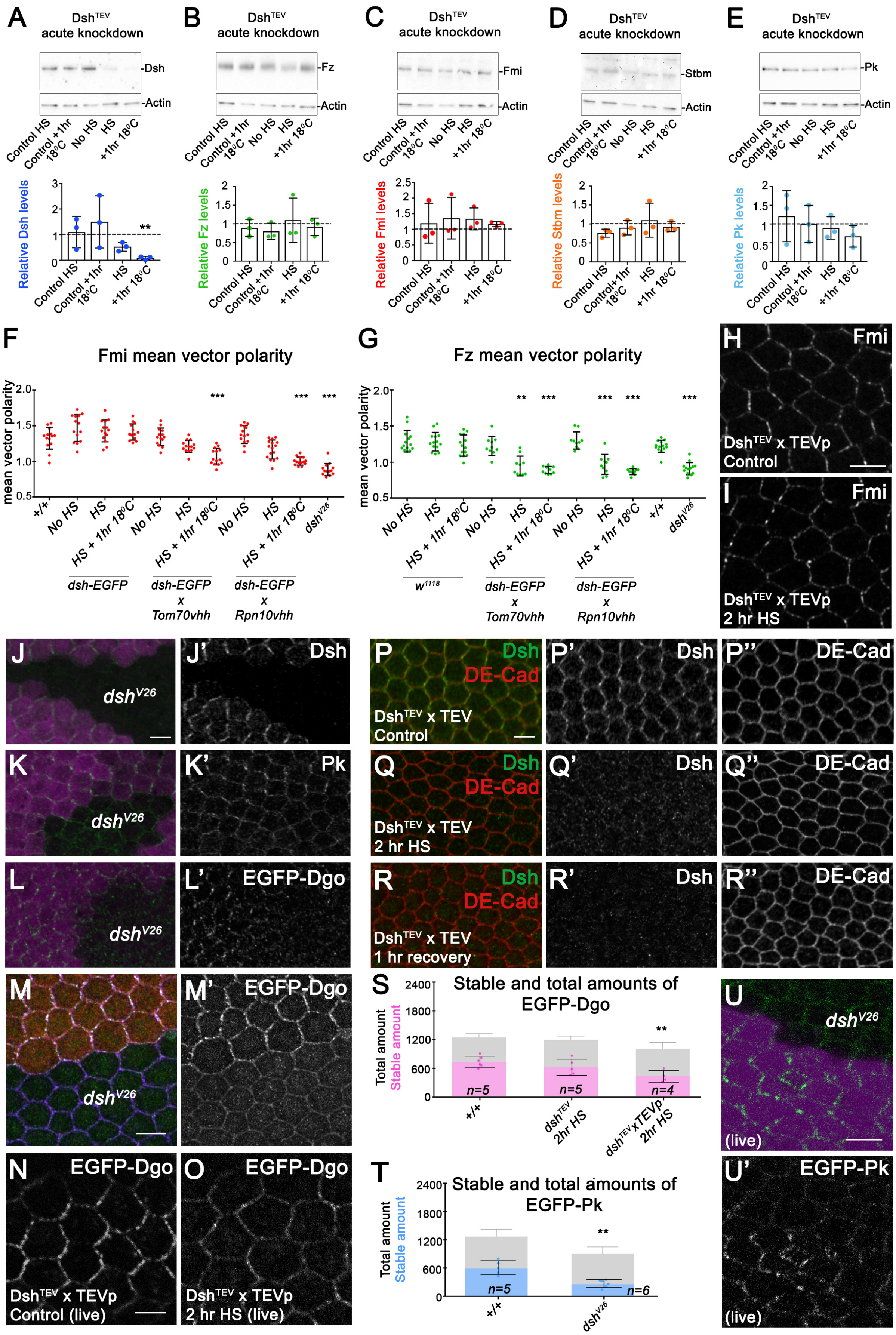
A
Dsh^TEV acute knockdown
-Dsh
-Actin
Control HS
Control +1hr 18°C
No HS
HS
+1hr 18°C
Relative Dsh levels
**
B
Dsh^TEV acute knockdown
-Fz
-Actin
Relative Fz levels
C
Dsh^TEV acute knockdown
-Fmi
-Actin
Relative Fmi levels
D
Dsh^TEV acute knockdown
-Stbm
-Actin
Relative Stbm levels
E
Dsh^TEV acute knockdown
-Pk
-Actin
Relative Pk levels
F
Fmi mean vector polarity
mean vector polarity
***
+/+
No HS
HS
HS + 1hr 18°C
dsh^V26
dsh-EGFP
dsh-EGFP x Tom70vhh
dsh-EGFP x Rpn10vhh
G
Fz mean vector polarity
mean vector polarity
**
***
w^1118
dsh-EGFP
dsh-EGFP x Tom70vhh
dsh-EGFP x Rpn10vhh
H
Fmi
Dsh^TEV x TEVp
Control
I
Fmi
Dsh^TEV x TEVp
2 hr HS
J
dsh^V26
J'
Dsh
K
dsh^V26
K'
Pk
L
dsh^V26
L'
EGFP-Dgo
M
dsh^V26
M'
EGFP-Dgo
N
EGFP-Dgo
Dsh^TEV x TEVp
Control (live)
O
EGFP-Dgo
Dsh^TEV x TEVp
2 hr HS (live)
P
Dsh
DE-Cad
Dsh^TEV x TEV
Control
P'
Dsh
P''
DE-Cad
Q
Dsh
DE-Cad
Dsh^TEV x TEV
2 hr HS
Q'
Dsh
Q''
DE-Cad
R
Dsh
DE-Cad
Dsh^TEV x TEV
1 hr recovery
R'
Dsh
R''
DE-Cad
S
Stable and total amounts of EGFP-Dgo
Total amount
Stable amount
n=5
n=5
n=4
**
+/+
dsh^TEV 2hr HS
dsh^TEV x TEVp 2hr HS
T
Stable and total amounts of EGFP-Pk
Total amount
Stable amount
n=5
n=6
**
+/+
dsh^V26
U
dsh^V26
(live)
U'
EGFP-Pk
(live)

Figure S2. Dishevelled maintains stability of core planar polarity proteins. Related to Figure 2.

(A-E) Western blots from 28 hr APF pupal wing extracts, showing protein levels of either Dsh^{TEV} (A), Fz (B), Fmi (C), Stbm (D) or Pk (E) before and after Dsh^{TEV} acute knockdown. Lanes labelled Control HS and Control + 1hr 18°C are immediately after, and one hour after heat-shock induction of *hs-TEVp* with wild-type Dsh as a control for the temperature regimes. Lanes labelled No HS, HS and + 1hr 18°C, are from flies expressing Dsh^{TEV} in a *dsh*^{V26} and *hs-TEVp* background before, after a 2 hr heat-shock, and 1 hr at 18°C after a 2 hr heat-shock regime. Below each blot is the quantification for each detected protein from three independent experiments normalised to actin and then to the No HS condition. Error bars are standard deviation. ANOVA with Dunnett's multiple comparisons test were used to compare experimental to no heat-shock control samples, $**p\leq 0.01$.

(F,G) Fmi (F) and Fz (G) mean vector polarity values after disruption of Dsh-EGFP using vhhGFP. Error bars are standard deviation. ANOVA with Tukey-Kramer's multiple comparison test was used to compare all samples. Shown are comparisons of each genotype to no heat-shock conditions and +/+ to *dsh*^{V26} clone tissue. $***p\leq0.001$, $**p\leq0.01$.

(H,I) AiryScan super-resolution microscopy of 28 hr APF wings, immunolabelled for Fmi, before (H) and after (I) Dsh^{TEV} cleavage.

(J-L) Immunolabelling of 28 hr APF wings containing clones of *dsh*^{V26} null mutant tissue (loss of RFP, magenta), labelled for Dsh (green, J), Pk (green, K) and EGFP-Dgo (green, L). Scale bars = 5μm and are the same hereafter.

(M) AiryScan super-resolution image of EGFP-Dgo in wild-type and *dsh*^{V26} null mutant tissue. While EGFP-Dgo is hard to visualise at cell membranes of *dsh*^{V26} mutant tissue by confocal microscopy (L'), an EGFP-Dgo signal at cell junctions is weakly detected using AiryScan microscopy (M').

(N,O) Live imaging of 28 hr APF wings expressing EGFP-Dgo before (N) and after (O) cleavage of Dsh^{TEV}.

(P-R) Dsh^{TEV} (green) and E-cadherin (red) immunolabelling of 28 hr APF wings before cleavage of Dsh^{TEV} (P), immediately after (Q) and 1 hr after (R), showing the sustained absence of Dsh labelling after heat-shock induction.

(S) FRAP analysis of 28 hr APF pupal wings expressing EGFP-Dgo, showing stable and total amounts of EGFP-Dgo before and after Dsh^{TEV} cleavage. Error bars are standard deviation, n = number of wings. ANOVA with Tukey's multiple comparison test was used to compare all genotypes, p=0.0088 comparing +/+ to Dsh^{TEV}xTEVp. Also see Table S2 and Table S3.

(T) FRAP analysis of 28 hr APF pupal wings expressing EGFP-Pk in wild-type Dsh and *dsh* null tissue. Error bars are standard deviation, n = number of wings. Genotypes were compared using an unpaired t-test, p=0.0011. Also see Table S2 and Table S3.

(U) Live imaging of 28 hr APF wings containing clones of *dsh*^{V26} null mutant tissue (loss of RFP, magenta), expressing EGFP-Pk (green, U').

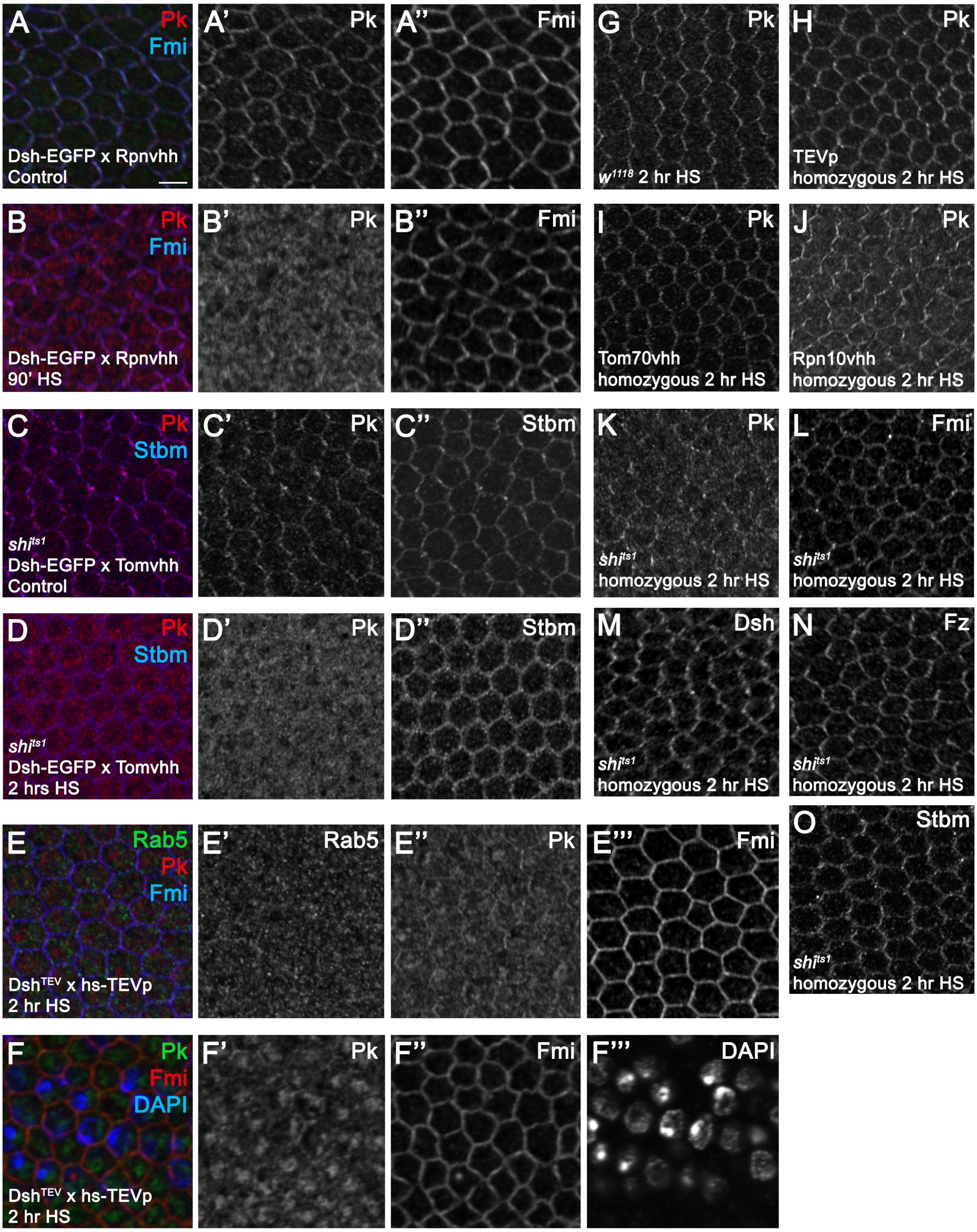

A
Pk
Fmi
Dsh-EGFP x Rpnvhh
Control
A'
Pk
A''
Fmi
G
Pk
w1118 2 hr HS
H
Pk
TEVp
homozygous 2 hr HS
B
Pk
Fmi
Dsh-EGFP x Rpnvhh
90' HS
B'
Pk
B''
Fmi
I
Pk
Tom70vhh
homozygous 2 hr HS
J
Pk
Rpn10vhh
homozygous 2 hr HS
C
Pk
Stbm
shits1
Dsh-EGFP x Tomvhh
Control
C'
Pk
C''
Stbm
K
Pk
shits1
homozygous 2 hr HS
L
Fmi
shits1
homozygous 2 hr HS
D
Pk
Stbm
shits1
Dsh-EGFP x Tomvhh
2 hrs HS
D'
Pk
D''
Stbm
M
Dsh
shits1
homozygous 2 hr HS
N
Fz
shits1
homozygous 2 hr HS
E
Rab5
Pk
Fmi
DshTEV x hs-TEVp
2 hr HS
E'
Rab5
E''
Pk
E'''
Fmi
O
Stbm
shits1
homozygous 2 hr HS
F
Pk
Fmi
DAPI
DshTEV x hs-TEVp
2 hr HS
F'
Pk
F''
Fmi
F'''
DAPI

Figure S3. Removal of Dsh from core planar polarity protein complexes results in the translocation of Pk from the membrane to the cytoplasm. Related to Figure 3.

(A,B) 28 hr APF pupal wings before and after sequestration of Dsh-EGFP by Rpn10-HA-vhhGFP, showing Pk (A' and B') and Fmi (A'' and B''). Scale bar = 5µm.

(C,D) Immunolabelling of 28 hr APF wings hemizygous for shi^{ts1} before (C) and after (D) sequestration of Dsh-EGFP by Tom70-HA-vhhGFP. (C) Pk (C') and Stbm (C'') localise asymmetrically. (D) Pk (D') is cytoplasmic and Stbm (D'') is junctional.

(E) 28 hr APF pupal wings after cleavage of Dsh^{TEV}, showing negligible colocalisation between Rab5 (E') and cytoplasmic Pk (E''), Fmi staining defines the cell junctions (E''').

(F) 28 hr APF pupal wings after cleavage of Dsh^{TEV}, showing DAPI (F''') and cytoplasmic Pk (F'), Fmi staining defines the cell junctions (F'').

(G-J) Immunolabelling of 28 hr APF wings from wild-type (G), *hs-TEVp* (H), *hs-Tom70-HA-vhhGFP* (I) or *hs-Rpn10-HA-vhhGFP* (J) to assess non-specific effects on Pk localisation after a 2 hr heat-shock.

(K-O) Immunolabelling of 28 hr APF wings from shi^{ts1} flies, to assess non-specific effects on Pk (K), Fmi (L), Dsh (M), Fz (N) and Stbm (O) localisation after a 2 hr heat-shock.

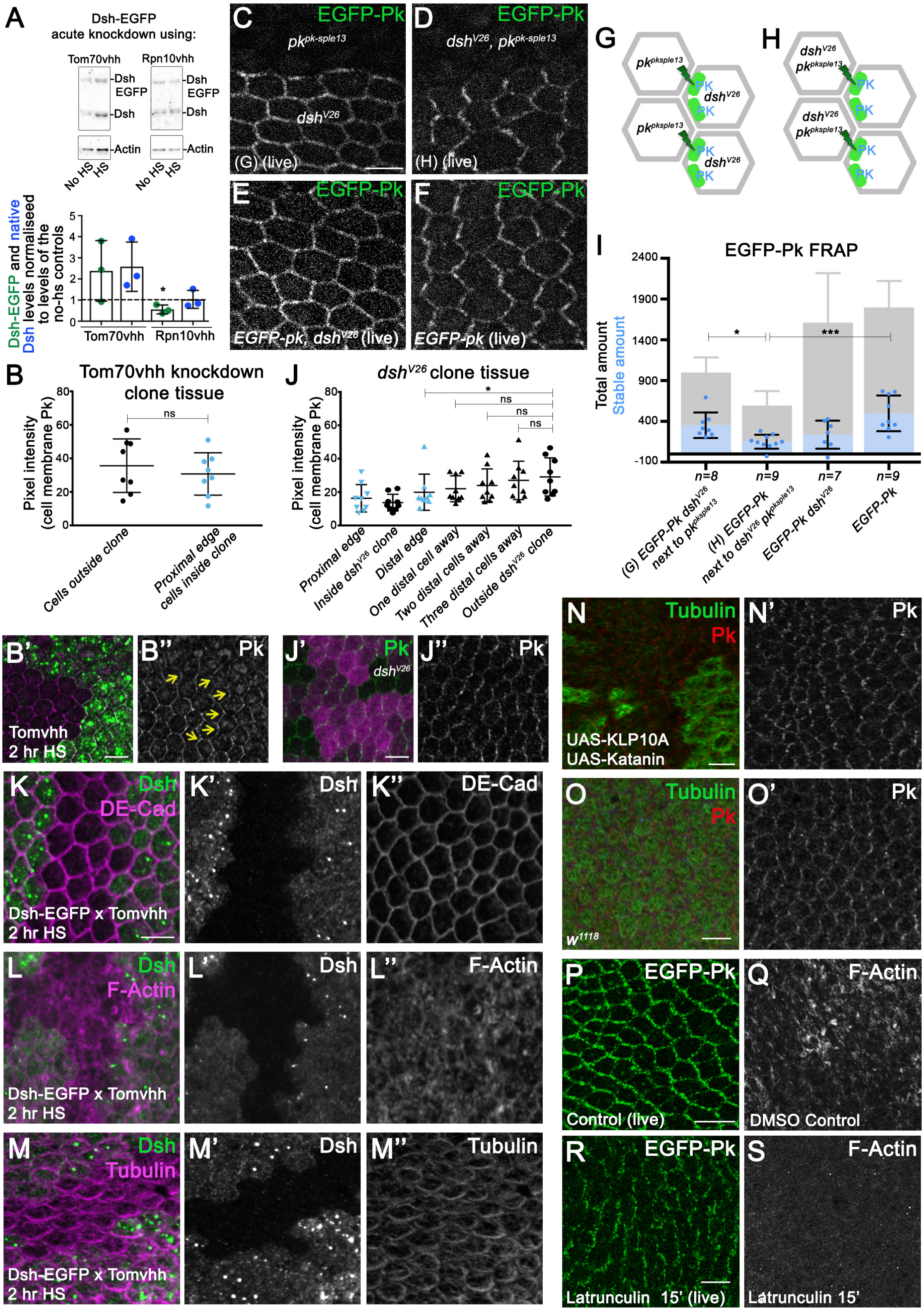

A
Dsh-EGFP acute knockdown using:
Tom70vhh
Rpn10vhh
Dsh EGFP
Dsh
Actin
No HS
HS
Dsh-EGFP and native Dsh levels normalised to levels of the no-hs controls
Tom70vhh
Rpn10vhh
B
Tom70vhh knockdown clone tissue
Pixel intensity (cell membrane Pk)
Cells outside clone
Proximal edge cells inside clone
ns
C
EGFP-Pk
pkpk-sple13
dshV26
(G) (live)
D
EGFP-Pk
dshV26, pkpk-sple13
(H) (live)
E
EGFP-Pk
EGFP-pk, dshV26 (live)
F
EGFP-Pk
EGFP-pk (live)
G
H
I
EGFP-Pk FRAP
Total amount
Stable amount
n=8
n=9
n=7
n=9
(G) EGFP-Pk dshV26 next to pkpksple13
(H) EGFP-Pk next to dshV26 pkpksple13
EGFP-Pk dshV26
EGFP-Pk
J
dshV26 clone tissue
Proximal edge
Inside dshV26 clone
Distal edge
One distal cell away
Two distal cells away
Three distal cells away
Outside dshV26 clone
B'
Tomvhh 2 hr HS
B''
Pk
J'
Pk
dshV26
J''
Pk
K
Dsh
DE-Cad
Dsh-EGFP x Tomvhh 2 hr HS
K'
Dsh
K''
DE-Cad
L
Dsh
F-Actin
Dsh-EGFP x Tomvhh 2 hr HS
L'
Dsh
L''
F-Actin
M
Dsh
Tubulin
Dsh-EGFP x Tomvhh 2 hr HS
M'
Dsh
M''
Tubulin
N
Tubulin
Pk
UAS-KLP10A
UAS-Katanin
N'
Pk
O
Tubulin
Pk
w1118
O'
Pk
P
EGFP-Pk
Control (live)
Q
F-Actin
DMSO Control
R
EGFP-Pk
Latrunculin 15' (live)
S
F-Actin
Latrunculin 15'

Figure S4. Dsh acts on Pk cell non-autonomously and independently of the cytoskeleton. Related to Figure 4.

(A) Western blots from 28 hr APF pupal wing extracts, showing levels of Dsh-GFP and native Dsh proteins before and after acute knockdown using *hs-Tom70-HA-vhhGFP* or *hs-Rpn10-HA-vhhGFP,* in a heterozygous dsh^{V26} background. Lanes labelled No HS show Dsh-EGFP and native Dsh in a background containing the anti-GFP nanobody transgenes, without heat-shock induction. Lanes labelled HS show Dsh-EGFP and native Dsh in the presence of Tom70-HA-vhhGFP or Rpn10-HA-vhhGFP after a 2 hr or a 90 min heat-shock at 38°C, respectively. Quantitation of Dsh-EGFP and Dsh levels, from three independent experiments is shown below, normalised to actin and then to the levels in No HS. Note that Dsh-EGFP is still present (and indeed shows increased levels) after the production of Tom70-HA-vhhGFP; while it is significantly reduced in the presence of Rpn10-HA-vhhGFP. Meanwhile native Dsh levels also increase after production of Tom70-HA-vhhGFP (presumably due to co-sequestration to mitochondria by Dsh-EGFP) while they do not decrease after the production of Rpn10-HA-vhhGFP. *p=0.05.

(B) Quantification of proximodistal membrane localised Pk in cells outside and at the proximal edge of cells at the proximal edge of Dsh-EGFP clones in Tom70-vhh knockdown tissue, and representative images (B',B'', yellow arrows indicate Pk at proximal edge of cells at proximal edge of clones). Error bars are standard deviation, each data point is one wing. A paired t-test was used to compare measurements between the outside and the proximal edge of the clone.

(C-I) FRAP analysis of EGFP-Pk on boundaries of dsh^{V26} null clones that border cells expressing wild-type levels of Dsh. (C-H) Representative images and diagrams, showing EGFP-Pk in a wild-type (F) or dsh^{V26} (E) background, or clones where EGFP-Pk is expressed in dsh^{V26} cells next to $pk^{pk\text{-}sple13}$ mutant cells (C,G) or in wild-type cells next to double-mutant dsh^{V26} and $pk^{pk\text{-}sple13}$ cells (D,H). Images show EGFP-Pk in white. Cells lacking EGFP-Pk were made mutant for $pk^{pk\text{-}sple13}$, to avoid possible confounding effects of Dsh on these unlabelled populations of Pk. (I) FRAP of EGFP-Pk (left to right): First column – boundaries between dsh^{V26} *EGFP-Pk* cells and $pk^{pk\text{-}sple13}$ cells; second column – boundaries between *EGFP-Pk* cells and dsh^{V26} $pk^{pk\text{-}sple13}$ cells; third and fourth columns – EGFP-Pk on vertical orientated junctions in a dsh^{V26} background or a wild-type background. The stable amount of EGFP-Pk in wild-type tissue (blue bar in fourth column) is significantly higher compared to that of EGFP-Pk in wild-type cells neighbouring cells that lack Dsh (blue bar in second column) ***$p\leq0.001$. While loss of Dsh in the same cell as EGFP-Pk (blue bar in first column) reduces overall EGFP-Pk levels (reduced height of grey bar in first column), the stable amount of EGFP-Pk is not significantly different to that of EGFP-Pk in wild-type cells (fourth column) $p=0.2094$. In addition, the stable amount of EGFP-Pk in a wild-type cell next to dsh^{V26} minus cells (second column) is decreased compared to EGFP-Pk in dsh^{V26} cells next to wild-type cells (first column) *$p\leq0.05$. Error bars are standard deviation, n = number of wings. ANOVA and a Holm-Šídák multiple comparison test was used to compare columns 1 and 2, 1 and 3, and 2 and 4. Also see Table S2.

(J) Quantification of membrane localised Pk at the proximal edge of cells at the proximal edge of dsh^{V26} clone tissue, inside clone tissue, at the distal cell edge at the distal edge of clone tissue, and at cell edges one to three distal cells away from, clone tissue; with a representative image (J'). Error bars are standard deviation, each data point is one wing. One-way ANOVA and a Tukey's multiple comparison test was used to compare measurements from outside dsh^{V26} tissue with all other measurements, *$p\leq0.05$.

(K-M) Images of *Drosophila* wing epithelia at 28 hr APF. Clone tissue is two copies of Dsh-EGFP or no Dsh-EGFP and heterozygous tissue is one copy of Dsh-EGFP, all in a background heterozygous for *hs-Tom70-HA-vhhGFP* and dsh^{V26}. After a 2 hr heat-shock, Dsh-EGFP is sequestered away from cell junctions (K', L' and M'). E-cad (K''), F-actin (L'') and β-Tubulin (M'') show no change in distribution between knockdown and non-targeted tissue.

(N) β-Tubulin and Pk localisation in tissue heterozygous for *Act-GAL4, tub-GAL80*^{ts}, and *UAS-KLP10A*, *UAS-Katanin-60*, maintained at 25°C until 28 hr APF. Under these conditions there is leaky expression of the transgenes, resulting in patchy disruption of microtubules in a stochastic manner. Pk remains at the membrane even in the absence of a visible microtubule network.

(O) β-Tubulin (green) and Pk (red) localization in w^{1118} wing epithelial cells at 25°C (28 hr APF), showing normal distributions.

(P,Q) 6 hr APF prepupal wings incubated for 15 min in 0.1% DMSO. (P) Live imaging of EGFP-Pk shows a normal distribution for that developmental stage. (Q) F-actin distribution detected by Phalloidin-Alexa568 in fixed wings.

(R,S) 6 hr APF prepupal wings incubated for 15 min in 2μM of Latrunculin A diluted in DMSO. (R) Live imaging of EGFP-Pk shows a membranous distribution. (S) F-actin detected by Phalloidin-Alexa568 in fixed wings. Note substantial loss of F-actin network.

Genotype	Treatment	n = number of wings analysed	Mean Vector Polarity	s.d.	*p* values Comparing to No HS condition	*p* values Comparing to w^{1118}
**Fmi immunolabelling**						
w^{1118}	25ºC (No HS)	14	1.363	0.137	n/a	n/a
w^{1118}	2 hr (38ºC)	14	1.321	0.099	0.951	n/a
w^{1118}	2 hr (38ºC) + 1 hr (18ºC)	14	1.375	0.224	0.892	n/a
$dsh^{V26}/Y; dsh^{TEV}$	25ºC (No HS)	9	1.400	0.137	n/a	0.875
$dsh^{V26}/Y; dsh^{TEV}$	2 hr (38ºC)	10	1.351	0.245	0.902	>0.999
$dsh^{V26}/Y; dsh^{TEV}$	2 hr (37ºC) + 1 hr (18ºC)	7	1.404	0.121	0.659	0.900
hs-TEVp	25ºC (No HS)	7	1.514	0.067	n/a	1.000
hs-TEVp	2 hr (38ºC)	10	1.581	0.169	0.998	0.315
hs-TEVp	2 hr (38ºC) + 1 hr (18ºC)	7	1.559	0.131	0.997	0.850
$dsh^{V26}/Y; dsh^{TEV}/$ *hs-TEVp*	25ºC (No HS)	21	1.365	0.168	n/a	>0.999
$dsh^{V26}/Y; dsh^{TEV}/$ *hs-TEVp*	2 hr (38ºC)	21	1.266	0.109	0.055	0.829
$dsh^{V26}/Y; dsh^{TEV}/$ *hs-TEVp*	2 hr (38ºC) + 1 hr (18ºC)	21	1.085	0.121	0.000	0.043
$dsh^{V26}/Y;$ *dsh-EGFP*	25ºC (No HS)	10	1.470	0.153	n/a	0.805
$dsh^{V26}/Y;$ *dsh-EGFP*	2 hr (38ºC)	10	1.341	0.206	>0.999	0.875
$dsh^{V26}/Y;$ *dsh-EGFP*	2 hr (38ºC) + 1 hr (18ºC)	10	1.435	0.188	>0.999	0.875
hs-Tom70-HA-vhhGFP	25ºC (No HS)	8	1.314	0.155	n/a	>0.999
hs-Tom70-HA-vhhGFP	2 hr (38ºC)	9	1.330	0.141	>0.999	>0.999
hs-Tom70-HA-vhhGFP	2 hr (38ºC) + 1 hr (18ºC)	9	1.269	0.130	0.999	0.893
$dsh^{V26}/Y;$ *dsh-EGFP/ hs-Tom70-HA-vhhGFP*	25ºC (No HS)	14	1.345	0.123	n/a	0.385
$dsh^{V26}/Y;$ *dsh-EGFP/ hs-Tom70-HA-vhhGFP*	2 hr (38ºC)	14	1.211	0.084	0.163	0.132
$dsh^{V26}/Y;$ *dsh-EGFP/ hs-Tom70-HA-vhhGFP*	2 hr (38ºC) + 1 hr (18ºC)	14	1.067	0.115	<0.0001	<0.0001
hs-Rpn10-HA-vhhGFP	25ºC (No HS)	7	1.190	0.178	n/a	0.967
hs-Rpn10-HA-vhhGFP	90 min (38ºC)	15	1.200	0.229	0.992	0.999
hs-Rpn10-HA-vhhGFP	90 min (38ºC) + 1 hr (18ºC)	8	1.107	0.132	0.929	0.967
$dsh^{V26}/Y;$ *dsh-EGFP/ hs-Rpn10-HA-vhhGFP*	25ºC (No HS)	14	1.378	0.125	n/a	0.989
$dsh^{V26}/Y;$ *dsh-EGFP/ hs-Rpn10-HA-vhhGFP*	2 hr (38ºC)	16	1.197	0.233	0.053	0.053
$dsh^{V26}/Y;$ *dsh-EGFP/ hs-Rpn10-HAvhhGFP*	2 hr (38ºC) + 1 hr (18ºC)	14	1.004	0.057		<0.0001

Table S1. Related to Figure 2 and S2.

Mean vector polarity measurements of Fmi immunolabelling in 28 hr APF pupal wing tissue for all genotypes before and after heat-shock. Vector polarity was calculated for a group of cells within a field of view in an individual wing. ‘n’ indicates the numbers of wings examined. Results were averaged across wings and standard deviations (s.d.) calculated. ANOVA with Tukey’s multiple comparison test was used to compare between genotypes. In column 6 (‘No hs’) are the results of comparing the heat-shocked samples to the non heat-shocked samples within a genotype. In column 7 (‘w^{1118}’) are the results of comparing each heat-shock regime to control w^{1118} wings treated to the same heat-shock regime.

Fz-EGFP before and after cleavage of Dsh^{TEV}

Genotype	n	Total fluorescent mean intensity	Total mean intensity confidence intervals	Stable amount	Stable amount 95% confidence intervals	y[max]	y[max] with 95% confidence intervals	t[0.5] Half-life	Half-life with 95% confidence Intervals	Figure
w^{1118}; fz-EGFP/+	6	2583.00	2363 to 2803	1588.00	1377 to 1799	0.36	0.335 to 0.385	48.07	38.71 to 63.39	Fig.2N
dsh^{V26}/Y; P[acman]-dsh^{TEV}/+; fz-EGFP/+ (2 hr HS)	6	2539.00	2066 to 3012	1393.00	1173 to 1612	0.46	0.371 to 0.551	40.22	0.304 to 0.3404	Fig.2N
dsh^{V26}/Y; P[acman]-dsh^{TEV}/+; fz-EGFP/+ (2 hr HS + 18°C)	6	2208.00	1921 to 2388	1444.00	1191 to 1697	0.36	0.294 to 0.422	60.58	0.367 to 0.420	Fig.2N
dsh^{V26}/Y; P[acman]-dsh^{TEV}/P[CaSpeR]-hs-TEVp; fz-EGFP/+ (No HS)	7	2216.00	1852 to 2580	1365.00	1190 to 1541	0.40	0.300 to 0.490	66.36	55.93 to 81.56	Fig.2N
dsh^{V26}/Y; P[acman]-dsh^{TEV}/P[CaSpeR]-hs-TEVp; fz-EGFP/+ (2 hr HS)	7	2472.00	1936 to 3008	1054.00	889.5 to 1218	0.58	0.545 to 0.613	66.84	55.12 to 84.92	Fig.2N
dsh^{V26}/Y; P[acman]-dsh^{TEV}/P[CaSpeR]-hs-TEVp; fz-EGFP/+ (2 hr HS + 18°C)	6	2386.50	647 to 2322	845.20	671.1 to 1019	0.65	0.602 to 0.703	36.24	29.77 to 46.31	Fig.2N
dsh^{V26} FRT19A/ ubi-mRFP-nls FRT19A; Ubx-FLP/+; fz-EGFP/+	6	2403.60	373.6 to 2144	833.90	534.9 to 1133	0.66	0.605 to 0.713	26.94	20.75 to 38.38	Fig.2N

Fmi-EGFP before and after cleavage of Dsh^{TEV}

Genotype	n	Total fluorescent mean intensity	Total mean intensity confidence intervals	Stable amount	Stable amount 95% confidence intervals	y[max]	y[max] with 95% confidence intervals	t[0.5] Half-life	Half-life with 95% confidence Intervals	Figure
w^{1118}; fmi-EGFP/+	8	2454.16	2069 to 2839	1830.24	1569 to 2092	0.27	0.184 to 0.352	12.52	8.568 to 23.25	Fig.2P
dsh^{V26}/Y; P[acman]-dsh^{TEV}/fmi-EGFP (HS)	8	2280.58	1955 to 2446	1754.36	1620 to 1789	0.24	0.222 to 0.268	47.64	60.62 to 79.33	Fig.2P
dsh^{V26}/Y; P[acman]-dsh^{TEV}/fmi-EGFP (2 hr HS + 18°C)	8	2096.58	1780 to 2171	1670.26	1541 to 1800	0.22	0.207 to 0.230	86.09	109.9 to 142.9	Fig.2P
dsh^{V26}/Y; P[acman]-dsh^{TEV}/fmi-EGFP; P[CaSpeR]-hs-TEVp/+ (No HS)	8	2399.95	2330 to 2470	1835.06	1727 to 1943	0.25	0.211 to 0.288	48.31	60.62 to 81.97	Fig.2P
dsh^{V26}/Y; P[acman]-dsh^{TEV}/fmi-EGFP; P[CaSpeR]-hs-TEVp/+ (2 hr HS)	8	2096.41	1457 to 2179	1484.57	1305 to 1664	0.30	0.296 to 0.314	31.04	38.32 to 53.85	Fig.2P
dsh^{V26}/Y; P[acman]-dsh^{TEV}/fmi-EGFP; P[CaSpeR]-hs-TEVp/+ (2 hr HS + 18°C)	8	2064.16	1834 to 2294	1336.90	1158 to 1515	0.36	0.342 to 0.387	56.99	48.69 to 68.71	Fig.2P
dsh^{V26} FRT19A/ ubi-mRFP-nls FRT19A; Ubx-FLP/fmi-EGFP	6	1644.59	1083 to 1884	949.51	686.9 to 1212	0.43	0.429 to 0.4379	34.78	25.31 to 55.55	Fig.2P

Stbm-EGFP before and after cleavage of Dsh^{TEV}

Genotype	n	Total fluorescent mean intensity	Total mean intensity confidence intervals	Stable amount	Stable amount 95% confidence intervals	y[max]	y[max] with 95% confidence intervals	t[0.5] Half-life	Half-life with 95% confidence Intervals	Figure
w^{1118}; P[acman]-stbm-EGFP stbm^{6}/+	9	5041.42	4656 to 5427	2878.16	2555 to 3201	0.44	0.339 to 0.539	80.39	60.79 to 118.7	Fig.2R
dsh^{V26}/Y; P[acman]-dsh^{TEV}/P[acman]-stbm-EGFP stbm^{6} (2 hr HS)	6	4725.57	4134 to 5318	2857.97	1862 to 4061	0.41	0.353 to 0.459	33.98	0.297 to 0.333	Fig.2R
dsh^{V26}/Y; P[acman]-dsh^{TEV}/P[acman]-stbm-EGFP stbm^{6} (2 hr HS + 18°C)	6	4419.35	3962 to 4876	2711.56	2184 to 3207	0.40	0.367 to 0.429	52.24	41.71 to 69.89	Fig.2R
dsh^{V26}/Y; P[acman]-dsh^{TEV}/P[acman]-stbm-EGFP stbm^{6}; P[CaSpeR]-hs-TEVp/+ (No HS)	7	4732.28	3962 to 4876	2722.73	2168 to 3243	0.44	0.362 to 0.503	65.32	53.34 to 84.21	Fig.2R
dsh^{V26}/Y; P[acman]-dsh^{TEV}/P[acman]-stbm-EGFP stbm^{6}; P[CaSpeR]-hs-TEVp/+ (2 hr HS)	6	4386.05	4136 to 5329	2655.81	1438 to 3662	0.41	0.362 to 4.450	73.71	62.34 to 90.15	Fig.2R
dsh^{V26}/Y; P[acman]-dsh^{TEV}/P[acman]-stbm-EGFP stbm^{6}; P[CaSpeR]-hs-TEVp/+ (2 hr HS + 18°C)	6	4005.26	3708 to 5064	2379.36	1772 to 3058	0.42	0.403 to 0.431	86.84	69.55 to 115.6	Fig.2R
dsh^{V26} FRT19A/ ubi-mRFP-nls FRT19A; Ubx-FLP/P[acman]-stbm-EGFP stbm^{6}	6	3241.62	2331 to 4152	1754.66	994 to 2715	0.47	0.424 to 0.514	41.55	34.72 to 51.74	Fig.2R

EGFP-Dgo before and after Dsh^{TEV} cleavage

Genotype	n	Total fluorescent mean intensity	Total mean intensity confidence intervals	Stable amount	Stable amount 95% confidence intervals	y[max]	y[max] with 95% confidence intervals	t[0.5] Half-life	Half-life with 95% confidence Intervals	Figure
w^{1118}; P[acman-EGFP-dgo dgo^{380}/+	5	1246.17	1084 to 1408	739.64	618.5 to 860.8	0.42	0.364 to 0.477	45.80	39.27 to 54.94	Fig.S2S
dsh^{V26}/Y; P[acman]-dsh^{TEV}/P[acman]-EGFP-dgo dgo^{380}	5	1193.01	1010 to 1376	625.18	417.3 to 833.1	0.49	0.450 to 0.518	36.13	29.84 to 45.78	Fig.S2S
dsh^{V26}/Y; P[acman]-dsh^{TEV}/P[acman]EGFP-dgo dgo^{380}; P[CaSpeR]-hs-TEVp/+ (2 hr HS)	4	1010.81	664.8 to 1357	434.53	238.6 to 630.5	0.58	0.493 to 0.722	46.10	39.64 to 55.07	Fig.S2S

EGFP-Pk in wild-type Dsh and *dsh*^{V26} null tissue

Genotype	n	Total fluorescent mean intensity	Total mean intensity confidence intervals	Stable amount	Stable amount 95% confidence intervals	y[max]	y[max] with 95% confidence intervals	t[0.5] Half-life	Half-life with 95% confidence Intervals	Figure
w^{1118}; EGFP-pk/+	5	1166.00	173.5 to 1468	607.30	422.2 to 792.4	0.49	0.537 to 0.586	54.71	47.84 to 63.88	Fig.S2T
dsh^{V26} FRT19A/ubi-nls-RFP FRT19A; Ubx-FLP/P[acman]EGFP-pk	6	867.60	733 to 1002	274.26	187.1 to 361.4	0.69	0.662 to 0.727	59.83	52.1 to 70.24	Fig.S2T

Dsh-EGFP after Tom70-HA-vhhGFP acute knockdown with or without blocking Dynamin-dependent endocytosis

Genotype	n	Total fluorescent mean intensity	Total mean intensity confidence intervals	Stable amount	Stable amount 95% confidence intervals	y[max]	y[max] with 95% confidence intervals	t[0.5] Half-life	Half-life with 95% confidence Intervals	Figure
Ubx-FLP, dsh^{V26}/Y; P[acman]-dsh-EGFP FRT40/P[CaSpeR]-hs-Tom70-HA-vhhGFP FRT40 (No HS)	5	1844.65	1579.6 to 2109.7	1006.81	156.2 to 1257	0.46	0.419 to 0.587	45.77	28.4 to 117.8	Fig.4E
Ubx-FLP, dsh^{V26}/Y; P[acman]-dsh-EGFP FRT40/P[CaSpeR]-hs-Tom70-HA-vhhGFP FRT40 (2 hr HS)	7	1495.44	1077.4 to 1913.4	590.08	389.7 to 790.4	0.61	0.583 to 633	36.93	26.4 to 27.1	Fig.4E
dsh^{1}, shi^{ts1}, hs-FLP/Y; P[acman]-dsh-EGFP FRT40/hs-Tom70-HA-vhhGFP4 FRT40 (2 hr HS)	5	1977.66	2274.7 to 1680.7	1028.77	777.6 to 1280	0.49	0.407 to 0.532	79.19	62.33 to 108.5	Fig.4E

EGFP-Pk in the same cell as *dsh*^{V26} or in the neighbouring wild-type cell

Genotype	n	Total fluorescent mean intensity	Total mean intensity confidence intervals	Stable amount	Stable amount 95% confidence intervals	y[max]	y[max] with 95% confidence intervals	t[0.5] Half-life	Half-life with 95% confidence Intervals	Figure
EGFP-pk	9	1796.00	1509 to 2083	498.90	331.4 to 666.5	0.71	0.680 to 0.746	45.01	38.48 to 54.2	Fig.S4I
EGFP-pk dsh^{V26}	7	1612.00	1121 to 2103	237.90	79.24 to 396.5	0.83	0.801 to 0.855	42.62	38.01 to 48.49	Fig.S4I
EGFP-pk dsh^{V26} clone boundary next to pk$^{pk\text{-}sple13}$	8	999.30	799.7 to 1199	353.40	223 to 483.9	0.68	0.649 to 0.705	25.94	21.75 to 32.11	Fig.S4I
EGFP-pk clone boundary next to dsh^{V26} pk$^{pk\text{-}sple13}$	9	594.10	468.9 to 719.3	151.30	90.34 to 212.3	0.68	0.652 to 0.707	23.96	20.02 to 29.81	Fig.S4I

Table S2. Quantitative FRAP data. Related to Figures 2, S2, 4, S4.

Genotypes for each FRAP experiment, along with the timepoints at which the pupal wings were imaged are shown, along with the number (n) of wings imaged for each genotype, the total fluorescence intensity, the stable amounts, the y[max] (plateau), the half-life and the 95% confidence intervals for each of the data sets. The figure panel relating to the results is also indicated.

**Dsh acute knockdown using TEVp, Tom70-HA-vhhGFP or Rpn10-HA-vhhGFP: Compared stable amount, Fig.S1F**					
Unpaired t-tests	**Mean Diff.**	**95% CI of diff.**	**Significant?**	**Summary**	**Adjusted p Value**
Dsh^{TEV} no heat shock ***vs.*** *Dsh*^{TEV} 2 hr heat shock + 1 hr recovery	-0.891	-1.011 to -0.788	Yes	***	≤0.0001
Dsh-EGFP with *Tom70-vhhGFP* no heat shock ***vs.*** *Dsh-EGFP* with *Tom70-vhhGFP* 2 hr heat shock	1.265	-0.303 to 2.833	Yes	***	≤0.0001
Dsh-EGFP with *Rpn10-vhhGFP* no heat shock ***vs.*** *Dsh-EGFP* with *Rpn10-vhhGFP* 90 min heat shock	-0.772	-1.111 to -0.432	Yes	**	0.0032
**Fz mean vector polarity: Compared stable amount, Fig.2M**					
ANOVA, Tukey-Kramer’s multiple comparison test	**Mean Diff.**	**95% CI of diff.**	**Significant?**	**Summary**	**Adjusted p Value**
dsh^{TEV} x *TEVp* no heat shock ***vs.*** *dsh*^{TEV} x *TEVp* heat shock	0.322	0.193 to 0.451	Yes	***	≤0.0001
dsh^{TEV} x *TEVp* no heat shock ***vs.*** *dsh*^{TEV} x *TEVp* heat shock + 1 hr recovery 18°C	0.439	0.310 to 0.569	Yes	***	≤0.0001
dsh^{TEV} x *TEVp* no heat shock ***vs.*** *dsh*^{TEV} x *TEVp* heat shock + 2 hr recovery 18°C	0.453	0.324 to 0.582	Yes	***	≤0.0001
dsh^{TEV} x TEVp no heat shock ***vs.*** *dsh*^{TEV} x *TEVp* heat shock + 3 hr recovery 18°C	0.471	0.342 to 0.600	Yes	***	≤0.0001
dsh^{TEV} x TEVp no heat shock ***vs.*** *dsh*^{V26}	0.409	0.280 to 0.538	Yes	***	≤0.0001
**Fmi mean vector polarity: Compared stable amount, Fig.2O**					
ANOVA, Tukey-Kramer’s multiple comparison test	**Mean Diff.**	**95% CI of diff.**	**Significant?**	**Summary**	**Adjusted p Value**
dsh^{TEV} x *TEVp* no heat shock ***vs.*** *dsh*^{TEV} x *TEVp* heat shock	0.065	0.017 to 0.193	No	na	0.055
dsh^{TEV} x *TEVp* no heat shock ***vs.*** *dsh*^{TEV} x *TEVp* heat shock + 1 hr recovery 18°C	0.275	0.169 to 0.381	Yes	***	≤0.0001
dsh^{TEV} x *TEVp* no heat shock ***vs.*** *dsh*^{TEV} x *TEVp* heat shock + 2 hr recovery 18°C	0.427	0.321 to 0.533	Yes	***	≤0.0001
dsh^{TEV} x TEVp no heat shock ***vs.*** *dsh*^{TEV} x *TEVp* heat shock + 3 hr recovery 18°C	0.431	0.325 to 0.537	Yes	***	≤0.0001
dsh^{TEV} x TEVp no heat shock ***vs.*** *dsh*^{V26}	0.473	0.351 to 0.595	Yes	***	≤0.0001
**Stbm mean vector polarity: Compared stable amount, Fig.2R**					
ANOVA, Tukey-Kramer’s multiple comparison test	**Mean Diff.**	**95% CI of diff.**	**Significant?**	**Summary**	**Adjusted p Value**
dsh^{TEV} x *TEVp* no heat shock ***vs.*** *dsh*^{TEV} x *TEVp* heat shock	-0.020	-0.166 to 0.126	No	ns	>0.9999
dsh^{TEV} x *TEVp* no heat shock ***vs.*** *dsh*^{TEV} x *TEVp* heat shock + 1 hr recovery 18°C	0.369	0.224 to 0.515	Yes	***	≤0.0001
dsh^{TEV} x *TEVp* no heat shock ***vs.*** *dsh*^{TEV} x *TEVp* heat shock + 2 hr recovery 18°C	0.343	0.188 to 0.499	Yes	***	≤0.0001
dsh^{TEV} x TEVp no heat shock ***vs.*** *dsh*^{TEV} x *TEVp* heat shock + 3 hr recovery 18°C	0.334	0.179 to 0.489	Yes	***	≤0.0001
dsh^{TEV} x TEVp no heat shock ***vs.*** *dsh*^{V26}	0.444	0.292 to 0.596	Yes	***	≤0.0001
**Fz-EGFP before and after cleavage of DshTEV (FRAP): Compared stable amount, Fig.2N**					
ANOVA, Tukey-Kramer’s multiple comparison test	**Mean Diff.**	**95% CI of diff.**	**Significant?**	**Summary**	**Adjusted p Value**
w^{1118}*; fz-EGFP/+* ***vs.*** *dsh*^{V26} *FRT19A/ubi-mRFP-nls FRT19A; Ubx-FLP/+; fz-EGFP/+*	754.2	328.6 to 1180	Yes	***	≤0.0001
w^{1118}*; fz-EGFP/+* ***vs.*** *dsh*^{V26}*/Y; P[acman]-dsh3xTEV/P[CaSpeR]-hs-TEVp; fz-EGFP/+* (no HS)	222.8	-168.2 to 613.9	No	ns	0.613
dsh^{V26}*/Y; P[acman]-dsh3xTEV/+; fz-EGFP/+* (HS) ***vs.*** *dsh*^{V26}*/Y; P[acman]-dsh3xTEV/P[CaSpeR]-hs-TEVp; fz-EGFP/+* (HS)	338.8	-52.28 to 729.8	Yes	*	0.043
dsh^{V26}*/Y; P[acman]-dsh3xTEV/+; fz-EGFP/+* (HS + 1 hr 18°C) ***vs.*** *dsh*^{V26}*/Y; P[acman]-dsh3xTEV/ P[CaSpeR]-hs-TEVp; fz-EGFP/+* (HS + 1 hr 18°C)	610.2	184.6 to 1036	Yes	**	0.001
**Fmi-EGFP before and after cleavage of DshTEV (FRAP): Compared stable amount, Fig.2P**					
ANOVA, Tukey-Kramer’s multiple comparison test	**Mean Diff.**	**95% CI of diff.**	**Significant?**	**Summary**	**Adjusted p Value**
w^{1118}*; fmi-EGFP/+* ***vs.*** *dsh*^{V26} *FRT19A/ubi-mRFP-nls FRT19A; Ubx-FLP/fmi-EGFP*	720.7	424.2 to 1017	Yes	***	≤0.0001
w^{1118}*; fmi-EGFP/+* ***vs.*** *dsh*^{V26}*/Y; P[acman]-dsh*^{TEV}*/fmi-EGFP; P[CaSpeR]-hs-TEVp/+* (no HS)	34.1	-249.8 to 318.1	No	ns	0.997
dsh^{V26}*/Y; P[acman]-dsh*^{TEV}*//fmi-EGFP* (HS) ***vs.*** *dshV26/Y; P[acman]-dsh*^{TEV}*//fmi-EGFP; P[CaSpeR]-hs-TEVp/+* (HS)	185.7	-98.26 to 469.6	No	ns	0.347
dsh^{V26}*/Y; P[acman]-dsh*^{TEV}*//fmi-EGFP* (HS + 1 hr 18°C) ***vs.*** *dsh*^{V26}*/Y; P[acman]-dsh*^{TEV}*/fmi-EGFP; P[CaSpeR]-hs-TEVp/+* (HS + 1 hr 18°C)	350.5	66.54 to 634.4	Yes	**	0.009
**Stbm-EGFP before and after cleavage of DshTEV (FRAP): Compared stable amount, Fig.2R**					
ANOVA, Tukey-Kramer’s multiple comparison test	**Mean Diff.**	**95% CI of diff.**	**Significant?**	**Summary**	**Adjusted p Value**
w^{1118}*; P[acman]-stbm-EGFP stbm*^{6}*/+* ***vs.*** *dsh*^{V26} *FRT19A/ubi-mRFP-nls FRT19A; Ubx-FLP/P[acman]-stbm-EGFP stbm*^{6}	1024	12.67 to 2034	Yes	*	0.046
w^{1118}*; P[acman]-stbm-EGFP stbm*^{6}*/+* ***vs.*** *dsh*^{V26}*/Y; P[acman]-dsh*^{TEV}*//P[acman]-stbm-EGFP stbm*^{6}*; P[CaSpeR]-hs-TEVp/+* (no HS)	172.5	-897.2 to 1242	No	ns	0.999
dsh^{V26}*/Y; P[acman]-dsh*^{TEV}*//P[acman]-stbm-EGFP stbm*^{6} (HS) ***vs.*** *dsh*^{V26}*/Y; P[acman]-dsh*^{TEV}*/P[acman]-stbm-EGFP stbm*^{6}*; P[CaSpeR]-hs-TEVp/+* (HS)	411.1	-875.5 to 1698	No	ns	0.949
dsh^{V26}*/Y; P[acman]-dsh*^{TEV}*//P[acman]-stbm-EGFP stbm*^{6} (HS + 1 hr 18°C) ***vs.*** *dsh*^{V26}*/Y; P[acman]-dsh*^{TEV}*/ P[acman]-stbm-EGFP stbm*^{6}*; P[CaSpeR]-hs-TEVp/+* (HS + 1hr 18°C)	280.8	-932.2 to 1494	No	ns	0.990
**Stbm-EGFP before and after cleavage of DshTEV (FRAP): Compared stable amount, Fig.S2A-E**					
**Panel A Western of *DshTEV* acute knockdown**	**Mean Diff.**	**95% CI of diff.**	**Significant?**	**Summary**	**Adjusted p Value**
Dunnett's multiple comparisons test					
Dsh No heat shock **vs.** Dsh Control heat shock	-0.098	-2.40 to 2.2	No	ns	0.993
Dsh No heat shock **vs.** Dsh Control heat shock + 1 hr at 18°C	-0.498	-4.346 to 3.35	No	ns	0.817
Dsh No heat shock **vs.** Dsh 2 hr heat shock	0.474	-0.177 to 1.124	No	ns	0.091
Dsh No heat shock **vs.** Dsh 2 hr heat shock + 1 hr at 18°C	0.910	0.668 to 1.152	Yes	**	0.004
**Panel B Western of DshTEV acute knockdown**					
Dunnett's multiple comparisons test					
Fz No heat shock **vs.** Fz Control heat shock	0.11	-0.757 to 0.978	No	ns	0.824
Fz No heat shock **vs.** Fz Control heat shock + 1 hr at 18°C	0.202	-0.624 to 1.027	No	ns	0.496
Fz No heat shock **vs.** Fz 2 hr heat shock	-0.231	-3.334 to 2.872	No	ns	0.956
Fz No heat shock **vs.** Fz 2 hr heat shock + 1 hr at 18°C	0.279	-1.883 to 2.441	No	ns	0.819
**Panel C Western of DshTEV acute knockdown**					

Dunnett's multiple comparisons test					
Fmi No heat shock **vs.** Fmi Control heat shock	-0.382	-6.111 to 5.347	No	ns	0.969
Fmi No heat shock **vs.** Fmi Control heat shock + 1 hr at 18°C	-1.124	-10.98 to 8.73	No	ns	0.862
Fmi No heat shock **vs.** Fmi 2 hr heat shock	-0.332	-1.65 to 0.986	No	ns	0.478
Fmi No heat shock **vs.** Fmi 2 hr heat shock + 1 hr at 18°C	-0.166	-0.461 to 0.128	No	ns	0.143
**Panel D Western of DshTEV acute knockdown**					
Dunnett's multiple comparisons test					
Stbm No heat shock **vs.** Stbm Control heat shock	0.241	-0.160 to 0.641	No	ns	0.129
Stbm No heat shock **vs.** Stbm Control heat shock + 1 hr at 18°C	0.098	-0.613 to 0.810	No	ns	0.791
Stbm No heat shock **vs.** Stbm 2 hr heat shock	-0.1003	-1.786 to 1.586	No	ns	0.979
Stbm No heat shock **vs.** Stbm 2 hr heat shock + 1 hr at 18°C	0.0733	-0.414 to 0.560	No	ns	0.753
**Panel E Western of DshTEV acute knockdown**					
Dunnett's multiple comparisons test					
Pk No heat shock **vs.** Pk Control heat shock	-0.21	-2.774 to 2.354	No	ns	0.942
Pk No heat shock **vs.** Pk Control heat shock + 1 hr at 18°C	-0.003	-1.866 to 1.861	No	ns	0.999
Pk No heat shock **vs.** Pk 2 hr heat shock	0.105	-1.028 to 1.238	No	ns	0.918
Pk No heat shock **vs.** Pk 2 hr heat shock + 1 hr at 18°C	0.329	-0.749 to 1.406	No	ns	0.377
**Fmi (F) and Fz (G) mean vector polarity values after disruption of Dsh-EGFP using vhhGFP: Compared stable amount, Fig.S2F-G**					
**Panel F acute knockdown Fmi mean vector polarity**					
ANOVA, Tukey-Kramer's multiple comparison test	**Mean Diff.**	**95% CI of diff.**	**Significant?**	**Summary**	**Adjusted P Value**
dsh-EGFP No heat shock ***vs.*** *dsh-EGFP* 2 hr heat shock	0.039	-0.117 to 0.195	No	ns	0.999
dsh-EGFP No heat shock ***vs.*** *dsh-EGFP* 2 hr heat shock + 1h 18°C	0.055	-0.101 to 0.210	No	ns	0.987
dsh-EGFP Tom70vhh No heat shock ***vs.*** *dsh-EGFP Tom70vhh* 2 hr heat shock	0.134	-0.022 to 0.290	No	ns	0.163
dsh-EGFP Tom70vhh No heat shock ***vs.*** *dsh-EGFP Tom70vhh* 2 hr heat shock + 1 hr 18°C	0.278	0.122 to 0.434	Yes	***	≤0.0001
dsh-EGFP Rpn10vhh No heat shock ***vs.*** *dsh-EGFP Rpn10vhh* 2 hr heat shock	-0.1004	-0.251 to 0.050	No	ns	0.524
dsh-EGFP Rpn10vhh No heat shock ***vs.*** *dsh-EGFP Rpn10vhh* 2 hr heat shock + 1 hr 18°C	0.374	0.218 to 0.530	Yes	***	≤0.0001
**Panel G acute knockdown Fz mean vector polarity**					
ANOVA, Tukey-Kramer's multiple comparison test	**Mean Diff.**	**95% CI of diff.**	**Significant?**	**Summary**	**Adjusted p Value**
dsh-EGFP No heat shock ***vs.*** *dsh-EGFP* 2 hr heat shock	0.006	-0.139 to 0.151	No	ns	>0.9999
dsh-EGFP No heat shock ***vs.*** *dsh-EGFP* 2 hr heat shock + 1 hr 18°C	0.06	-0.085 to 0.206	No	ns	0.955
dsh-EGFP Tom70vhh No heat shock ***vs.*** *dsh-EGFP Tom70vhh* 2 hr heat shock	0.208	0.106 to 0.450	Yes	**	0.0095
dsh-EGFP Tom70vhh No heat shock ***vs.*** *dsh-EGFP Tom70vhh* 2 hr heat shock + 1 hr 18°C	0.333	0.162 to 0.505	Yes	***	≤0.0001
dsh-EGFP Rpn10vhh No heat shock ***vs.*** *dsh-EGFP Rpn10vhh* 2 hr heat shock	0.331	0.159 to 0.503	Yes	***	≤0.0001
dsh-EGFP Rpn10vhh No heat shock ***vs.*** *dsh-EGFP Rpn10vhh* 2 hr heat shock + 1 hr 18°C	0.431	0.259 to 0.603	Yes	***	≤0.0001
**EGFP-Dgo before and after DshTEV cleavage (FRAP): Compared stable amount, Fig.S2S**					
ANOVA, Tukey-Kramer's multiple comparison test	**Mean Diff.**	**95% CI of diff.**	**Significant?**	**Summary**	**Adjusted P Value**
w^{1118}*; P[acman-EGFP-dgo* dgo^{380}*/+* ***vs.*** dsh^{V26}*/Y; P[acman]-*dsh^{TEV}*/P[acman-EGFP-dgo]* dgo^{380} (HS)	114.5	-106.4 to 335.3	No	ns	0.380
w^{1118}*; P[acman-EGFP-dgo* dgo^{380}*/+* ***vs.*** dsh^{V26}*/Y; P[acman]-*dsh^{TEV}*/P[acman-EGFP-dgo]* dgo^{380}*; P[CaSpeR]-hs-TEVp/+* (HS)	305.1	69.69 to 540.5	Yes	*	0.012
**EGFP-Pk in wild-type Dsh and dsh null tissue (FRAP): Compared stable amount, Fig.S2T**					
Unpaired t-test	**Mean Diff.**	**95% CI of diff.**	**Significant?**	**Summary**	**Adjusted P Value**
w^{1118}*; EGFP-pk/+* ***vs.*** dsh^{V26} *FRT19A/ubi-nls-RFP FRT19A; Ubx-FLP/P[acman]EGFP-pk*	607.3	-493.4 to -172.7	Yes	**	0.001
**Distal propergation of Pk: Compared stable amount, Fig.4C**					
ANOVA, Dunnett's multiple comparison	**Mean Diff.**	**95% CI of diff.**	**Significant?**	**Summary**	**Adjusted P Value**
Cell inside the clone **vs.** 1st cell outside the clone	0.155	-0.130 to 0.441	No	ns	0.606
Cell inside the clone **vs.** 2nd cell outside the clone	0.287	0.001 to 0.573	Yes	*	0.049
Cell inside the clone **vs.** 3rd cell outside the clone	0.487	0.201 to 0.773	Yes	***	0.0002
Cell inside the clone **vs.** 4th cell outside the clone	0.619	0.333 to 0.905	Yes	***	≤0.0001
Cell inside the clone **vs.** 5th cell outside the clone	0.67	0.385 to 0.956	Yes	***	≤0.0001
**Proximal propergation of Pk: Compared stable amount, Fig.4D**					
ANOVA, Dunnett's multiple comparison	**Mean Diff.**	**95% CI of diff.**	**Significant?**	**Summary**	**Adjusted P Value**
Cell inside the clone edge **vs.** 1st cell outside the clone	0.706	0.469 to 0.944	Yes	***	≤0.0001
Cell inside the clone edge **vs.** 2nd cell outside the clone	0.742	0.504 to 0.979	Yes	***	≤0.0001
Cell inside the clone edge **vs.** 3rd cell outside the clone	0.795	0.558 to 1.032	Yes	***	≤0.0001
**Dsh-EGFP after Tom70-HA-vhhGFP acute knockdown with or without blocking Dynamin-dependent endocytosis: Compared stable amount, Fig.4E**					
ANOVA, Tukey-Kramer's multiple comparison test	**Mean Diff.**	**95% CI of diff.**	**Significant?**	**Summary**	**Adjusted p Value**
UbxFLP, dsh^{V26}*/Y; P[acman]-dsh-EGFP FRT40/P[CaSpeR]-hs-Tom70-HA-vhhGFP FRT40 (No Tom70vhh)* ***vs.*** *the cell neighbouring the knockdown clone cells*	416.7	97.29 to 736.2	Yes	*	0.011
UbxFLP, dsh^{V26}*/Y; P[acman]-dsh-EGFP FRT40/P[CaSpeR]-hs-Tom70-HA-vhhGFP FRT40 (Cell neighbouring cell knockdown clone)* ***vs.*** *the cell neighbouring the knockdown clone cells in a* shi^{ts1} *background*	-438.7	-758.1 to -119.2	Yes	**	0.008
**Quantitation of Dsh-EGFP and Dsh levels with or without Tom70vhh or Rpn10vhh: Compared stable amount, Fig.S4A**					
Unpaired t-test	**Mean Diff.**	**95% CI of diff.**	**Significant?**	**Summary**	**Adjusted P Value**
Dsh-EGFP Tom70vhh No heat shock ***vs.*** *Dsh-EGFP Tom70vhh* 2 hr heat shock	1.579	-0.288 to 3.447	No	ns	0.079
Dsh Tom70vhh No heat shock ***vs.*** *Dsh Tom70vhh* 2 hr heat shock	1.381	-0.915 to 3.677	No	ns	0.17
Dsh-EGFP Rpn10vhh No heat shock ***vs.*** *Dsh-EGFP Rpn10vhh* 90 min heat shock	-0.444	-0.781 to -0.108	Yes	*	0.022
Dsh Rpn10vhh No heat shock ***vs.*** *Dsh Rpn10vhh* 90 min heat shock	0.034	-0.649 to 0.718	No	ns	0.896
**Dsh-EGFP clones in Tom70-vhh knockdown tissue: Compared stable amount, Fig.S4B**					

Paired t-test	Mean Diff.	95% CI of diff.	Significant?	Summary	Adjusted P Value
Proximodistal membrane localised Pk in cells outside of the clone **vs.** *At the proximal edge of cells at the proximal edge of Dsh-EGFP clones*	4.925	-1.4 to 11.25	No	ns	0.108
***EGFP-Pk* in the same cell as d*sh*^{V26} or in the neighbouring wild-type cell: Compared stable amount, Fig.S4I**					
ANOVA, Sidak's multiple comparisons test	**Mean Diff.**	**95% CI of diff.**	**Significant?**	**Summary**	**Adjusted P Value**
(2nd column) *EGFP-pk* in a wild-type cell next to *pk*$^{pk\text{-}sple13}$ *dsh*^{V26} cells n=9 **vs.** (4th column) *EGFP-pk* in a wild-type wing n=9	-347.6	-536.4 to -158.8	Yes	***	0.0002
(2nd column) *EGFP-pk* in a wild-type cell next to *pk*$^{pk\text{-}sple13}$ *dsh*^{V26} cells n=9 **vs.** (1st column) *EGFP-pk in a dsh*^{V26} mutant cell next to *pk*$^{pk\text{-}sple13}$ *dsh* wild-type cells n=8	-202.1	-397.1 to -7.171	Yes	*	0.04
(1st column) *EGFP-pk* in a *dsh*^{V26} mutant cell next to *pk*$^{pk\text{-}sple13}$ mutant *dsh* wild-type cells n=8 **vs.** (4th column) EGFP-pk in a wild-type wing n=9	115.5	-97.15 to 328.2	No	ns	0.209
**Pk membrane intensity on and near *dsh*^{V26} clones: Compared stable amount, Fig.S4J**					
ANOVA, Tukey-Kramer's multiple comparison test	**Mean Diff.**	**95% CI of diff.**	**Significant?**	**Summary**	**Adjusted P Value**
outside *dsh*^{V26} tissue ***vs.*** Distal edge of the clone	9.175	0.700 to 17.65	Yes	*	0.034
outside *dsh*^{V26} tissue **vs.** One distal cell away form the clone	7.09	-0.137 to 14.32	No	ns	0.055
outside *dsh*^{V26} tissue **vs.** Two distal cell away form the clone	5.101	-0.371 to 10.57	No	ns	0.068
outside *dsh*^{V26} tissue **vs.** Three distal cell away form the clone	2.07	-3.25 to 7.385	No	ns	0.664

Table S3. Detailed statistical comparisons. Related to Figures 2, 4, S2, S4.

Detailed results for the statistical tests carried out on the data shown in the figures are shown. The statistical test (either ANOVA or t-test is indicated), as well as the names of the post hoc tests. These include the Dunnett's multiple comparisons test (for comparing the control to the other experimental conditions), and the Šídák's multiple comparisons test (to compare pre-selected pairs of conditions within an experiment). The table also shows the mean difference and 95% confidence intervals. Stars indicate statistical significance *$P \leq 0.05$ **$P \leq 0.01$ and ***$P \leq 0.001$. The figure panel relating to each statistical test is also indicated.
