# Supplementary material for: Rapid Disruption of Dishevelled Activity Uncovers an Intercellular Role in Maintenance of Prickle in Core Planar Polarity Protein Complexes
Source: Cell Rep. 2018 Nov 6;25(6):1415–1424.e6. doi: 10.1016/j.celrep.2018.10.039 (PMC6231328; doi:10.1016/j.celrep.2018.10.039)
Supplement: Document S2. Article plus Supplemental Information [file mmc2.pdf]

Cell Reports

Report

Rapid Disruption of Dishevelled Activity Uncovers an Intercellular Role in Maintenance of Prickle in Core Planar Polarity Protein Complexes

Graphical Abstract

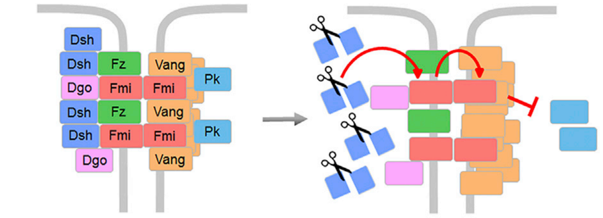

Prickle (Pk) dissociates from intercellular protein complexes upon Dishevelled (Dsh) acute knockdown

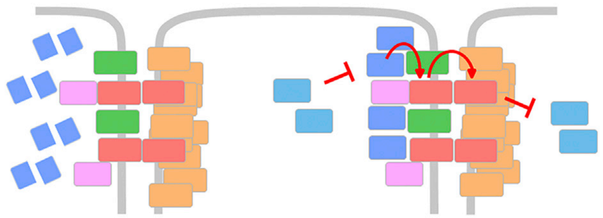

Cytoplasmic Pk destabilizes Dsh in intracellular core protein complexes leading to the release of Pk in the next cell, resulting in a propagating wave of complex disassembly

Highlights

- Inducible genetic tools can efficiently disrupt Dishevelled activity *in vivo*
- Dishevelled activity continuously promotes core planar polarity complex stability
- Prickle is maintained in intercellular complexes cell non-autonomously by Dishevelled
- Unbound Prickle results in intercellular propagation of complex destabilization

Authors

Margarida Ressurreição, Samantha Warrington, David Strutt

Correspondence

d.strutt@sheffield.ac.uk

In Brief

Ressurreição et al. have developed tools to rapidly disrupt protein function in *Drosophila* and used these to investigate the function of the protein Dishevelled in planar polarity. They find a continuous requirement for Dishevelled in core planar polarity complex maintenance with its loss causing relocalization of Prickle in neighboring cells.

Ressurreição et al., 2018, Cell Reports *25*, 1415–1424
November 6, 2018 © 2018 The Author(s).
https://doi.org/10.1016/j.celrep.2018.10.039

Cell Reports
Report

OPEN ACCESS
CellPress

Rapid Disruption of Dishevelled Activity Uncovers an Intercellular Role in Maintenance of Prickle in Core Planar Polarity Protein Complexes

Margarida Ressurreição,[1] Samantha Warrington,[1] and David Strutt[1,2,*]
[1]Department of Biomedical Science, University of Sheffield, Western Bank, Sheffield, S10 2TN, UK
[2]Lead Contact
*Correspondence: d.strutt@sheffield.ac.uk
https://doi.org/10.1016/j.celrep.2018.10.039

SUMMARY

Planar polarity, the coordinated polarization of cells in the plane of a tissue, is important for normal tissue development and function. Proteins of the core planar polarity pathway become asymmetrically localized at the junctions between cells to form intercellular complexes that coordinate planar polarity between cell neighbors. Here, we combine tools to rapidly disrupt the activity of the core planar polarity protein Dishevelled, with quantitative measurements of protein dynamics and levels, and mosaic analysis, to investigate Dishevelled function in maintenance of planar polarity. We provide mechanistic insight into the hierarchical relationship of Dishevelled with other members of the core planar polarity complex. Notably, we show that removal of Dishevelled in one cell causes rapid release of Prickle into the cytoplasm in the neighboring cell. This release of Prickle generates a self-propagating wave of planar polarity complex destabilization across the tissue. Thus, Dishevelled actively maintains complex integrity across intercellular junctions.

INTRODUCTION

Planar polarity is the coordination of cell polarity within the plane of a tissue (Goodrich and Strutt, 2011; Butler and Wallingford, 2017). It is most obviously manifested by the orientation of trichomes and bristles in *Drosophila* or hair structures in the inner ear and skin of vertebrates (Goodrich and Strutt, 2011; Devenport, 2016). Importantly, disruptions in planar polarity have been linked to congenital birth defects and cancer (Butler and Wallingford, 2017).

At the molecular level, planar polarity is defined as the asymmetric subcellular distribution of planar polarity proteins. During *Drosophila* wing development, the six proteins of the "core" planar polarity pathway ("core proteins" hereafter) self-organize along the proximodistal axis into stable asymmetric intercellular complexes (Figure 1A) of variable stoichiometry (Strutt et al., 2016). The transmembrane protein Frizzled (Fz) and the cytoplasmic proteins Dishevelled (Dsh) and Diego (Dgo) co-localize at distal junctions, while the fourpass transmembrane protein Strabismus (Stbm; also known as Van Gogh [Vang]) and the LIM-domain protein Prickle (Pk) co-localize proximally. The atypical cadherin Flamingo (Fmi; also known as Starry Night [Stan]) localizes both proximally and distally, bridging the two halves of the complex (reviewed in Strutt and Strutt, 2009). For intercellular complexes to form and distribute to opposite cell ends, activity of all six core proteins is required to enable feedback interactions that are thought to amplify cellular asymmetry (Strutt and Strutt, 2009; Warrington et al., 2017).

Dsh (Dvl in mammals) is a multifunctional protein that is a key cytoplasmic component of both the core planar polarity and Wnt signaling pathways and consequently has been intensely studied. Nevertheless, key questions remain regarding its cellular functions, and in particular its role in planar polarity feedback regulation is not well understood.

Although classical loss- and gain-of-function approaches were key in identifying the members of the core planar polarity complex, these techniques are not always suitable for dissecting molecular mechanisms, because of the effects of pleiotropy and adaptation (Hoeller et al., 2014; Warrington et al., 2017). To circumvent these limitations, technologies have been developed that can regulate *in vivo* protein function in a rapid and temporally controlled manner. These have proved successful in many biological systems including *Drosophila,* providing insights into developmental processes (Harder et al., 2008; Pauli et al., 2008; Caussinus et al., 2011; Warrington et al., 2017). In this study we optimized tools based on protein cleavage using tobacco etch virus protease (TEVp) (Harder et al., 2008; Pauli et al., 2008), and degradation or relocalization with anti-GFP nanobodies (Caussinus et al., 2011; Harmansa et al., 2017), to acutely disrupt Dsh activity *in vivo*.

RESULTS

Disruption of Dsh Activity *In Vivo* in the *Drosophila* Pupal Wing Epithelium

We optimized two technologies based on either TEVp-induced cleavage or targeting by anti-GFP nanobodies (vhhGFPs) (Figures 1B, 1E, and S1B). The vhhGFPs were HA-tagged and fused to either the Tom70 import signal or Rpn10, which act to relocalize target proteins to the mitochondria (Robinson et al., 2010) or proteasome (Janse et al., 2004), respectively (Figures 1E and S1B). For TEVp-mediated knockdown, TEVp recognition sites

This is an open access article under the CC BY license (http://creativecommons.org/licenses/by/4.0/).

OPEN ACCESS
CellPress

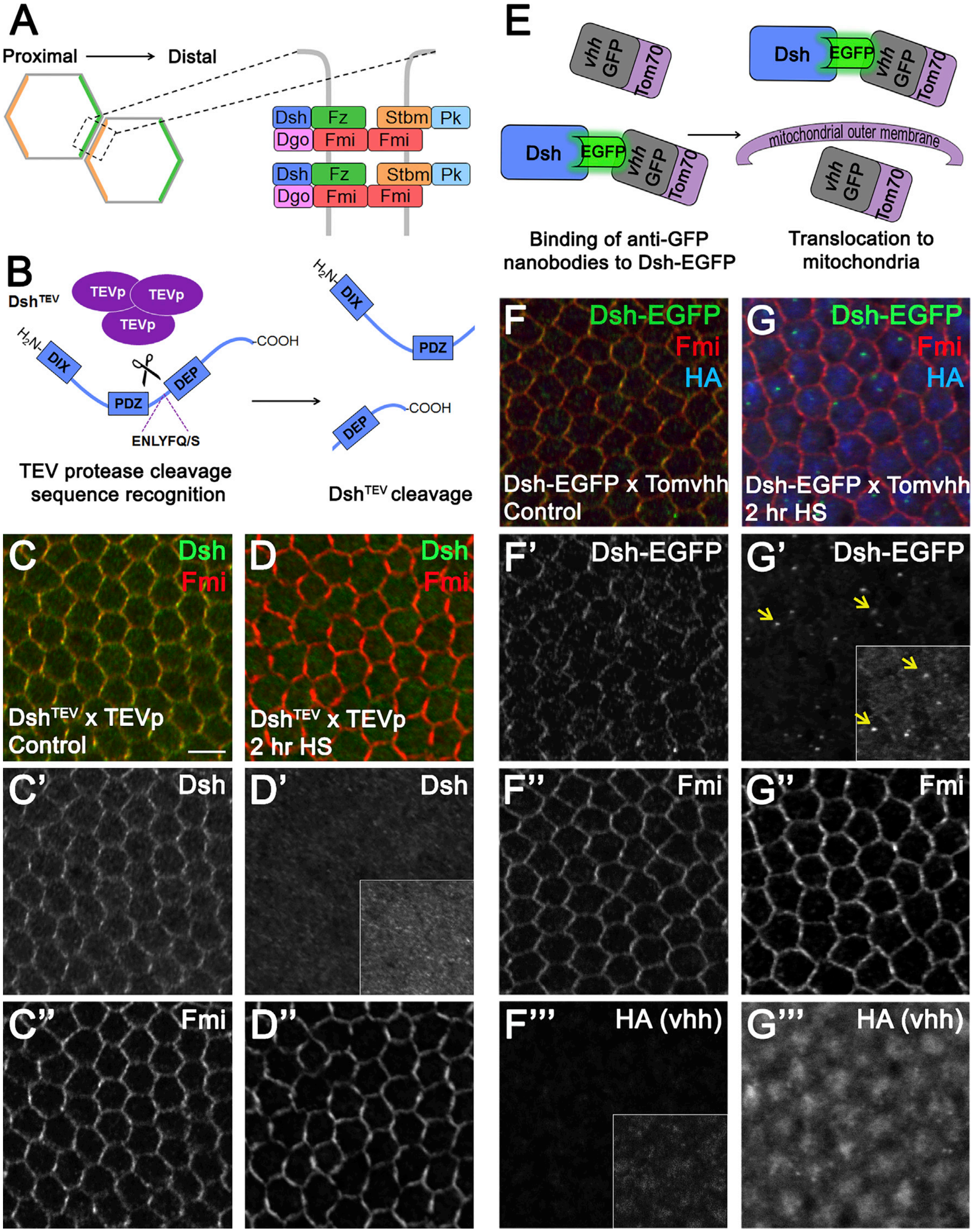

Figure 1. *In Vivo* Disruption of Dishevelled in the *Drosophila* Pupal Wing

(A) Graphical representation of cells of the wing epithelium depicting the asymmetric distribution of the "core" planar polarity proteins.

(B) Schematic representation of Dsh^{TEV} disruption by heat shock-induced TEVp-induced cleavage at introduced TEVp cleavage sites.

(C and D) Twenty-eight hours APF wing epithelium heterozygous for *dsh^{TEV}* and *hs-TEVp* transgenes in a *dsh^{V26}*-null background. (C) In the absence of heat-shock, no *hs-TEVp* is expressed, and the *dsh^{TEV}* transgene rescues the *dsh*-null phenotype, shown by the asymmetric localization of Dsh^{TEV} (green, C′) and endogenous Fmi (red, C″). (D) After expression of TEVp by a 2 hr heat shock at 38°C, Dsh^{TEV} localization is absent from the cell membrane (D′), but Fmi localization is maintained (D″). Scale bar, 5 μm.

(E) Dsh-EGFP disruption on the basis of targeting with anti-GFP nanobodies fused to the Tom70 mitochondrial translocation signal. Upon Tom70-HA-vhhGFP production via heat shock, Dsh-EGFP is displaced from cell junctions.

(legend continued on next page)

were introduced into the Dsh coding sequence (Figure 1B). TEVp and the vhhGFP fusions were acutely expressed under control of the *hsp70* promoter in transgenic flies, via heat shock at 38°C (Figure S1A). In the absence of heat shock (control conditions), Dsh^{TEV} and Dsh-EGFP rescued the *dsh*-null phenotype, resulting in normal planar polarization (Figures 1C,1F, and S1C, compare with Figure S2J). As a further control, HA antibody labeling confirmed that the vhhGFP fusions were not expressed when pupae were maintained at 25°C in the absence of heat-shock (Figures 1F‴ and S1C‴). To exclude non-specific effects, w^{1118} flies were exposed to the same heat-shock regimes, and vhhGFPs and TEVp were produced in the absence of Dsh-EGFP or Dsh^{TEV}, respectively (Table S1; Figures S3G–S3J); in all cases examined there was no significant change in core protein localization.

To rapidly disrupt Dsh^{TEV} via TEVp cleavage, we administered a 2 hr heat shock (38°C). Under these conditions and using an antibody that detects the C terminus of Dsh, Dsh^{TEV} localization at cell junctions was lost (Figures 1D and 1D′), while Fmi still remained asymmetrically localized at the cell membrane (Figure 1D″). This was accompanied by a significant loss in total cellular levels of Dsh protein, indicating that the cleaved protein was degraded (Figures S1E, S1F, and S2A).

Similarly, substantial depletion of Dsh-EGFP from junctions via Tom70-HA-vhhGFP and Rpn10-HA-vhhGFP expression was observed after 2 hr or 90 min heat shock, respectively (Figures 1G and S1D). HA labeling of Tom70-HA-vhhGFP was restricted to the cytoplasm (Figure 1G‴), while Dsh-EGFP was detected primarily in punctate spots within the cell (Figure 1G′, yellow arrows). However, Tom70-HA-vhhGFP did not reduce Dsh-EGFP total cellular levels (Figures S1E, S1F, and S4A), most likely because of sequestration of Dsh-EGFP to mitochondria. Upon production of Rpn10-HA-vhhGFP, Dsh-EGFP membrane localization was abolished, with HA labeling present throughout the cell (Figures S1D). This was accompanied by a significant loss in total cellular levels of Dsh-EGFP protein, indicating that the targeted protein is degraded (Figures S1E, S1F, and S4A).

We then compared the efficiency of depletion of Dsh using *UAS-TEVp* or *UAS-dsh-RNAi* (Figures S1A and S1G–S1L). Our attempts to knock down Dsh using RNAi were less efficient than the TEVp or vhhGFP methods (Figures S1G–S1J). Dsh was still detectable at cell junctions 6 hr after RNAi expression using two separate RNAi transgenes (Figures S1G–S1J). Furthermore, 4 hr of induced *GAL4/UAS* driven TEVp expression was not sufficient to abolish Dsh^{TEV} localization (Figures S1K and S1L).

Targeted Disruption of Dsh Activity Differentially Affects the Transmembrane Core Complex Components

After confirming that our techniques were able to remove Dsh protein from junctions within 2 hr, we went on to examine the effects on the polarity and stability of the transmembrane core proteins (Fz, Fmi, and Stbm). We examined their polarization prior to cleavage of Dsh^{TEV} (Figures 2B, 2F, and 2J), immediately after (Figures 2C, 2G, and 2K), and 1 hr after (Figures 2D, 2H, and 2L) and compared it with complete removal of *dsh* (Figures 2A, 2E, and 2I). Immunolabelling confirmed a normal Dsh asymmetric profile prior to disruption, and its absence from the cell junctions immediately after and 1 hr after (Figures S2P and S2R). Notably, total cellular levels or junctional levels of Fz, Fmi, or Stbm were not significantly altered (Figures S2B–S2D, S2N, S2P, and S2R). However, Fz polarity was significantly reduced immediately after Dsh disruption and continued to decrease thereafter, reaching levels similar to those in *dsh*-null tissue (Figure 2M). This was accompanied by a significant reduction in the stable amount of Fz, quantified using FRAP of Fz-EGFP (Strutt et al., 2016) (Figure 2N). To measure immediate effects on Fz turnover we also used a Fz "fluorescent timer" construct fused to superfolder GFP (sfGFP) and mKate chromophores, which discriminates between "newer" (sfGFP) and "older" (mKate) pools of Fz protein, because of the different maturation rates (Barry et al., 2016). Live imaging showed an increase in sfGFP and a decrease in mKate fluorescence at junctions upon Dsh deletion, confirming an increased Fz turnover (Figures 2S and 2T).

Fmi and Stbm responded more slowly to the absence of Dsh, as their polarization and stability were unchanged immediately after disruption (Figures 2O–2R; Table S1). However, 1 hr after Dsh disruption, polarity readouts for both proteins were significantly reduced (Figures 2O, 2Q; Table S1). Although Fmi stability levels were also significantly reduced 1 hr after knockdown (Figure 2P), the same was not observed for Stbm (Figure 2R). The changes in Fmi and Fz polarity were also recapitulated using Rpn10-HA-vhhGFP and Tom70-HA-vhhGFP (Figures S2F and S2G; Table S1). Imaging of Fmi at increased resolution immediately after knockdown showed an increased sparseness of Fmi containing puncta, suggesting a loosening of the complex (Figures S2H and S2I). Our findings reveal a hierarchical relationship between the transmembrane proteins, where Fz behavior is more tightly coupled to the presence of Dsh than that of Fmi and Stbm.

Pk Translocates to the Cytoplasm upon Dsh Disruption, Independently of Dynamin Function

Pk and Dgo showed the expected junctional asymmetric localization prior to disruption of Dsh (Figures 3A, 3D, S2N, and S3A). After Dsh disruption, Dgo remained associated with the cell membrane (Figure S2O). This phenotype is different from that observed in *dsh*-null clones, where the Dgo levels at the cell membrane are strongly reduced (Figures S2L and S2M).

Strikingly, immediately after Dsh disruption, Pk moves to the cytoplasm (Figures 3B, 3E, 3H, and S3B). This Pk translocation

(F and G) Twenty-eight hr APF wing epithelium heterozygous for *dsh-EGFP* and *hs-Tom70-HA-vhhGFP* in a dsh^{V26}-null background. (F) In the absence of heat shock, Dsh-EGFP (green, F′) and Fmi (red, F″) are asymmetrically localized, and there is no detection of Tom70-HA-vhhGFP by immunolabelling (blue, F‴). (G) After expression of Tom70-HA-vhhGFP by a 2 hr heat shock at 38°C, Dsh-EGFP disappears from the cell membrane and is seen in punctate cytoplasmic spots (yellow arrows, G′), while asymmetric Fmi labeling is maintained at the cell membrane (G″). Tom70-HA-vhhGFP signal (blue) is strongly detected by immunolabelling and is restricted to the cytoplasmic region of the cells (G‴).
Insets in (D′), (F‴) and (G′) are increased intensity regions. See also Figure S1.

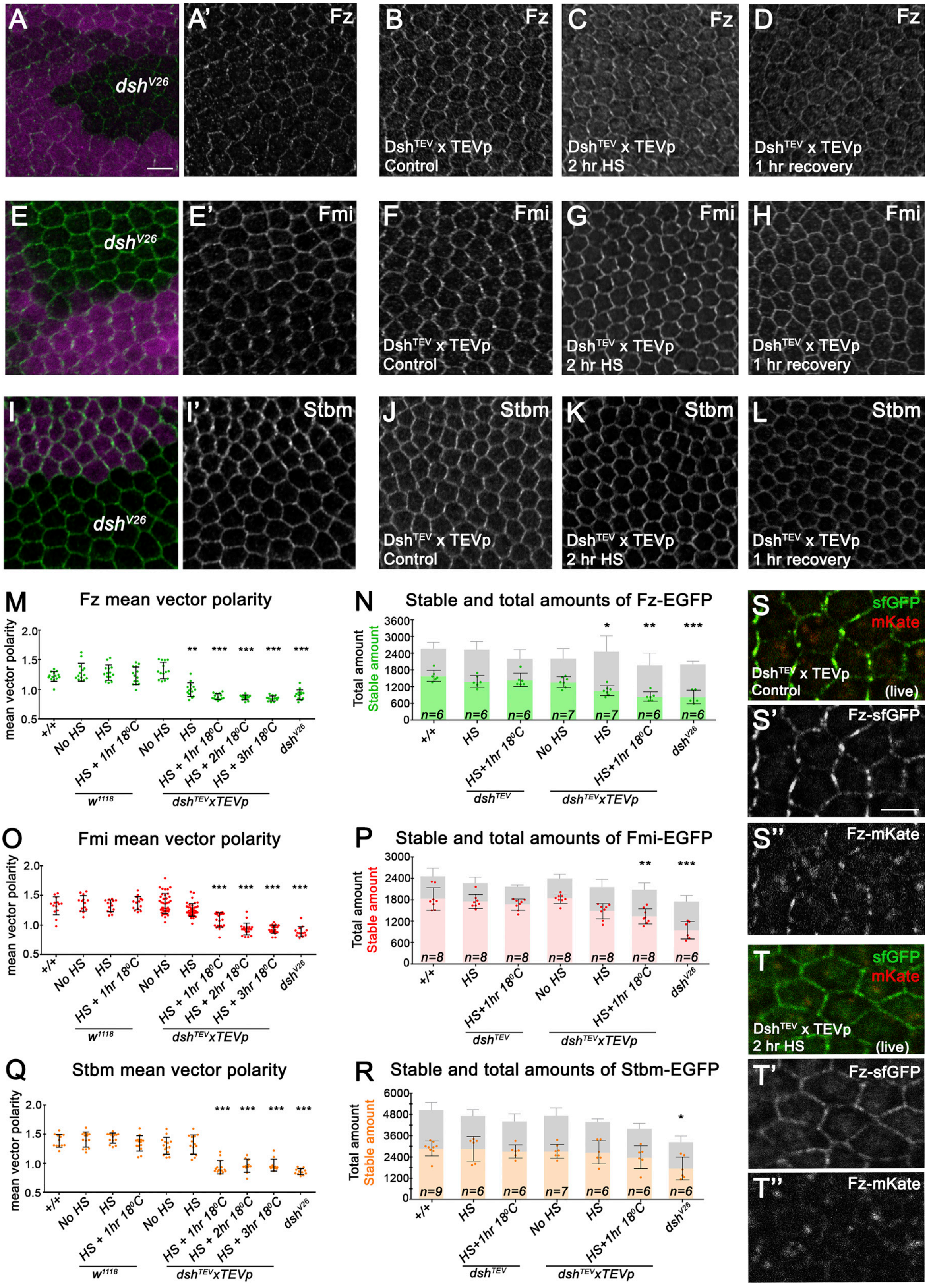

(legend on next page)

is dynamic, recovering 1 hr after Dsh disruption (Figure 3C), when a spotty symmetric membrane association can be detected, similar to that detected in *dsh*-null tissue (Figures S2K and S2U). We hypothesize that the rapid recovery of Pk localization to junctions may be because Pk contains a prenylation motif that allows it to associate with membranes independently of core protein complexes. Overall cellular levels of Pk did not change significantly (Figures S2E).

We then asked if Pk translocation is mechanistically dependent on endocytosis, as Pk is known to destabilize Fz-EGFP by endocytic mechanisms (Warrington et al., 2017). Interestingly, blocking endocytosis alone using shi^{ts1} at 29°C partially disrupts localization of Pk, but not the other core proteins, although Pk still largely maintains its asymmetry at junctions (Figures S3K–S3O). Induction of Dsh acute knockdown concomitantly with blocking of endocytosis in the pupal wing does not prevent translocation of Pk to the cytoplasm, suggesting an endocytosis-independent mechanism (Figures 3F, 3G, S3C, and S3D). Moreover, cytoplasmically relocalized Pk does not co-localize with the endosome marker Rab5 or with the nuclear marker DAPI (Figures S3E and S3F).

Pk Translocation into the Cytoplasm Produces a Propagating Wave of Destabilization of Core Protein Complexes

To understand how Dsh affects Pk junctional localization, we used mosaic analysis to determine if the effect was cell autonomous or non-autonomous. We generated clones of Dsh-EGFP expression in a background uniformly heterozygous for the *hs-Tom70-HA-vhhGFP* transgene (Figures 4A and 4B). Even though endogenous Dsh is present in addition to Dsh-EGFP, Pk translocation to the cytoplasm still occurs after heat shock-induced expression of Tom70-HA-vhhGFP (Figure 4B″; note that endogenous Dsh levels do not change; see Figure S4A). Notably, targeting of Dsh-EGFP with Tom70-HA-vhhGFP resulted in a dominant and cell-non-autonomous effect on Pk junctional localization, such that Pk also translocates to the cytoplasm in adjacent tissue lacking Dsh-GFP (Figure 4B″). The translocation of Pk into the cytoplasm propagates distally (but not proximally) away from the Dsh-EGFP tissue (Figures 4B–4D), while junctional levels of Pk in cells proximal to Dsh-EGFP knockdown tissue are similar to wild-type levels (Figure S4B). Importantly, the distal propagation of Pk release from complexes supports a model in which Dsh localized at the distal edge of one cell non-autonomously promotes Pk localization in intercellular complexes at the proximal edge of the juxtaposed neighboring cell (see Figure 4F).

To further corroborate the ability of Dsh to cell-non-autonomously influence Pk even in the absence of rapid disruption, we carried out FRAP on boundaries of null mutant *dsh* clones. We found that EGFP-Pk on cell junctions inside Dsh expressing cells that neighbor *dsh*-mutant cells had significantly reduced stability, compared with EGFP-Pk on cell junctions inside *dsh*-mutant cells that neighbor cells with Dsh activity (Figures S4C–S4I). However, Pk did not translocate into the cytoplasm, and only mild effects on junctional levels of Pk were observed around *dsh* clones (Figure S4J). Taken together, we conclude that a Dsh-dependent signal passes across cell junctions to non-autonomously maintain Pk stability and localization in core protein intercellular complexes.

Notably, the distal propagation of cytoplasmic Pk release results in destabilization of Dsh-EGFP in core protein complexes in a Dynamin-dependent manner (Figure 4E). This is consistent with our previous observation that ectopic induction of Pk expression results in cell-autonomous destabilization of Fz-Dsh complexes in a process requiring Dynamin activity (Warrington et al., 2017).

Finally we asked whether the effect of Dsh disruption on Pk localization is an indirect consequence of changes in the cell cytoskeleton that might result from the removal of Dsh. We examined the distribution of F-actin, β-tubulin, and E-cadherin immediately after Dsh disruption (Figures S4K–S4M). No changes were evident in the distribution or levels of these molecules. We also attempted to recapitulate the release of Pk from cell junctions by perturbing the actin and tubulin cytoskeletons. Neither pharmacological reduction of F-actin with latrunculin A nor genetic disruption of microtubule density via KLP10A and Katanin co-expression altered Pk membrane association (Figures S4N–S4S).

Overall we have shown that Dsh positively regulates Pk localization in core protein intercellular complexes in a cell-non-autonomous manner. Moreover, Pk that becomes relocalized to the cytoplasm is capable of destabilizing Dsh in a Dynamin-dependent

Figure 2. Dishevelled Activity Maintains the Asymmetry and Stability of the Transmembrane Core Planar Polarity Proteins

(A, E, and I) Twenty-eight hr APF wing epithelia containing clones of dsh^{V26}-null mutant tissue (loss of RFP, magenta), immunolabelled for endogenous Fz (A), Fmi (E), and Stbm (I). Scale bar, 5 μm and the same hereafter.

(B–L) Twenty-eight hr APF wing epithelia showing localization of Fz (B–D), Fmi (F–H), or Stbm (J–L) before cleavage of Dsh^{TEV} (B, F, and J), immediately after (C, G, and K), and 1 hr after (D, H, and L).

(M, O, and Q) Fz (M), Fmi (O), and Stbm (Q) polarity measurements before and after cleavage of Dsh^{TEV} and in dsh^{V26} and wild-type tissue. Error bars are SD; each data point represents one wing.

(N, P, and R) FRAP experiments, showing stable amounts and total amounts of Fz-EGFP (N), Fmi-EGFP (P), or Stbm-EGFP (R) before and after cleavage of Dsh^{TEV}. Error bars are SD; n, number of wings. For (M–R), ANOVA with Tukey-Kramer multiple-comparison test was used to compare all samples. Shown are comparisons of each experimental genotype to no heat-shock conditions and +/+ to dsh^{V26} clone tissue. *$p \leq 0.05$, **$p \leq 0.01$, and ***$p \leq 0.001$. See also Table S2.

(S and T) Twenty-eight hr APF wing epithelium showing localization of Dsh^{TEV} in a dsh^{V26}-null background, before and after cleavage by TEVp in the presence of the Fz "fluorescent timer" protein (Fz-mKate-sfGFP). (S) In the absence of heat-shock both "new" Fz (sfGFP) (S′) and "old" Fz (mKate) (S″) are asymmetrically localized at the junctions. (T) After a 2 hr heat shock, sfGFP is still detectable at the cell membrane and cytoplasm (T′), while mKate is reduced at the membrane (T″).

See also Figure S2 and Tables S2 and S3.

OPEN ACCESS
CellPress

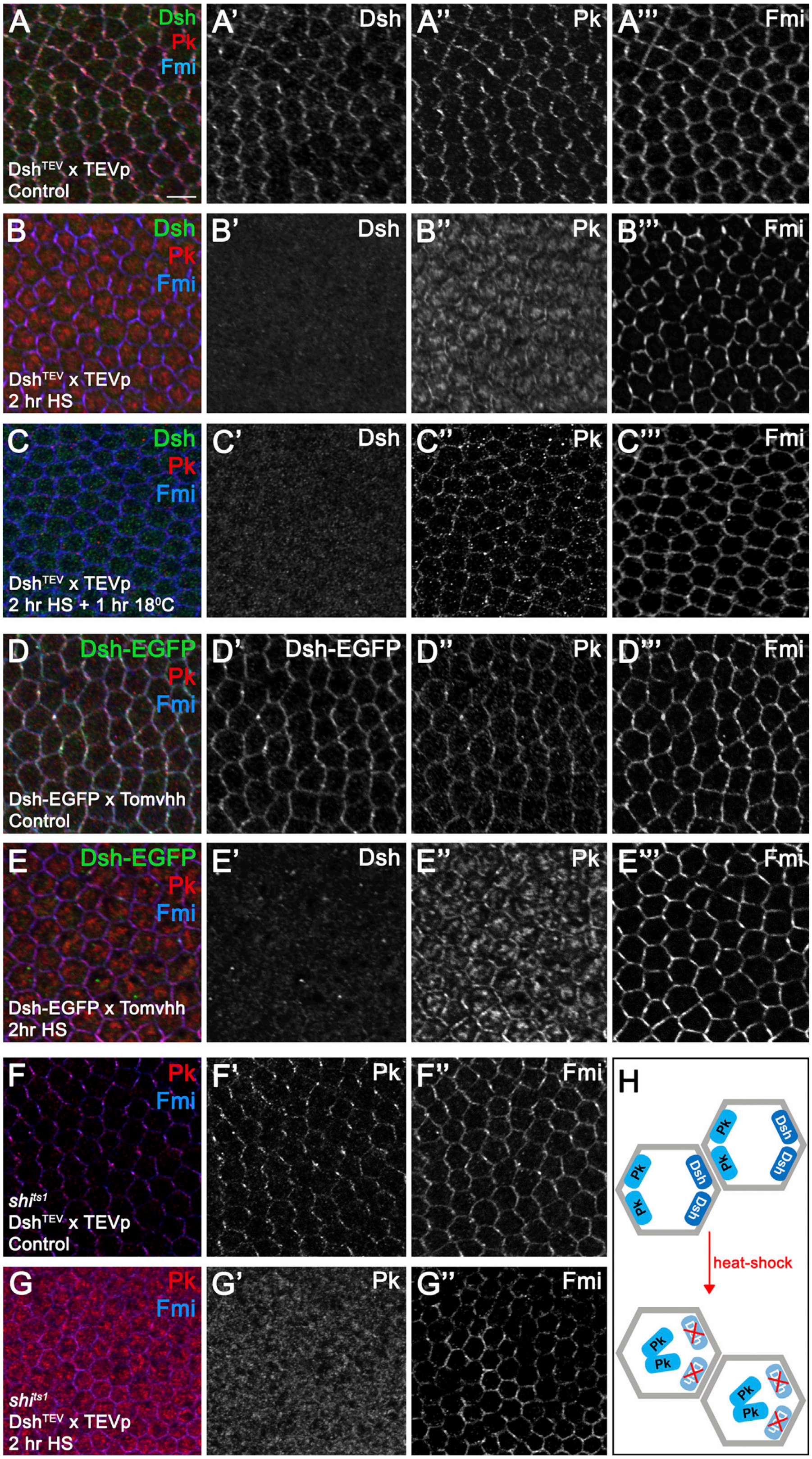

(legend on next page)

manner, leading to a wave of destabilization of core protein complexes across the tissue (Figure 4F).

DISCUSSION

Defining the roles of individual components in signaling networks can be a significant challenge. This is particularly so when the network is not a simple linear pathway, if components play more than one role in the cell (pleiotropy), and if there is ''adaptation'' such that over time, the pathway adjusts to the effects of experimental manipulations. However, in many cases, these difficulties can be bypassed through methods that rapidly alter protein activities (Hoeller et al., 2014; Harmansa et al., 2017).

Consistent with this, we have recently shown that spatiotemporal activation of gene expression is an effective tool for dissecting feedback interactions during planar polarity patterning in the *Drosophila* wing (Warrington et al., 2017). In this work, we now use methods for rapidly disrupting protein function to probe the role of the Dsh protein in planar polarity.

Our main finding is that Dsh regulates Pk membrane association in core planar polarity complexes, acting cell-non-autonomously to prevent its relocalization to the cytoplasm. Notably, this role for Dsh is specifically revealed when Dsh is rapidly depleted from core protein complexes but not in the simple *dsh* loss-of-function situation, when instead a largely mobile fraction of Pk is seen associated with cell junctions. We speculate that a Dsh-dependent signal normally passes between cells via the core protein complexes to maintain Pk recruitment. When this signal is disrupted, Pk rapidly leaves the junctions. However, in the long-term absence of Dsh, Pk can return to cell junctions, where we speculate it weakly associates with cell membranes by virtue of being prenylated (Strutt et al., 2013b).

What might be the nature of the intercellular signal from Dsh to Pk? We suggest it passes via the Fmi homodimers that form between cells, as numerous lines of evidence indicate these are essential for cell-cell signaling in planar polarity (reviewed in Strutt and Strutt, 2009). A simple possibility is that Dsh binding to Fz induces a conformational change in the complex that passes via the Fmi homodimers to alter the conformation of bound Stbm, thus creating a Pk binding site. Such molecular signaling events mediated by allostery are common features of ligand-receptor interactions (Nussinov et al., 2013). In support of the model that the Dsh signal is transduced via a change in Fz behavior, we note that following Dsh disruption, Fz distribution and stability is altered faster than those of Fmi and Stbm (Figure 2).

A related mechanism is suggested by our recent observations that the core proteins incorporate into intercellular complexes non-stoichiometrically and that all components contribute to complex stability (Strutt et al., 2016; Warrington et al., 2017). We interpret these findings as suggesting that core complex stability is dependent on a phase transition mediated by multivalent interactions between the core proteins, with Dsh playing a critical role (Warrington et al., 2017). The rapid destabilization of Fz after Dsh depletion may be a result of loss of multivalent binding interactions mediated between the different domains of Dsh, as also occurs in Wnt signalosome assembly (Gammons and Bienz, 2018), over time leading to a reduction in multivalent binding interactions between Fmi and Stbm. This would thus produce a gradual ''loosening'' of the complex that would result in release of Pk from its binding interactions with Fmi and Stbm. Some support for this model comes from our observation that super-resolution microscopy immediately after Dsh disruption shows subtle changes in the size and distribution of Fmi in junctional puncta (Figure S2I).

A striking observation is that if Dsh fails to maintain Pk recruitment in intercellular complexes, free Pk can destabilize Dsh in the same cell, leading to release of Pk in the neighboring cell and a wave of core planar polarity complex destabilization (Figures 4B and 4F). This observation both supports our previous work showing that physiological levels of Pk can effectively destabilize Fz-Dsh complexes at cell junctions (Warrington et al., 2017) and highlights the importance of sequestering Pk into ''proximal'' complexes, to prevent unregulated activity of Pk within the cell. We propose that Fmi mediates essential intercellular signals from Fz-Dsh in ''distal'' core complexes that actively maintain Pk in ''proximal'' core protein complexes. In turn, this promotes the effective segregation of distal and proximal complexes to opposite cell ends, driven in part by destabilizing feedback interactions between Pk and Dsh in the same cell.

STAR★METHODS

Detailed methods are provided in the online version of this paper and include the following:

- KEY RESOURCES TABLE
- CONTACT FOR REAGENT AND RESOURCE SHARING

Figure 3. Dishevelled Disruption Leads to the Translocation of Prickle to the Cytoplasm

(A–C) Twenty-eight hr APF wing epithelia showing Pk localization before (A), immediately after (B), or 1 hr after (C) Dsh^{TEV} cleavage, showing Pk (red), Dsh (green), and Fmi (blue). (B) Note the cytoplasmic fraction of Pk (B″) after Dsh^{TEV} cleavage, while junctional localization of Fmi is maintained (B‴). (C) One hr after Dsh^{TEV} cleavage, Pk shows spotty junctional localization (C″), negligible junctional Dsh is detected (C′), and Fmi shows reduced asymmetry (C‴). Scale bar, 5 μm.

(D and E) Twenty-eight hr APF wing epithelia expressing Dsh-EGFP in a dsh^{V26}-null background before (D) and after (E) sequestration of Dsh-EGFP using *hs-Tom70-HA-vhhGFP*. (D) In the absence of heat-shock Dsh-EGFP (D′), Pk (D″), and Fmi (D‴) localize asymmetrically. (E) Sequestration of Dsh-EGFP leads to Dsh-EGFP disappearance from the cell junctions (E′), Pk localization to the cytoplasm (E″), while Fmi maintains its asymmetry (E‴).

(F and G) Effect of cleavage of Dsh^{TEV} using *hs-TEVp* in a dsh^{1} shi^{ts1} background. (F) At 18°C, Pk (F′) and Fmi (F″) are asymmetrically localized. (G) Immediately after 2 hr heat shock at 38°C, Dsh^{TEV} cleavage still results in Pk localization to the cytoplasm (G′) even though Dynamin-dependent endocytosis should be blocked under these conditions, while Fmi remains membrane associated (G″).

(H) Schematic representation depicting the relocation of Pk after Dsh disruption.

See also Figure S3.

OPEN
ACCESS
CellPress

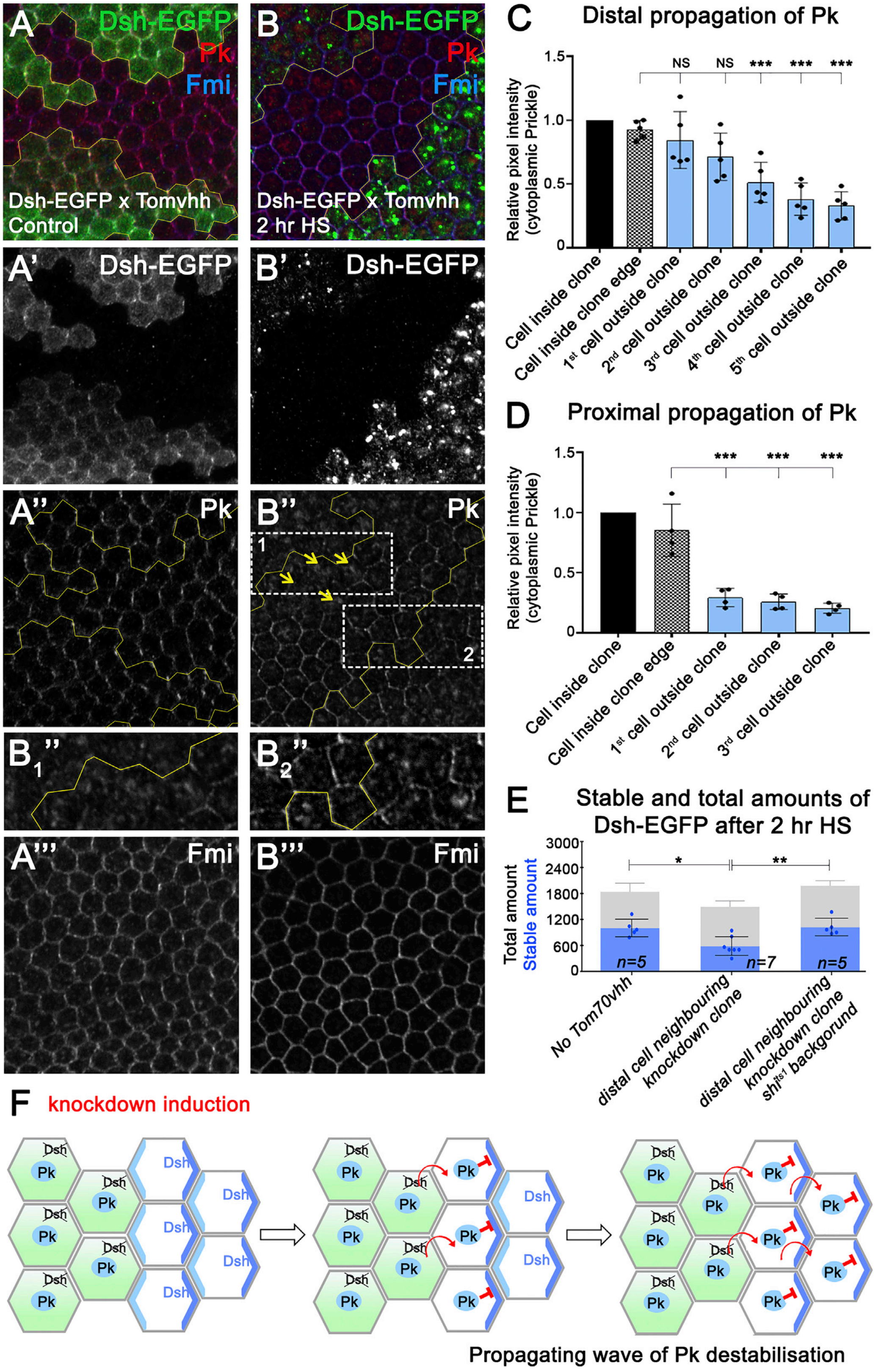

(legend on next page)

- EXPERIMENTAL MODEL AND SUBJECT DETAILS
- METHOD DETAILS
  - Molecular Biology
  - Fly genetics
  - Temperature regimes for protein activity disruption
  - Immunostaining and antibodies
  - Western blotting
  - Cytoskeleton disruption assays
  - Heat-shock induction of tools to disrupt protein activity
  - Imaging
- QUANTIFICATION AND STATISTICAL ANALYSIS
  - FRAP processing
  - Statistics
  - Polarity measurement
  - Quantification of Pk propagation
  - Quantification of Pk membrane levels

SUPPLEMENTAL INFORMATION

Supplemental Information includes four figures and three tables and can be found with this article online at https://doi.org/10.1016/j.celrep.2018.10.039.

ACKNOWLEDGMENTS

We thank Jessica Gamage for making *P[acman]-dsh-EGFP*, Reinhard Schuh, Christian Dahmann, the Bloomington Drosophila Stock Center, and NIG for fly stocks, the Developmental Studies Hybridoma Bank for antibodies, Markus Affolter and Addgene for plasmids, and BestGene and Genetivision for generating transformants. We thank the fly room staff for excellent technical support and members of the Strutt lab for comments on the manuscript. This work was funded by a Wellcome Senior Fellowship (grant 100986/Z/13/Z) awarded to D.S. Imaging was performed in the Wolfson Light Microscopy Facility.

AUTHOR CONTRIBUTIONS

D.S. conceived the project, generated tools and reagents, designed experiments, and wrote the manuscript. M.R. designed and conducted experiments, analyzed data, and wrote the manuscript. S.W. designed and conducted experiments, analyzed data, and edited the manuscript.

DECLARATION OF INTERESTS

The authors declare no competing interests.

Received: June 13, 2018
Revised: September 17, 2018
Accepted: October 9, 2018
Published: November 6, 2018

REFERENCES

Barry, J.D., Donà, E., Gilmour, D., and Huber, W. (2016). TimerQuant: a modelling approach to tandem fluorescent timer design and data interpretation for measuring protein turnover in embryos. Development *143*, 174–179.

Bastock, R., and Strutt, D. (2007). The planar polarity pathway promotes coordinated cell migration during *Drosophila* oogenesis. Development *134*, 3055–3064.

Butler, M.T., and Wallingford, J.B. (2017). Planar cell polarity in development and disease. Nat. Rev. Mol. Cell Biol. *18*, 375–388.

Caussinus, E., Kanca, O., and Affolter, M. (2011). Fluorescent fusion protein knockout mediated by anti-GFP nanobody. Nat. Struct. Mol. Biol. *19*, 117–121.

Devenport, D. (2016). Tissue morphodynamics: translating planar polarity cues into polarized cell behaviors. Semin. Cell Dev. Biol. *55*, 99–110.

Feiguin, F., Hannus, M., Mlodzik, M., and Eaton, S. (2001). The ankyrin repeat protein Diego mediates Frizzled-dependent planar polarization. Dev. Cell *1*, 93–101.

Gammons, M., and Bienz, M. (2018). Multiprotein complexes governing Wnt signal transduction. Curr. Opin. Cell Biol. *51*, 42–49.

Goodrich, L.V., and Strutt, D. (2011). Principles of planar polarity in animal development. Development *138*, 1877–1892.

Gubb, D., Green, C., Huen, D., Coulson, D., Johnson, G., Tree, D., Collier, S., and Roote, J. (1999). The balance between isoforms of the Prickle LIM domain protein is critical for planar polarity in Drosophila imaginal discs. Genes Dev. *13*, 2315–2327.

Harder, B., Schomburg, A., Pflanz, R., Küstner, K., Gerlach, N., and Schuh, R. (2008). TEV protease-mediated cleavage in *Drosophila* as a tool to analyze protein functions in living organisms. Biotechniques *44*, 765–772.

Harmansa, S., Alborelli, I., Bieli, D., Caussinus, E., and Affolter, M. (2017). A nanobody-based toolset to investigate the role of protein localization and dispersal in *Drosophila*. eLife *6*, e22549.

Hoeller, O., Gong, D., and Weiner, O.D. (2014). How to understand and outwit adaptation. Dev. Cell *28*, 607–616.

Janse, D.M., Crosas, B., Finley, D., and Church, G.M. (2004). Localization to the proteasome is sufficient for degradation. J. Biol. Chem. *279*, 21415–21420.

Nussinov, R., Tsai, C.J., and Ma, B. (2013). The underappreciated role of allostery in the cellular network. Annu. Rev. Biophys. *42*, 169–189.

Oda, H., Uemura, T., Harada, Y., Iwai, Y., and Takeichi, M. (1994). A *Drosophila* homolog of cadherin associated with armadillo and essential for embryonic cell-cell adhesion. Dev. Biol. *165*, 716–726.

Figure 4. Cytoplasmic Translocation of Pk Results in a Propagating Wave of Destabilization of Core Protein Complexes

(A and B) Twenty-eight hr APF pupal wings carrying clones of Dsh-EGFP, in a dsh^{V26}-heterozygous background, with Tom70-HA-vhhGFP expressed in all cells (clone boundaries marked by yellow lines). (A) In the absence of heat shock, Dsh-EGFP (A′), Pk (A″), and Fmi (A‴) are all asymmetrically localized inside (and outside in the case of Pk and Fmi) Dsh-EGFP clones. (B) Immediately after heat shock, Dsh-EGFP in clones becomes punctate and is absent from the cell membranes (B′). Pk localization (B″) becomes cytoplasmic inside clones (white rectangle 1, inset B_1'') and propagates distally to cells outside of the Dsh-EGFP expressing tissue (yellow arrows), where Dsh is not targeted by vhhGFP expression. Pk localization remains junctional proximal to Dsh-EGFP clones (white rectangle 2, inset B_2''). Fmi remains associated with the cell junctions (B‴). Scale bar, 5 μm.

(C and D) Quantification of cytoplasmic Pk inside Dsh-EGFP clone knockdown tissue and its propagation through cells distal (C) or proximal (D) to a clone. Error bars are SD; each data point represents one wing. ANOVA with Dunnett's multiple comparison test was used to compare the first cell inside the clone edge (column 2) with all other samples; ***$p \leq 0.001$.

(E) Dsh-EGFP stable amounts determined by FRAP analysis, showing control cells (left column), cells lacking Tom70-vhhGFP that are immediately distal to cells in which Dsh-EGFP has been sequestered by Tom70-HA-vhhGFP (middle column, heterozygous for Dsh-EGFP and Tom70-vhhGFP), and similar cells that are also hemizygous for shi^{ts1} and thus should be inhibited for Dynamin-dependent endocytosis (right column). Error bars are SD; n, number of wings. ANOVA with Tukey-Kramer multiple-comparison test was used to compare all genotypes; *$p \leq 0.05$ and **$p \leq 0.01$. See also Table S2.

(F) Schematic representation of Pk release upon Dsh disruption in clonal tissue and the further destabilization of Dsh by cytoplasmic Pk creating a propagating wave of polarity destabilization.

See also Figure S4 and Table S2 and S3.

Pauli, A., Althoff, F., Oliveira, R.A., Heidmann, S., Schuldiner, O., Lehner, C.F., Dickson, B.J., and Nasmyth, K. (2008). Cell-type-specific TEV protease cleavage reveals cohesin functions in *Drosophila* neurons. Dev. Cell *14*, 239–251.

Perrimon, N., and Mahowald, A.P. (1987). Multiple functions of segment polarity genes in *Drosophila*. Dev. Biol. *119*, 587–600.

Rawls, A.S., and Wolff, T. (2003). Strabismus requires Flamingo and Prickle function to regulate tissue polarity in the *Drosophila* eye. Development *130*, 1877–1887.

Robinson, M.S., Sahlender, D.A., and Foster, S.D. (2010). Rapid inactivation of proteins by rapamycin-induced rerouting to mitochondria. Dev. Cell *18*, 324–331.

Strutt, H., and Strutt, D. (2008). Differential stability of flamingo protein complexes underlies the establishment of planar polarity. Curr. Biol. *18*, 1555–1564.

Strutt, H., and Strutt, D. (2009). Asymmetric localisation of planar polarity proteins: Mechanisms and consequences. Semin. Cell Dev. Biol. *20*, 957–963.

Strutt, H., Price, M.A., and Strutt, D. (2006). Planar polarity is positively regulated by casein kinase Iepsilon in *Drosophila*. Curr. Biol. *16*, 1329–1336.

Strutt, H., Warrington, S.J., and Strutt, D. (2011). Dynamics of core planar polarity protein turnover and stable assembly into discrete membrane subdomains. Dev. Cell *20*, 511–525.

Strutt, H., Searle, E., Thomas-Macarthur, V., Brookfield, R., and Strutt, D. (2013a). A Cul-3-BTB ubiquitylation pathway regulates junctional levels and asymmetry of core planar polarity proteins. Development *140*, 1693–1702.

Strutt, H., Thomas-MacArthur, V., and Strutt, D. (2013b). Strabismus promotes recruitment and degradation of farnesylated prickle in *Drosophila melanogaster* planar polarity specification. PLoS Genet. *9*, e1003654.

Strutt, H., Gamage, J., and Strutt, D. (2016). Robust asymmetric localization of planar polarity proteins is associated with organization into signalosome-like domains of variable stoichiometry. Cell Rep. *17*, 2660–2671.

Usui, T., Shima, Y., Shimada, Y., Hirano, S., Burgess, R.W., Schwarz, T.L., Takeichi, M., and Uemura, T. (1999). Flamingo, a seven-pass transmembrane cadherin, regulates planar cell polarity under the control of Frizzled. Cell *98*, 585–595.

Wang, S., Zhao, Y., Leiby, M., and Zhu, J. (2009). A new positive/negative selection scheme for precise BAC recombineering. Mol. Biotechnol. *42*, 110–116.

Warrington, S.J., Strutt, H., and Strutt, D. (2013). The Frizzled-dependent planar polarity pathway locally promotes E-cadherin turnover via recruitment of RhoGEF2. Development *140*, 1045–1054.

Warrington, S.J., Strutt, H., Fisher, K.H., and Strutt, D. (2017). A dual function for Prickle in regulating Frizzled stability during feedback-dependent amplification of planar polarity. Curr. Biol. *27*, 2784–2797.e3.

Widmann, T.J., and Dahmann, C. (2009). Dpp signaling promotes the cuboidal-to-columnar shape transition of *Drosophila* wing disc epithelia by regulating Rho1. J. Cell Sci. *122*, 1362–1373.

Wolff, T., and Rubin, G.M. (1998). *strabismus*, a novel gene that regulates tissue polarity and cell fate decisions in *Drosophila*. Development *125*, 1149–1159.

STAR★METHODS

KEY RESOURCES TABLE

REAGENT or RESOURCE	SOURCE	IDENTIFIER
Antibodies		
Rabbit anti-GFP, affinity purified	Abcam	cat#ab6556; RRID: AB_305564
Mouse monoclonal anti-β-actin AC-40	Sigma-Aldrich	cat#A4700; RRID: AB_476730
Rat monoclonal anti-HA 3F10	Sigma-Aldrich	cat#12158167001; RRID: AB_390918
Mouse anti-β-tubulin E7 s	DSHB	RRID: AB_2315513
Mouse monoclonal anti-Fmi #74	DSHB (Usui et al., 1999)	RRID: AB_2619583
Rat anti-Pk, affinity purified	David Strutt (Strutt et al., 2013a)	N/A
Rabbit anti-Dsh, affinity purified (western blotting)	David Strutt (Strutt et al., 2006)	N/A
Rat anti-Dsh (immunolabelling)	David Strutt (Strutt et al., 2006)	N/A
Rabbit anti-Stbm (western blotting)	Tanya Wolff (Rawls and Wolff, 2003)	N/A
Rabbit anti-Stbm (immunolabelling)	David Strutt (Warrington et al., 2013)	N/A
Rat anti-Stbm (immunolabelling)	David Strutt (Strutt and Strutt, 2008)	N/A
Rabbit anti-Fz, affinity purified	David Strutt (Bastock and Strutt, 2007)	N/A
Rat anti-E-Cad	DSHB (Oda et al., 1994)	N/A
Rabbit anti-Rab5	Abcam	cat#ab13253; RRID: AB_2569809
Chemicals, Peptides, and Recombinant Proteins		
16% paraformaldehyde solution (methanol free)	Agar Scientific	cat#R1026
Phalloidin Alexa-568	ThermoFisher	cat#A12380
NucBlue (DAPI)	ThermoFisher	cat#R37605
Triton X-100	VWR	cat#28817.295; CAS: 9002-93-1
Tween-20	VWR	cat#437082Q; CAS: 9005-64-5
Methyl cellulose	Sigma-Aldrich	cat#M0387; CAS: 9004-67-5
Latrunculin A	ThermoFisher	cat#L12370; CAS: 76343-93-6
DMSO	Sigma-Aldrich	cat#D9170; CAS: 67-68-5
Schneider's medium	ThermoFisher	cat#21720-024
Glycerol	VWR	cat#284546F; CAS: 56-81-5
DABCO	Fluka	cat#33480; CAS: 280-57-9
Normal Goat Serum	Jackson Labs	cat#005-000-121
Halocarbon 700 Oil	Halocarbon Products Corp.	CAS: 9002-83-9
Experimental Models: Organisms/Strains		
stbm^{6}	Tanya Wolff (Wolff and Rubin, 1998)	FlyBase: FBal0062423
dsh^{V26}	Norbert Perrimon (Perrimon and Mahowald, 1987)	FlyBase: FBal0003140
dsh^{1}	Bloomington Drosophila Stock Center	FlyBase: FBal0003138; RRID: BDSC_5298
dgo^{380}	Suzanne Eaton (Feiguin et al., 2001)	FlyBase: FBal0141190
pk$^{pk\text{-}sple13}$	David Gubb (Gubb et al., 1999)	N/A
shi^{ts1}	Bloomington Drosophila Stock Center	FlyBase: FBal0015610; RRID: BDSC_7068
fz-EGFP	David Strutt (Strutt et al., 2016)	N/A
EGFP-pk	David Strutt (Strutt et al., 2016)	N/A
fmi-EGFP	David Strutt (Strutt et al., 2016)	N/A
P[acman]-dsh-EGFP	This paper	N/A
P[acman]-stbm-EGFP	David Strutt (Strutt et al., 2016)	N/A
P[acman]-EGFP-dgo	David Strutt (Strutt et al., 2016)	N/A
P[acman]-dsh^{3xTEV}	This paper	N/A
P[CaSpeR]-hs-Tom70-HA-vhhGFP	This paper	N/A

(Continued on next page)

Continued

REAGENT or RESOURCE	SOURCE	IDENTIFIER
P[CaSpeR]-hs-Rpn10-HA-vhhGFP	This paper	N/A
ActP-FRT-polyA-FRT-fz-mKate-sfGFP	This paper	N/A
P[CaSpeR]-hs-TEVp	Reinhard Schuh (Harder et al., 2008)	N/A
pUAS-TEVp	Reinhard Schuh (Harder et al., 2008)	N/A
pUAS-KLP-10A, pUAS-Katanin-60	Christian Dahmann (Widmann and Dahmann, 2009)	N/A
pUAS-dshNIG$^{18361R-2}$	National Institute of Genetics	Stock ID: 18361R-2 NIG078563.2
pUAS-dsh^{WIZ}	David Strutt (Bastock and Strutt, 2007)	N/A
hs-FLP	Bloomington Drosophila Stock Center	FlyBase: FBti0002044; RRID: BDSC_6
Ubx-FLP	Bloomington Drosophila Stock Center	FlyBase: FBti0150334; RRID: BDSC_42718
Actin-GAL4	Bloomington Drosophila Stock Center	FlyBase: FBti0012293; RRID: BDSC_4414
tubulin-GAL80^{ts}	Bloomington Drosophila Stock Center	FlyBase: FBti0027796; RRID: BDSC_7019
Ubi-mRFP-nls	Bloomington Drosophila Stock Center	FlyBase: FBti0129786; RRID: BDSC_30852
Software and Algorithms		
NIS Elements AR version 4.60	Nikon	N/A
Image Lab version 4.1	BioRad Laboratories	N/A
ImageJ version 2.0.0	https://fiji.sc	N/A
MATLAB_R2014b	Mathworks	N/A
GraphPad Prism version 7.0c	GraphPad Software, Inc.	N/A
G*Power version 3.1	http://www.gpower.hhu.de	N/A
Tissue Analyzer	https://grr.gred-clermont.fr/labmirouse/software/WebPA/	N/A
Polarity measurement scripts (MATLAB)	David Strutt (Strutt et al., 2016)	N/A

CONTACT FOR REAGENT AND RESOURCE SHARING

Further information and requests for resources and reagents should be directed to and will be fulfilled by the Lead Contact, David Strutt (d.strutt@sheffield.ac.uk).

EXPERIMENTAL MODEL AND SUBJECT DETAILS

Drosophila melanogaster flies were grown on standard cornmeal/agar/molasses media at 18°C or 25°C, unless otherwise described.

METHOD DETAILS

Molecular Biology

P[acman]-dsh-EGFP and *P[acman]-dsh^{3xTEV}* were generated from *P[acman]-dsh* (Strutt et al., 2016) via recombineering (Wang et al., 2009). *P[acman]-dsh-EGFP* is a fusion of EGFP to the C terminus of Dsh, made as previously described (Strutt et al., 2016), and for *P[acman]-dsh^{TEV}* three TEVp sites were inserted between the codons encoding amino acids 379-380. *ActP-FRT-polyA-FRT-fz-sfGFP-mKate*, is a fusion of sfGFP and mKate to the C terminus of Fz. *P[CaSpeR]-hs-Tom70-HA-vhhGFP* and *P[CaSpeR]-hs-Rpn10-HA-vhhGFP* are fusions of amino acids 1-50 of *Drosophila Tom70* or the full-length *Drosophila Rpn10* coding sequence respectively to the *vhhGFP* coding sequence, separated by a linker containing a single HA-tag sequence. Full sequences and cloning details are available upon request.

Fly genetics

Fly strains are described in the Key Resources Table. Mutant alleles are described in FlyBase. *stbm^{6}*, *dsh^{V26} pk$^{pk-sple13}$* and *dgo^{380}* are null alleles and unable to give rise to functional proteins; *dsh^{1}* contains a missense mutation in the DEP domain which has been reported to be a strong mutation for planar polarity activity. *shi^{ts1}* is a thermosensitive Dynamin mutation which allows normal endocytosis to occur at the permissive temperature (18°C) and is less active at the restrictive temperature (29°C).

P[acman] constructs were integrated into the genome via ΦC31-mediated recombination into the *attP40* landing site. For the acute knockdown of Dsh we used genomic rescue constructs *P[acman]-dsh^{3xTEV}* and *P[acman]-dsh-EGFP*, and genetically encoded

effector transgenes expressed under heat-shock promoter control *pCaSpeR-hs-TEVp* (Harder et al., 2008), *pCaSpeR-hs-Tom70-HA-vhhGFP* and *pCaSpeR-hs-Rpn10-HA-vhhGFP*. Two *UAS-dsh-RNAi* transgenes (*dsh*$^{NIG18361R-2}$ [RNAi1] and *dsh*^{WIZ} [RNAi2] (Bastock and Strutt, 2007)) and a *UAS-TEVp* (Harder et al., 2008) line were conditionally expressed using *Act-GAL4, tub-GAL80*^{ts}. Mitotic clones were produced using the FLP/FRT system with *Ubx-FLP* or *hs-FLP*. The fluorescent timer transgene *ActP-FRT-polyA-FRT-fz-sfGFP-mKate* was used to study Fz turnover. FRAP experiments used knock-ins of *EGFP-pk*, *fmi-EGFP* and *fz-EGFP* or genomic rescue constructs for *P[acman]-stbm-EGFP* and *P[acman]-EGFP-dgo,* in *stbm*^{6} or *dgo*^{380} mutant backgrounds respectively (Strutt et al., 2016). *UAS-KLP10A* and *UAS-Katanin-60* were used to depolymerize and sever microtubules (Widmann and Dahmann, 2009). Transgenics were generated by Genetivision and BestGene.

Genotypes for experiments were:

Figure 1
(C-D) *dsh*^{V26}*/Y; P[acman]-dsh*^{3xTEV}*/P[CaSpeR]-hs-TEVp*
(F-G) *dsh*^{V26}*/Y; P[acman]-dsh-EGFP/P[CaSpeR]-hs-Tom70-HA-vhhGFP*
Figure 2
(A,E,I) *dsh*^{V26} *FRT19A/ubi-mRFP-nls FRT19A; Ubx-FLP/+*
(B-D, F-H and J-L) *dsh*^{V26}*/Y; P[acman]-dsh*^{3xTEV}*/P[CaSpeR]-hs-TEVp*
(M, O and Q) w^{1118}
dsh^{V26}*/Y; P[acman]-dsh*^{3xTEV}*/P[CaSpeR]-hs-TEVp*
dsh^{V26} *FRT19A/ ubi-mRFP-nls FRT19A; Ubx-FLP/+*
(N) *w*^{1118}*; fz-EGFP/+*
dsh^{V26}*/Y; P[acman]-dsh*^{3xTEV}*/+; fz-EGFP/+*
dsh^{V26}*/Y; P[acman]-dsh*^{3xTEV}*/P[CaSpeR]-hs-TEVp; fz-EGFP/+*
dsh^{V26} *FRT19A/ ubi-mRFP-nls FRT19A; Ubx-FLP/+; fz-EGFP/+*
(P) *w*^{1118}*; fmi-EGFP/+*
dsh^{V26}*/Y; P[acman]-dsh*^{3xTEV}*/fmi-EGFP*
dsh^{V26}*/Y; P[acman]-dsh*^{3xTEV}*/fmi-EGFP; P[CaSpeR]-hs-TEVp/+*
dsh^{V26} *FRT19A/ubi-mRFP-nls FRT19A; Ubx-FLP/fmi-EGFP*
(R) *w*^{1118}*; P[acman]-stbm-EGFP stbm*^{6}*/+*
dsh^{V26}*/Y; P[acman]-dsh*^{3xTEV}*/P[acman]-stbm-EGFP stbm*^{6}
dsh^{V26}*/Y; P[acman]-dsh*^{3xTEV}*/P[acman]-stbm-EGFP stbm*^{6}*; P[CaSpeR]-hs-TEVp/+*
dsh^{V26} *FRT19A/ubi-mRFP-nls FRT19A; Ubx-FLP/P[acman]-stbm-EGFP stbm*^{6}
(S-T) *Ubx-FLP, dsh*^{V26}*/Y; P[acman]-dsh*^{3xTEV}*/P[CaSpeR]-hs-TEVp; ActP-FRT-polyA-FRT-fz-sfGFP-mKate/+*
Figure 3
(A-C) *dsh*^{V26}*/Y; P[acman]-dsh*^{3xTEV}*/P[CaSpeR]-hs-TEVp*
(D-E) *dsh*^{V26}*/Y; P[acman]-dsh-EGFP/P[CaSpeR]-hs-Tom70-HA-vhhGFP*
(F-G) *dsh*^{1}*, shi*^{ts1}*, hs-FLP/Y; P[acman]-dsh*^{3xTEV}*/P[CaSpeR]-hs-TEVp*
Figure 4
(A-D) *UbxFLP, dsh*^{V26}*/w*^{1118}*; P[acman]-dsh-EGFP FRT40/FRT40; P[CaSpeR]-hs-Tom70-HA-vhhGFP/+*
(E) *UbxFLP, dsh*^{V26}*/Y; P[acman]-dsh-EGFP FRT40/P[CaSpeR]-hs-Tom70-HA-vhhGFP FRT40*
dsh^{1}*, shi*^{ts1}*, hs-FLP/Y; P[acman]-dsh-EGFP FRT40/hs-Tom70-HA-vhhGFP4 FRT40*
Figure S1
(C-D) *dsh*^{V26}*/Y; P[acman]-dsh-EGFP/P[CaSpeR]-hs-Rpn10-HA-vhhGFP*
(E-F) *dsh*^{V26}*/Y; P[acman]-dsh*^{3xTEV}*/P[CaSpeR]-hs-TEVp*
dsh^{V26}*/Y; P[acman]-dsh-EGFP/P[CaSpeR]-hs-Tom70-HA-vhhGFP*
dsh^{V26}*/Y; P[acman]-dsh-EGFP/P[CaSpeR]-hs-Rpn10-HA-vhhGFP*
(G-H) *w*^{1118}*/Y; Act-GAL4, tub-GAL80*^{ts}*/+; UAS-dsh*$^{NIG18361R-2}$*/+*
(I-J) *w*^{1118}*/Y; Act-GAL4, tub-GAL80*^{ts}*/+; UAS-dsh*^{WIZ}*/+*
(K-L) *dsh*^{V26}*/Y; P[acman]-dsh*^{3xTEV}*/pUAS-TEVp*
Figure S2
(A-E) *w*^{1118}*/Y; +/P[CaSpeR]-hs-TEVp*
dsh^{V26}*/Y; P[acman]-dsh*^{3xTEV}*/P[CaSpeR]-hs-TEVp*
(F-G) *w*^{1118}
dsh^{V26}*/Y; P[acman]-dsh-EGFP/+*
dsh^{V26}*/Y; P[acman]-dsh-EGFP/P[CaSpeR]-hs-Tom70-HA-vhhGFP*
dsh^{V26}*/Y; P[acman]-dsh-EGFP/P[CaSpeR]-hs-Rpn10-HA-vhhGFP*
dsh^{V26} *FRT19A/ubi-mRFP-nls FRT19A; Ubx-FLP/+*
(H-I) *dsh*^{V26}*/Y; P[acman]-dsh*^{3xTEV}*/P[CaSpeR]-hs-TEVp*
(J-K) *dsh*^{V26} *FRT19A/ubi-mRFP-nls FRT19A; Ubx-FLP/+*

OPEN ACCESS
CellPress

(L-M) *dsh*^{V26} *FRT19A/ubi-mRFP-nls FRT19A; Ubx-FLP/P[acman]-EGFP-dgo dgo*^{380}
(N-O) *dsh*^{V26}*/Y; P[acman]-dsh*^{3xTEV}*/ P[acman]-EGFP-dgo dgo*^{380}*; P[CaSpeR]-hs-TEVp/+*
(P-R) *dsh*^{V26}*/Y; P[acman]-dsh*$^{3xTEV/}$*P[CaSpeR]-hs-TEVp*
(S) *w*^{1118}*; P[acman-EGFP-dgo dgo*^{380}*/+*
dsh^{V26}*/Y; P[acman]-dsh*^{3xTEV}*/P[acman-EGFP-dgo dgo*^{380}
dsh^{V26}*/Y; P[acman]-dsh*^{3xTEV}*/P[acman]EGFP-dgo dgo*^{380}*;P[CaSpeR]-hs-TEVp/+*
(T) *w*^{1118}*; EGFP-pk/+*
dsh^{V26} *FRT19A/ubi-nls-RFP FRT19A; Ubx-FLP/P[acman]EGFP-pk*
(U) *dsh*^{V26} *FRT19A/ubi-nls-RFP FRT19A; Ubx-FLP/P[acman]EGFP-pk*
Figure S3
(A-B) *dsh*^{V26}*/Y; P[acman]-dsh-EGFP/P[CaSpeR]-hs-Rpn10-HA-vhhGFP*
(C-D) *dsh*^{1}*, shi*^{ts1}*, hs-FLP/Y; P[acman]-dsh-EGFP/P[CaSpeR]-hs-Tom70-HA-vhhGFP*
(E-F) *dsh*^{V26}*/Y; P[acman]-dsh*^{3xTEV}*/P[CaSpeR]-hs-TEVp*
(G) *w*^{1118}
(H) *w*^{1118}*; P[CaSpeR]-hs-TEVp /P[CaSpeR]-hs-TEVp*
(I) *w*^{1118}*; P[CaSpeR]-hs-Tom70-HA-vhhGFP /P[CaSpeR]-hs-Tom70-HA-vhhGFP*
(J) *w*^{1118}*; P[CaSpeR]-hs-Rpn10-HA-vhhGFP /P[CaSpeR]-hs-Rpn10-HA-vhhGFP*
(K-O) *shi*^{ts1}*, hs-FLP/Y*
Figure S4
(A) *dsh*^{V26}*/w*^{1118}*; P[acman]-dsh-EGFP/P[CaSpeR]-hs-Tom70-HA-vhhGFP*
dsh^{V26}*/w*^{1118}*; P[acman]-dsh-EGFP/P[CaSpeR]-hs-Rpn10-HA-vhhGFP*
(B) *UbxFLP, dsh*^{V26}*/w*^{1118}*;P[acman]-dsh-EGFPFRT40/FRT40;P[CaSpeR]-hs-Tom70-HA-vhhGFP/+*
(C-F, I) *dsh*^{V26} *FRT19A/ubi-mRFP-nls hs-FLP FRT19A; FRT42 EGFP-Pk/FRT42 pk-sple*^{13}
(J) *dsh*^{V26} *FRT19A/ubi-mRFP-nls FRT19A; Ubx-FLP/+*
(K-M) *UbxFLP, dsh*^{V26}*/w*^{1118}*;P[acman]-dsh-EGFPFRT40/FRT40;P[CaSpeR]-hs-Tom70-HA-vhhGFP/+*
(N) *w*^{1118}*; Act-GAL4, tub-GAL80*^{ts}*/+; UAS-KLP-10A, UAS-Katanin-60/+*
(O) *w*^{1118}
(P-S) *w*^{1118}*; EGFP-pk*

Temperature regimes for protein activity disruption

Pupae were aged for 28 hr at 25°C unless otherwise stated, except for genotypes with *hs-TEVp* on the third chromosome in which pupae were aged for twice as long at 18°C, as the transgene is leaky at 25°C. Heat-shocks were performed by placing pupae in plastic vials in a water bath at 38°C for up to 2 hr. Longer heat-shock regimes were not possible due to pupal lethality. Afterward, pupae were either immediately dissected or left to recover at 18°C for 1, 2 or 3 hr prior to use. From the same population control pupae were set aside, and aged to 28 hr APF without heat shock.

Immunostaining and antibodies

Dissection and staining procedures were performed as previously reported (Strutt et al., 2016). Briefly, pupae were fixed in 4% paraformaldehyde in PBS and wings removed. Dissected wings were fixed for 30-45 min at room temperature, depending on antibody combinations. Wings were blocked for 1 hr in PBS containing 0.2% Triton X-100 (PTX) and 10% normal goat serum prior to antibody incubation. Primary antibodies were incubated overnight at 4°C, and secondary antibodies either for 4 hr at room temperature or overnight at 4°C, in PTX with 10% normal goat serum, all washes were in PTX. After immunolabelling, wings were post-fixed in 2% paraformaldehyde in PTX for 15 min and mounted in 10% glycerol, 1xPBS, containing 2.5% DABCO (pH7.5). For super-resolution imaging, wings were mounted in Vectorshield. Primary antibodies for immunolabelling were affinity purified rabbit anti-GFP (ab6556, Abcam, UK), rat monoclonal anti-HA 3F10 (Sigma-Aldrich), affinity purified rat anti-Pk (Strutt et al., 2013b), rat anti-Dsh (Strutt et al., 2006), rabbit anti-Dsh (Strutt et al., 2013a), rat anti-Stbm (Strutt and Strutt, 2008), rabbit anti-Stbm (Warrington et al., 2013), mouse monoclonal anti-Fmi (Flamingo #74, DSHB (Usui et al., 1999)), affinity-purified rabbit anti-Fz (Bastock and Strutt, 2007), mouse anti-β-tubulin E7 (DSHB) and rat monoclonal anti-DE-cad (DSHB) (Oda et al., 1994). Actin was visualized using Alexa-568-conjugated phalloidin (Molecular Probes).

Western blotting

Pupal wings were processed for western blotting as previously described (Strutt et al., 2016). Briefly, pupal wings at 28 hr APF were dissected in 1x PBS, placed directly in 2x sample buffer on ice at a concentration of 1 wing per 10 μL and vortexed for 5 s and boiled at 95°C for 5 min to solubilise proteins. Afterward samples were vortexed for 10 s and stored at −20°C for use within a week. Before use, lysates were thawed, vortexed and 2 pupal wing equivalents were run on Tris-Bis precast gels (Invitrogen) and transferred using a wet apparatus onto a Hybond ECL nitrocellulose membrane (GE Healthcare). Membranes were blocked for 1 hr at room temperature in 5% skimmed milk in PBS containing 0.1% Tween-20. Primary antibodies were incubated overnight at 4°C and secondary antibodies at room temperature for 3 hr, in PBS containing 0.1% Tween-20 and 5% skimmed milk. Proteins were detected using affinity purified

rabbit anti-Dsh antibody (Strutt et al., 2006), mouse monoclonal anti-Fmi #74 (DHSB, (Usui et al., 1999), affinity purified rabbit anti-Fz (Bastock and Strutt, 2007), rabbit anti-Stbm (Rawls and Wolff, 2003), affinity purified rat anti-Pk (Strutt et al., 2013a) or rat anti-β-actin AC-40 (Sigma-Aldrich), and HRP-conjugated secondary antibodies (DAKO). Detection was performed using Supersignal West Dura (Pierce) and a Bio-Rad ChemiDoc XRS+ was used for imaging. Band intensities from three separate experiments were quantified using ImageJ. Values were normalized to actin loading controls and then non-heat-shocked controls. Statistical analysis was carried out using unpaired t tests or one way-ANOVA.

Cytoskeleton disruption assays

Wings from *EGFP-pk* 6 hr APF prepupae were dissected in 1% PBS. To disrupt the actin cytoskeleton, a final concentration of 2μM Latrunculin A was added to the prepupal wings for 15 min. Wings were washed twice in PBS and mounted in methyl cellulose in Schneider's medium, for live imaging within 5 min or stained for F-actin with Phalloidin-Alexa568. 6 hr APF prepupae were used as at this stage the developing wings are not enveloped by an impermeable cuticle. Severing of microtubules was performed by co-expression of *UAS-KLP10A* and *UAS-Katanin-60* (Widmann and Dahmann, 2009) at 25°C using *Act-Gal4, tub-Gal80^{ts}* and pupal wings dissected at 28 hr APF.

Heat-shock induction of tools to disrupt protein activity

All pupae were aged for 28 hr at 25°C unless otherwise stated. Heat-shocks were carried out in a water-bath at 38°C for up to 2 hr. Note that longer heat-shock regimes cause lethality. After heat-shock, pupae were either dissected, live-imaged, or left to recover at 18°C for 1, 2 or 3 hr. For experiments to block dynamin-dependent endocytosis, pupae hemizygous for *shi^{ts1}* were aged at 18°C, then heat-shocked for 2 hr.

Imaging

All confocal micrographs of fixed pupal wings were captured posterior to the L4 vein region of the pupal wing. Fixed pupal wings were imaged on a Nikon A1R GaAsP confocal microscope using a 60x NA1.4 apochromatic lens, with a pixel size of 70 nm, and a pinhole of 1.2 AU. 9 Z-slices separated by 150 nm were imaged, and then the 3 brightest slices around junctions were selected and averaged for each channel in ImageJ. Super-resolution imaging was carried out using a Zeiss LSM 710 AiryScan with a 63x lens.

For live imaging, white prepupae were collected and aged for 28 hr at 25°C (or the equivalent time at different temperatures (Warrington et al., 2017)). Briefly, a small piece of cuticle was removed from above the pupal wing, and the exposed wing was mounted in a drop of Halocarbon 700 oil in a glass-bottomed dish. For FRAP analysis, images were 256 × 256 pixels, with a pixel size of 100 nm, 60x 1.2 NA oil objective and a pinhole of 1.2 AU. For FRAP, regions of interest (ROIs) of ~3 μm^2 were selected as ellipses that covered 2/3 of proximal-distal membranes, where planar polarity proteins localize, with the exception of the experiment in Fig.S4I where any junctions on the boundary were bleached. Three prebleach images were taken at 2 frames/sec, and ROIs were then bleached using a 488 nm Argon laser at 85% with eight laser passes (one second total time), which resulted in 55%–70% bleaching. Immediately following bleaching, five images were taken at 5 s intervals, followed by 10 images at 10 s intervals, 10 images at 15 s intervals and 8 images at 30 s intervals. Laser power was adjusted to maintain constant power at the lens between different imaging sessions. When only EGFP was being imaged, a 488 nm laser and a long pass GFP filter were used. For samples expressing both EGFP and mRFP, a 488 nm laser and a 525-550 band pass filter were used to detect EGFP. After imaging, ROIs were manually reselected in ImageJ and quantitated, in addition to four unbleached regions to control for acquisition bleaching. Stable amounts were compared using ANOVA.

QUANTIFICATION AND STATISTICAL ANALYSIS

FRAP processing

Data analysis was conducted as previously described (Strutt et al., 2011). Briefly, ImageJ was used to manually reselect up to 8 bleached regions in each image for each time point. The laser-off background was subtracted, and the values were then corrected for acquisition bleaching and normalized against the average of the prebleach values. Data were then plotted on an xy graph using Prism (v7 Graphpad), bleached regions within the same wing were averaged and a one-phase exponential curve was fitted for each wing. Multiple wings were then combined and a one phase exponential association curve was fitted. To determine the stable amount, the mean intensity of the ROIs from the three prebleach images was measured in ImageJ, and averaged per wing. The intensity was then corrected for distance from the coverslip as previously described (Strutt et al., 2016), and this value multiplied by the stable fraction (1-y[max]) for each wing. The stable amounts were then averaged across wings, and results were plotted on a scatter graph along with the mean and standard deviation. Statistical tests were performed using one-way ANOVA, with Dunnett's multiple comparison test to compare a control to the rest of the genotypes in the experiment, Tukey-Kramer's multiple comparison test to compare all genotypes within an experiment and the Holm-Šídák multiple comparison test for paired comparisons within an experiment. p values calculated in Prism 7 are reported on the graph as asterisks (* = $p \leq 0.5$, ** = $p \leq 0.01$, *** = $p \leq 0.001$). See also Table S2 and S3 for the raw data and results of the statistical tests.

Based on the mean intensity and standard deviation of a control set of wings, we aimed for a sample size of at least 6 wings per genotype. This would allow detection of differences of 20% in the means, in a pairwise comparison (calculated using G Power).

Each experiment was performed on multiple wings from different pupae, which represent biological replicates (n, number of wings). For each wing, 8 ROIs were selected for FRAP analysis, and these were treated as technical replicates and were averaged per wing to produce a y[max] and a stable amount per wing. Data were excluded if the ROI recovery curve failed the 'replicates test for lack of fit' in Prism, or if the wing moved out of focus during the course of imaging. In total 22 wings were discarded across all the genotypes.

Statistics

The overall intensities and stable and unstable amounts for multiple genotypes were compared using one-way ANOVA, to take into account the sample variation across the genotypes analyzed and to avoid multiple t test analysis. Post hoc tests were used to compare individual samples: Dunnett's multiple comparison test was used to compare the control to the rest of the genotypes in the experiment; Tukey-Kramer's multiple comparison test to compare all genotypes within an experiment; and Holm-Šídák's multiple comparison test was sometimes used to compare genotypes pairwise. Where a post hoc test was used this is described in the Figure legends, and multiplicity adjusted p values calculated in Prism are reported on the graph as asterisks (* = $p \leq 0.5$, ** = $p \leq 0.01$, *** = $p \leq 0.001$).

Polarity measurement

A MATLAB script was used to determine the angle of maximum asymmetry for each cell (see (Strutt et al., 2016) for MATLAB scripts). The mean vector polarity was then averaged for all cells in the image to give a mean vector polarity per wing (asymmetry ratio on plots). Results were averaged across wings and compared using ANOVA with Tukey's multiple comparison test.

Quantification of Pk propagation

Pk cytoplasmic levels were selected in immunolabelled confocal images inside and outside acute knockdown clone tissue using ImageJ. A circular shape was drawn inside each cell (without touching the membrane) and the same shape used throughout all measurements. The mean intensity was determined for each cell, and averaged to give a mean intensity per wing. Results were averaged across wings and ANOVA with Dunnett's multiple comparison test was used to compare cells away from the clone boundary to cells just inside the clone.

Quantification of Pk membrane levels

Pk membrane levels were selected in immunolabelled confocal images inside and outside acute knockdown tissue using ImageJ. A line was drawn on each vertical cell junction and the same shape used throughout all measurements. A minimum of 5 cell membrane measurements were taken per wing and averaged to give a mean intensity per wing. Results were averaged across wings and compared using a paired parametric t test or a paired one-way ANOVA.

Cell Reports, Volume *25*

Supplemental Information

Rapid Disruption of Dishevelled Activity Uncovers an Intercellular Role in Maintenance of Prickle in Core Planar Polarity Protein Complexes

Margarida Ressurreição, Samantha Warrington, and David Strutt

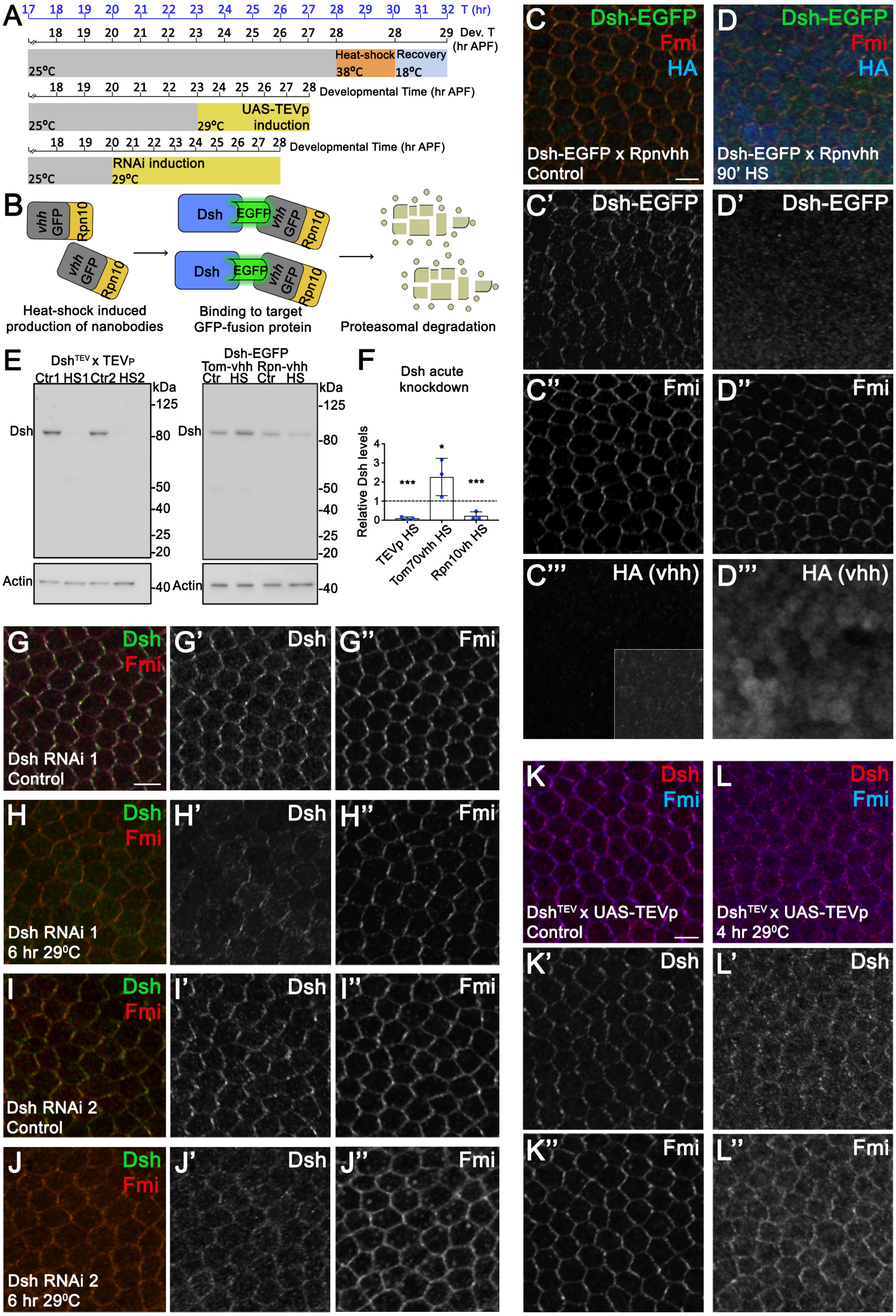

A
T (hr)
Dev. T (hr APF)
Heat-shock 38°C
Recovery 18°C
25°C
Developmental Time (hr APF)
UAS-TEVp induction
29°C
RNAi induction
B
Heat-shock induced production of nanobodies
Binding to target GFP-fusion protein
Proteasomal degradation
Dsh
EGFP
vhh GFP
Rpn10
E
Dsh^TEV x TEVp
Ctr1 HS1 Ctr2 HS2
Dsh-EGFP
Tom-vhh Rpn-vhh
Ctr HS Ctr HS
kDa
Dsh
Actin
F
Dsh acute knockdown
Relative Dsh levels
TEVp HS
Tom70vhh HS
Rpn10vh HS
C Dsh-EGFP Fmi HA
Dsh-EGFP x Rpnvhh Control
D Dsh-EGFP Fmi HA
Dsh-EGFP x Rpnvhh 90' HS
C' Dsh-EGFP
D' Dsh-EGFP
C'' Fmi
D'' Fmi
C''' HA (vhh)
D''' HA (vhh)
G Dsh Fmi
Dsh RNAi 1 Control
G' Dsh
G'' Fmi
H Dsh Fmi
Dsh RNAi 1 6 hr 29°C
H' Dsh
H'' Fmi
I Dsh Fmi
Dsh RNAi 2 Control
I' Dsh
I'' Fmi
J Dsh Fmi
Dsh RNAi 2 6 hr 29°C
J' Dsh
J'' Fmi
K Dsh Fmi
Dsh^TEV x UAS-TEVp Control
L Dsh Fmi
Dsh^TEV x UAS-TEVp 4 hr 29°C
K' Dsh
L' Dsh
K'' Fmi
L'' Fmi

Figure S1. Disruption of Dishevelled activity in the *Drosophila* pupal wing. Related to Figure 1.

(A) Diagram representing the temperature regimes employed to knockdown Dishevelled acutely, via RNAi or UAS-TEVp in the 28 hr APF wing epithelium. 'Dev. T' is the developmental time equivalent at 25°C.

(B) Dsh-EGFP disruption based on targeting with anti-GFP nanobodies fused to the proteosomal subunit Rpn10. Upon Rpn10-HA-vhhGFP production via heat-shock, Dsh-EGFP is targeted for degradation.

(C,D) 28 hr APF wing epithelium heterozygous for *dsh-EGFP* and *hs-Rpn10-HA-vhhGFP* in a dsh^{V26} null background. (C) No heat-shock, showing native GFP (green, C'), endogenous Fmi expression (red, C'') and HA labelling (blue, C'''). Inset is a region in C''' where intensity was digitally increased to show there is negligible HA expression. (D) Disruption of Dsh-EGFP by Rpn10-HA-vhhGFP expression after induction by a 90 min heat-shock at 38°C. (D') is GFP, (D'') endogenous Fmi expression and (D''') HA labelling showing strong cytoplasmic signal corresponding to expression of Rpn10-HA-vhhGFP. Scale bars = 5µm and are the same hereafter.

(E) Western blots showing levels of either Dsh^{TEV} or Dsh-GFP proteins before and after acute knockdown via heat-shock (38°C) when in the presence of *hs-TEVp, hs-Tom70-HA-vhhGFP* or *hs-Rpn10-HA-vhhGFP,* in a dsh^{V26} background. The left blot shows two independent experiments before and after Dsh^{TEV} cleavage by TEVp. Lanes labelled Ctr are Dsh^{TEV} in the presence of *hs-TEVp* without heat-shock induction, showing only one band at the expected molecular weight for Dsh. Lanes labelled HS are of Dsh^{TEV} in the presence of *hs-TEVp* after a 2 hr heat-shock and 1 hr recovery at 18°C, showing negligible detection of Dsh. The blot on the right shows Dsh-GFP before and after induction of Tom70-HA-vhhGFP or Rpn10-HA-vhhGFP. Lanes labelled Ctr are of Dsh-EGFP in the presence of the two differently anchored anti-GFP nanobodies, without heat-shock induction. Lanes labelled HS are of Dsh-EGFP in the presence of Tom70-HA-vhhGFP or Rpn10-HA-vhhGFP after a 2 hr or a 90 min heat-shock at 38°C, respectively. Note that Dsh-EGFP is still present after the production of Tom70-HA-vhhGFP as this presumably relocates Dsh-EGFP from the membrane to the mitochondrial network. Meanwhile Dsh-EGFP is visibly reduced after the production of Rpn10-HA-vhhGFP as this should relocate Dsh-EGFP to proteasomes.

(F) Quantification of the relative change in either Dsh^{TEV} or Dsh-EGFP protein levels when disrupted using TEVp, Tom70-HA-vhhGFP or Rpn10-HA-vhhGFP. Each data point is an average of the replicates of three independent experiments, normalised to actin and then to the control. Error bars are standard deviation. Unpaired t-tests were used to compare experimental and control samples, ***$p \leq 0.001$.

(G-J) 28 hr APF wing epithelia from male flies carrying two different RNAi lines against *dsh* transcripts and *Act-GAL4, tub-GAL80^ts^*. (G,I) When maintained at 25°C for 28 hr, wing tissue shows normal asymmetric planar polarised localisation of endogenous Dsh (G', I') and Fmi (G'', I''). (H) Upon a temperature shift regime to 29°C for 6 hr to induce the production of $dsh^{NIG18361R\text{-}2}$ RNAi, Dsh labelling is still evident, albeit patchy (H') and Fmi labelling is still present and asymmetric (H''). (J) Upon a temperature shift to 29°C for 6 hr to induce dsh^{WIZ} RNAi expression, Dsh (J') and Fmi (J'') labelling is still detectable at the cell membrane.

(K-L) 28 hr APF wing epithelia heterozygous for Dsh^{TEV}, *UAS-TEVp* and *Act-GAL4, tub-GAL80*^{ts}*,* in a dsh^{V26} null background. (K) Pupae maintained at 25°C for 28 hr show a normal asymmetric localisation of Dsh^{TEV} (K') and Fmi (K'') suggesting that TEVp production is negligible under these conditions. (L-L'') Upon a temperature shift to 29°C for 4 hr, Dsh (L') and Fmi (L'') labelling is still asymmetrically present at the cell membrane, however localisation is weaker and more diffuse.

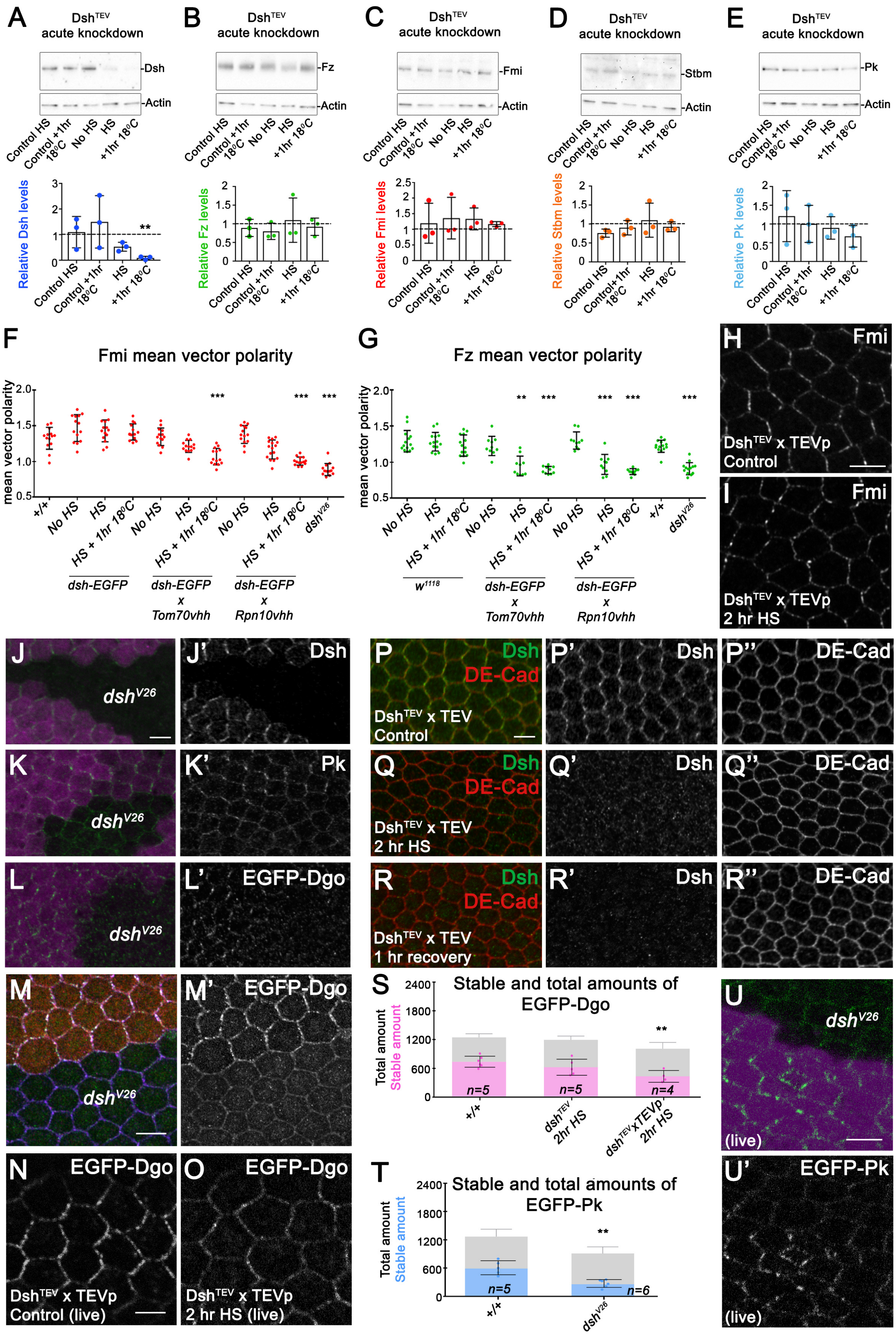

A
DshTEV acute knockdown
Dsh
Actin
Control HS
Control +1hr 18°C
No HS
HS
+1hr 18°C
Relative Dsh levels
**
B
DshTEV acute knockdown
Fz
Actin
Relative Fz levels
C
DshTEV acute knockdown
Fmi
Actin
Relative Fmi levels
D
DshTEV acute knockdown
Stbm
Actin
Relative Stbm levels
E
DshTEV acute knockdown
Pk
Actin
Relative Pk levels
F
Fmi mean vector polarity
mean vector polarity
+/+
No HS
HS
HS + 1hr 18°C
dsh-EGFP
dsh-EGFP x Tom70vhh
dsh-EGFP x Rpn10vhh
dshV26
G
Fz mean vector polarity
w1118
H
Fmi
DshTEV x TEVp
Control
I
Fmi
DshTEV x TEVp
2 hr HS
J
dshV26
J'
Dsh
K
dshV26
K'
Pk
L
dshV26
L'
EGFP-Dgo
M
dshV26
M'
EGFP-Dgo
N
EGFP-Dgo
DshTEV x TEVp
Control (live)
O
EGFP-Dgo
DshTEV x TEVp
2 hr HS (live)
P
Dsh
DE-Cad
DshTEV x TEV
Control
P'
Dsh
P''
DE-Cad
Q
Dsh
DE-Cad
DshTEV x TEV
2 hr HS
Q'
Dsh
Q''
DE-Cad
R
Dsh
DE-Cad
DshTEV x TEV
1 hr recovery
R'
Dsh
R''
DE-Cad
S
Stable and total amounts of EGFP-Dgo
Total amount
Stable amount
n=5
n=5
n=4
+/+
dshTEV 2hr HS
dshTEVxTEVp 2hr HS
T
Stable and total amounts of EGFP-Pk
Total amount
Stable amount
n=5
n=6
+/+
dshV26
U
dshV26
(live)
U'
EGFP-Pk
(live)

Figure S2. Dishevelled maintains stability of core planar polarity proteins. Related to Figure 2.

(A-E) Western blots from 28 hr APF pupal wing extracts, showing protein levels of either Dsh^{TEV} (A), Fz (B), Fmi (C), Stbm (D) or Pk (E) before and after Dsh^{TEV} acute knockdown. Lanes labelled Control HS and Control + 1hr 18°C are immediately after, and one hour after heat-shock induction of *hs-TEVp* with wild-type Dsh as a control for the temperature regimes. Lanes labelled No HS, HS and + 1hr 18°C, are from flies expressing Dsh^{TEV} in a *dsh*^{V26} and *hs-TEVp* background before, after a 2 hr heat-shock, and 1 hr at 18°C after a 2 hr heat-shock regime. Below each blot is the quantification for each detected protein from three independent experiments normalised to actin and then to the No HS condition. Error bars are standard deviation. ANOVA with Dunnett's multiple comparisons test were used to compare experimental to no heat-shock control samples, $**p \leq 0.01$.

(F,G) Fmi (F) and Fz (G) mean vector polarity values after disruption of Dsh-EGFP using vhhGFP. Error bars are standard deviation. ANOVA with Tukey-Kramer's multiple comparison test was used to compare all samples. Shown are comparisons of each genotype to no heat-shock conditions and +/+ to *dsh*^{V26} clone tissue. $***p \leq 0.001$, $**p \leq 0.01$.

(H,I) AiryScan super-resolution microscopy of 28 hr APF wings, immunolabelled for Fmi, before (H) and after (I) Dsh^{TEV} cleavage.

(J-L) Immunolabelling of 28 hr APF wings containing clones of *dsh*^{V26} null mutant tissue (loss of RFP, magenta), labelled for Dsh (green, J), Pk (green, K) and EGFP-Dgo (green, L). Scale bars = 5µm and are the same hereafter.

(M) AiryScan super-resolution image of EGFP-Dgo in wild-type and *dsh*^{V26} null mutant tissue. While EGFP-Dgo is hard to visualise at cell membranes of *dsh*^{V26} mutant tissue by confocal microscopy (L'), an EGFP-Dgo signal at cell junctions is weakly detected using AiryScan microscopy (M').

(N,O) Live imaging of 28 hr APF wings expressing EGFP-Dgo before (N) and after (O) cleavage of Dsh^{TEV}.

(P-R) Dsh^{TEV} (green) and E-cadherin (red) immunolabelling of 28 hr APF wings before cleavage of Dsh^{TEV} (P), immediately after (Q) and 1 hr after (R), showing the sustained absence of Dsh labelling after heat-shock induction.

(S) FRAP analysis of 28 hr APF pupal wings expressing EGFP-Dgo, showing stable and total amounts of EGFP-Dgo before and after Dsh^{TEV} cleavage. Error bars are standard deviation, n = number of wings. ANOVA with Tukey's multiple comparison test was used to compare all genotypes, p=0.0088 comparing +/+ to Dsh^{TEV}xTEVp. Also see Table S2 and Table S3.

(T) FRAP analysis of 28 hr APF pupal wings expressing EGFP-Pk in wild-type Dsh and *dsh* null tissue. Error bars are standard deviation, n = number of wings. Genotypes were compared using an unpaired t-test, p=0.0011. Also see Table S2 and Table S3.

(U) Live imaging of 28 hr APF wings containing clones of *dsh*^{V26} null mutant tissue (loss of RFP, magenta), expressing EGFP-Pk (green, U').

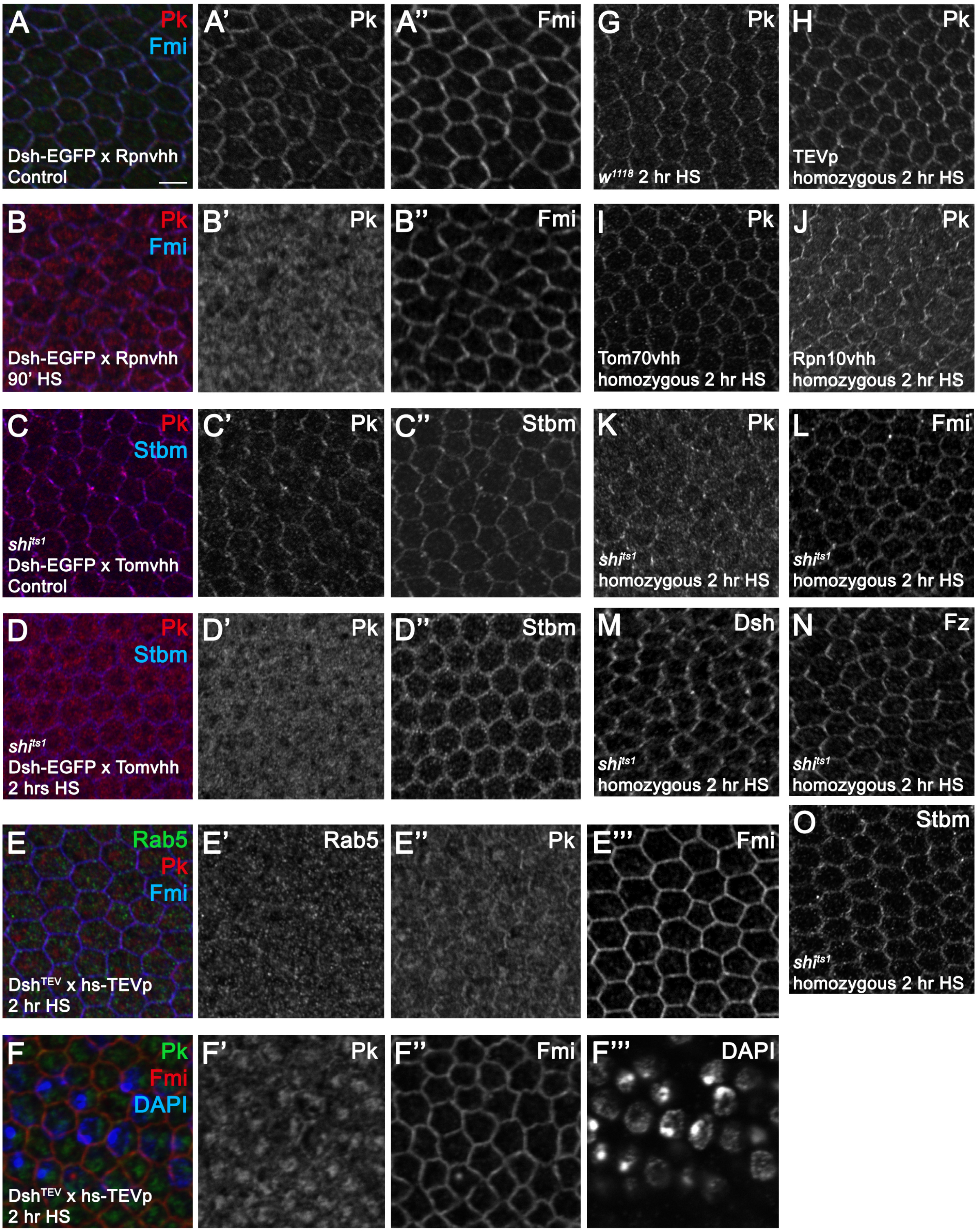
A
Pk
Fmi
Dsh-EGFP x Rpnvhh
Control
A'
Pk
A''
Fmi
G
Pk
w1118 2 hr HS
H
Pk
TEVp
homozygous 2 hr HS
B
Pk
Fmi
Dsh-EGFP x Rpnvhh
90' HS
B'
Pk
B''
Fmi
I
Pk
Tom70vhh
homozygous 2 hr HS
J
Pk
Rpn10vhh
homozygous 2 hr HS
C
Pk
Stbm
shits1
Dsh-EGFP x Tomvhh
Control
C'
Pk
C''
Stbm
K
Pk
shits1
homozygous 2 hr HS
L
Fmi
shits1
homozygous 2 hr HS
D
Pk
Stbm
shits1
Dsh-EGFP x Tomvhh
2 hrs HS
D'
Pk
D''
Stbm
M
Dsh
shits1
homozygous 2 hr HS
N
Fz
shits1
homozygous 2 hr HS
E
Rab5
Pk
Fmi
DshTEV x hs-TEVp
2 hr HS
E'
Rab5
E''
Pk
E'''
Fmi
O
Stbm
shits1
homozygous 2 hr HS
F
Pk
Fmi
DAPI
DshTEV x hs-TEVp
2 hr HS
F'
Pk
F''
Fmi
F'''
DAPI

Figure S3. Removal of Dsh from core planar polarity protein complexes results in the translocation of Pk from the membrane to the cytoplasm. Related to Figure 3.

(A,B) 28 hr APF pupal wings before and after sequestration of Dsh-EGFP by Rpn10-HA-vhhGFP, showing Pk (A' and B') and Fmi (A'' and B''). Scale bar = 5µm.

(C,D) Immunolabelling of 28 hr APF wings hemizygous for shi^{ts1} before (C) and after (D) sequestration of Dsh-EGFP by Tom70-HA-vhhGFP. (C) Pk (C') and Stbm (C'') localise asymmetrically. (D) Pk (D') is cytoplasmic and Stbm (D'') is junctional.

(E) 28 hr APF pupal wings after cleavage of Dsh^{TEV}, showing negligible colocalisation between Rab5 (E') and cytoplasmic Pk (E''), Fmi staining defines the cell junctions (E''').

(F) 28 hr APF pupal wings after cleavage of Dsh^{TEV}, showing DAPI (F''') and cytoplasmic Pk (F'), Fmi staining defines the cell junctions (F'').

(G-J) Immunolabelling of 28 hr APF wings from wild-type (G), *hs-TEVp* (H), *hs-Tom70-HA-vhhGFP* (I) or *hs-Rpn10-HA-vhhGFP* (J) to assess non-specific effects on Pk localisation after a 2 hr heat-shock.

(K-O) Immunolabelling of 28 hr APF wings from shi^{ts1} flies, to assess non-specific effects on Pk (K), Fmi (L), Dsh (M), Fz (N) and Stbm (O) localisation after a 2 hr heat-shock.

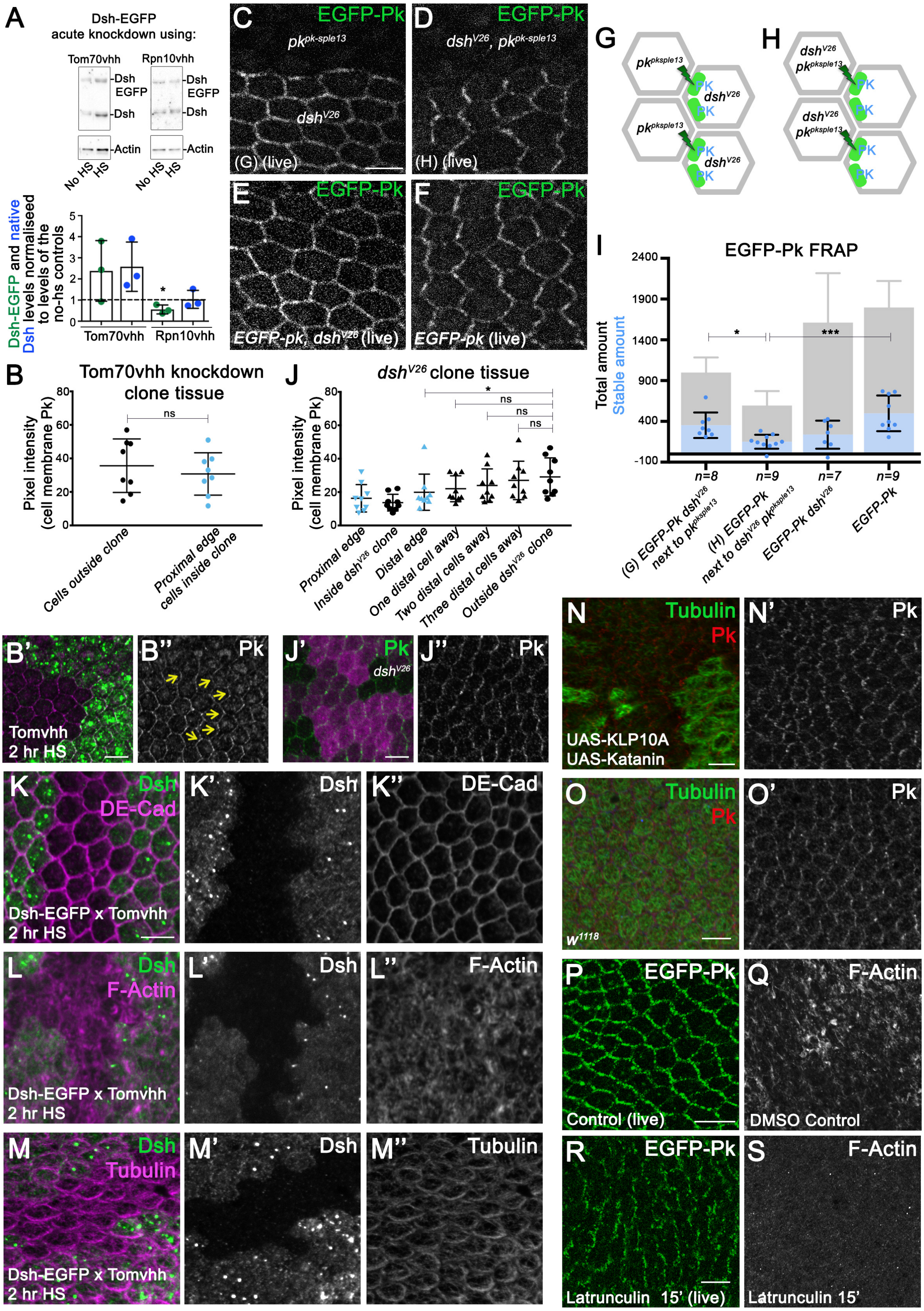

A
Dsh-EGFP acute knockdown using:
Tom70vhh
Rpn10vhh
-Dsh EGFP
-Dsh
-Actin
No HS
HS
Dsh-EGFP and native Dsh levels normalised to levels of the no-hs controls
Tom70vhh
Rpn10vhh
B
Tom70vhh knockdown clone tissue
Pixel intensity (cell membrane Pk)
ns
Cells outside clone
Proximal edge cells inside clone
C
EGFP-Pk
$pk^{pk-sple13}$
dsh^{V26}
(G) (live)
D
EGFP-Pk
dsh^{V26}, $pk^{pk-sple13}$
(H) (live)
E
EGFP-Pk
EGFP-pk, dsh^{V26} (live)
F
EGFP-Pk
EGFP-pk (live)
G
H
I
EGFP-Pk FRAP
Total amount
Stable amount
n=8
n=9
n=7
n=9
(G) EGFP-Pk dsh^{V26} next to $pk^{pksple13}$
(H) EGFP-Pk next to dsh^{V26} $pk^{pksple13}$
EGFP-Pk dsh^{V26}
EGFP-Pk
J
dsh^{V26} clone tissue
Pixel intensity (cell membrane Pk)
Proximal edge
Inside dsh^{V26} clone
Distal edge
One distal cell away
Two distal cells away
Three distal cells away
Outside dsh^{V26} clone
B'
Tomvhh 2 hr HS
B''
Pk
J'
Pk
dsh^{V26}
J''
Pk
K
Dsh
DE-Cad
Dsh-EGFP x Tomvhh 2 hr HS
K'
Dsh
K''
DE-Cad
L
Dsh
F-Actin
Dsh-EGFP x Tomvhh 2 hr HS
L'
Dsh
L''
F-Actin
M
Dsh
Tubulin
Dsh-EGFP x Tomvhh 2 hr HS
M'
Dsh
M''
Tubulin
N
Tubulin
Pk
UAS-KLP10A UAS-Katanin
N'
Pk
O
Tubulin
Pk
w^{1118}
O'
Pk
P
EGFP-Pk
Control (live)
Q
F-Actin
DMSO Control
R
EGFP-Pk
Latrunculin 15' (live)
S
F-Actin
Latrunculin 15'

Figure S4. Dsh acts on Pk cell non-autonomously and independently of the cytoskeleton. Related to Figure 4.

(A) Western blots from 28 hr APF pupal wing extracts, showing levels of Dsh-GFP and native Dsh proteins before and after acute knockdown using *hs-Tom70-HA-vhhGFP* or *hs-Rpn10-HA-vhhGFP,* in a heterozygous dsh^{V26} background. Lanes labelled No HS show Dsh-EGFP and native Dsh in a background containing the anti-GFP nanobody transgenes, without heat-shock induction. Lanes labelled HS show Dsh-EGFP and native Dsh in the presence of Tom70-HA-vhhGFP or Rpn10-HA-vhhGFP after a 2 hr or a 90 min heat-shock at 38°C, respectively. Quantitation of Dsh-EGFP and Dsh levels, from three independent experiments is shown below, normalised to actin and then to the levels in No HS. Note that Dsh-EGFP is still present (and indeed shows increased levels) after the production of Tom70-HA-vhhGFP; while it is significantly reduced in the presence of Rpn10-HA-vhhGFP. Meanwhile native Dsh levels also increase after production of Tom70-HA-vhhGFP (presumably due to co-sequestration to mitochondria by Dsh-EGFP) while they do not decrease after the production of Rpn10-HA-vhhGFP. *p=0.05.

(B) Quantification of proximodistal membrane localised Pk in cells outside and at the proximal edge of cells at the proximal edge of Dsh-EGFP clones in Tom70-vhh knockdown tissue, and representative images (B',B'', yellow arrows indicate Pk at proximal edge of cells at proximal edge of clones). Error bars are standard deviation, each data point is one wing. A paired t-test was used to compare measurements between the outside and the proximal edge of the clone.

(C-I) FRAP analysis of EGFP-Pk on boundaries of dsh^{V26} null clones that border cells expressing wild-type levels of Dsh. (C-H) Representative images and diagrams, showing EGFP-Pk in a wild-type (F) or dsh^{V26} (E) background, or clones where EGFP-Pk is expressed in dsh^{V26} cells next to $pk^{pk\text{-}sple13}$ mutant cells (C,G) or in wild-type cells next to double-mutant dsh^{V26} and $pk^{pk\text{-}sple13}$ cells (D,H). Images show EGFP-Pk in white. Cells lacking EGFP-Pk were made mutant for $pk^{pk\text{-}sple13}$, to avoid possible confounding effects of Dsh on these unlabelled populations of Pk. (I) FRAP of EGFP-Pk (left to right): First column – boundaries between dsh^{V26} *EGFP-Pk* cells and $pk^{pk\text{-}sple13}$ cells; second column – boundaries between *EGFP-Pk* cells and dsh^{V26} $pk^{pk\text{-}sple13}$ cells; third and fourth columns – EGFP-Pk on vertical orientated junctions in a dsh^{V26} background or a wild-type background. The stable amount of EGFP-Pk in wild-type tissue (blue bar in fourth column) is significantly higher compared to that of EGFP-Pk in wild-type cells neighbouring cells that lack Dsh (blue bar in second column) ***$p \leq 0.001$. While loss of Dsh in the same cell as EGFP-Pk (blue bar in first column) reduces overall EGFP-Pk levels (reduced height of grey bar in first column), the stable amount of EGFP-Pk is not significantly different to that of EGFP-Pk in wild-type cells (fourth column) $p=0.2094$. In addition, the stable amount of EGFP-Pk in a wild-type cell next to dsh^{V26} minus cells (second column) is decreased compared to EGFP-Pk in dsh^{V26} cells next to wild-type cells (first column) *$p \leq 0.05$. Error bars are standard deviation, n = number of wings. ANOVA and a Holm-Šídák multiple comparison test was used to compare columns 1 and 2, 1 and 3, and 2 and 4. Also see Table S2.

(J) Quantification of membrane localised Pk at the proximal edge of cells at the proximal edge of dsh^{V26} clone tissue, inside clone tissue, at the distal cell edge at the distal edge of clone tissue, and at cell edges one to three distal cells away from, clone tissue; with a representative image (J'). Error bars are standard deviation, each data point is one wing. One-way ANOVA and a Tukey's multiple comparison test was used to compare measurements from outside dsh^{V26} tissue with all other measurements, *$p \leq 0.05$.

(K-M) Images of *Drosophila* wing epithelia at 28 hr APF. Clone tissue is two copies of Dsh-EGFP or no Dsh-EGFP and heterozygous tissue is one copy of Dsh-EGFP, all in a background heterozygous for *hs-Tom70-HA-vhhGFP* and dsh^{V26}. After a 2 hr heat-shock, Dsh-EGFP is sequestered away from cell junctions (K', L' and M'). E-cad (K''), F-actin (L'') and β-Tubulin (M'') show no change in distribution between knockdown and non-targeted tissue.

(N) β-Tubulin and Pk localisation in tissue heterozygous for *Act-GAL4, tub-GAL80*^{ts}, and *UAS-KLP10A*, *UAS-Katanin-60*, maintained at 25°C until 28 hr APF. Under these conditions there is leaky expression of the transgenes, resulting in patchy disruption of microtubules in a stochastic manner. Pk remains at the membrane even in the absence of a visible microtubule network.

(O) β-Tubulin (green) and Pk (red) localization in w^{1118} wing epithelial cells at 25°C (28 hr APF), showing normal distributions.

(P,Q) 6 hr APF prepupal wings incubated for 15 min in 0.1% DMSO. (P) Live imaging of EGFP-Pk shows a normal distribution for that developmental stage. (Q) F-actin distribution detected by Phalloidin-Alexa568 in fixed wings.

(R,S) 6 hr APF prepupal wings incubated for 15 min in 2µM of Latrunculin A diluted in DMSO. (R) Live imaging of EGFP-Pk shows a membranous distribution. (S) F-actin detected by Phalloidin-Alexa568 in fixed wings. Note substantial loss of F-actin network.

Genotype	Treatment	n = number of wings analysed	Mean Vector Polarity	s.d.	*p* values Comparing to No HS condition	*p* values Comparing to w^{1118}
**Fmi immunolabelling**						
w^{1118}	25ºC (No HS)	14	1.363	0.137	n/a	n/a
w^{1118}	2 hr (38ºC)	14	1.321	0.099	0.951	n/a
w^{1118}	2 hr (38ºC) + 1 hr (18ºC)	14	1.375	0.224	0.892	n/a
$dsh^{V26}/Y; dsh^{TEV}$	25ºC (No HS)	9	1.400	0.137	n/a	0.875
$dsh^{V26}/Y; dsh^{TEV}$	2 hr (38ºC)	10	1.351	0.245	0.902	>0.999
$dsh^{V26}/Y; dsh^{TEV}$	2 hr (37ºC) + 1 hr (18ºC)	7	1.404	0.121	0.659	0.900
hs-TEVp	25ºC (No HS)	7	1.514	0.067	n/a	1.000
hs-TEVp	2 hr (38ºC)	10	1.581	0.169	0.998	0.315
hs-TEVp	2 hr (38ºC) + 1 hr (18ºC)	7	1.559	0.131	0.997	0.850
$dsh^{V26}/Y; dsh^{TEV}$/ *hs-TEVp*	25ºC (No HS)	21	1.365	0.168	n/a	>0.999
$dsh^{V26}/Y; dsh^{TEV}$/ *hs-TEVp*	2 hr (38ºC)	21	1.266	0.109	0.055	0.829
$dsh^{V26}/Y; dsh^{TEV}$/ *hs-TEVp*	2 hr (38ºC) + 1 hr (18ºC)	21	1.085	0.121	0.000	0.043
dsh^{V26}/Y; *dsh-EGFP*	25ºC (No HS)	10	1.470	0.153	n/a	0.805
dsh^{V26}/Y; *dsh-EGFP*	2 hr (38ºC)	10	1.341	0.206	>0.999	0.875
dsh^{V26}/Y; *dsh-EGFP*	2 hr (38ºC) + 1 hr (18ºC)	10	1.435	0.188	>0.999	0.875
hs-Tom70-HA-vhhGFP	25ºC (No HS)	8	1.314	0.155	n/a	>0.999
hs-Tom70-HA-vhhGFP	2 hr (38ºC)	9	1.330	0.141	>0.999	>0.999
hs-Tom70-HA-vhhGFP	2 hr (38ºC) + 1 hr (18ºC)	9	1.269	0.130	0.999	0.893
dsh^{V26}/Y; *dsh-EGFP/ hs-Tom70-HA-vhhGFP*	25ºC (No HS)	14	1.345	0.123	n/a	0.385
dsh^{V26}/Y; *dsh-EGFP/ hs-Tom70-HA-vhhGFP*	2 hr (38ºC)	14	1.211	0.084	0.163	0.132
dsh^{V26}/Y; *dsh-EGFP/ hs-Tom70-HA-vhhGFP*	2 hr (38ºC) + 1 hr (18ºC)	14	1.067	0.115	<0.0001	<0.0001
hs-Rpn10-HA-vhhGFP	25ºC (No HS)	7	1.190	0.178	n/a	0.967
hs-Rpn10-HA-vhhGFP	90 min (38ºC)	15	1.200	0.229	0.992	0.999
hs-Rpn10-HA-vhhGFP	90 min (38ºC) + 1 hr (18ºC)	8	1.107	0.132	0.929	0.967
dsh^{V26}/Y; *dsh-EGFP/ hs-Rpn10-HA-vhhGFP*	25ºC (No HS)	14	1.378	0.125	n/a	0.989
dsh^{V26}/Y; *dsh-EGFP/ hs-Rpn10-HA-vhhGFP*	2 hr (38ºC)	16	1.197	0.233	0.053	0.053
dsh^{V26}/Y; *dsh-EGFP/ hs-Rpn10-HAvhhGFP*	2 hr (38ºC) + 1 hr (18ºC)	14	1.004	0.057		<0.0001

Table S1. Related to Figure 2 and S2.

Mean vector polarity measurements of Fmi immunolabelling in 28 hr APF pupal wing tissue for all genotypes before and after heat-shock. Vector polarity was calculated for a group of cells within a field of view in an individual wing. 'n' indicates the numbers of wings examined. Results were averaged across wings and standard deviations (s.d.) calculated. ANOVA with Tukey's multiple comparison test was used to compare between genotypes. In column 6 ('No hs') are the results of comparing the heat-shocked samples to the non heat-shocked samples within a genotype. In column 7 ('w^{1118}') are the results of comparing each heat-shock regime to control w^{1118} wings treated to the same heat-shock regime.

Fz-EGFP before and after cleavage of Dsh^{TEV}

Genotype	n	Total fluorescent mean intensity	Total mean intensity confidence intervals	Stable amount	Stable amount 95% confidence intervals	y[max]	y[max] with 95% confidence intervals	t[0.5] Half-life	Half-life with 95% confidence Intervals	Figure
w^{1118}*; fz-EGFP/+*	6	2583.00	2363 to 2803	1588.00	1377 to 1799	0.36	0.335 to 0.385	48.07	38.71 to 63.39	Fig.2N
dsh^{V26}*/Y; P[acman]-dsh*^{TEV}*/+; fz-EGFP/+* (2 hr HS)	6	2539.00	2066 to 3012	1393.00	1173 to 1612	0.46	0.371 to 0.551	40.22	0.304 to 0.3404	Fig.2N
dsh^{V26}*/Y; P[acman]-dsh*^{TEV}*/+; fz-EGFP/+* (2 hr HS + 18°C)	6	2208.00	1921 to 2388	1444.00	1191 to 1697	0.36	0.294 to 0.422	60.58	0.367 to 0.420	Fig.2N
dsh^{V26}*/Y; P[acman]-dsh*^{TEV}*/P[CaSpeR]-hs-TEVp; fz-EGFP/+* (No HS)	7	2216.00	1852 to 2580	1365.00	1190 to 1541	0.40	0.300 to 0.490	66.36	55.93 to 81.56	Fig.2N
dsh^{V26}*/Y; P[acman]-dsh*^{TEV}*/P[CaSpeR]-hs-TEVp; fz-EGFP/+* (2 hr HS)	7	2472.00	1936 to 3008	1054.00	889.5 to 1218	0.58	0.545 to 0.613	66.84	55.12 to 84.92	Fig.2N
dsh^{V26}*/Y; P[acman]-dsh*^{TEV}*/P[CaSpeR]-hs-TEVp; fz-EGFP/+* (2 hr HS + 18°C)	6	2386.50	647 to 2322	845.20	671.1 to 1019	0.65	0.602 to 0.703	36.24	29.77 to 46.31	Fig.2N
dsh^{V26} *FRT19A/ ubi-mRFP-nls FRT19A; Ubx-FLP/+; fz-EGFP/+*	6	2403.60	373.6 to 2144	833.90	534.9 to 1133	0.66	0.605 to 0.713	26.94	20.75 to 38.38	Fig.2N

Fmi-EGFP before and after cleavage of Dsh^{TEV}

Genotype	n	Total fluorescent mean intensity	Total mean intensity confidence intervals	Stable amount	Stable amount 95% confidence intervals	y[max]	y[max] with 95% confidence intervals	t[0.5] Half-life	Half-life with 95% confidence Intervals	Figure
w^{1118}*; fmi-EGFP/+*	8	2454.16	2069 to 2839	1830.24	1569 to 2092	0.27	0.184 to 0.352	12.52	8.568 to 23.25	Fig.2P
dsh^{V26}*/Y; P[acman]-dsh*^{TEV}*/fmi-EGFP* (HS)	8	2280.58	1955 to 2446	1754.36	1620 to 1789	0.24	0.222 to 0.268	47.64	60.62 to 79.33	Fig.2P
dsh^{V26}*/Y; P[acman]-dsh*^{TEV}*/fmi-EGFP* (2 hr HS + 18°C)	8	2096.58	1780 to 2171	1670.26	1541 to 1800	0.22	0.207 to 0.230	86.09	109.9 to 142.9	Fig.2P
dsh^{V26}*/Y; P[acman]-dsh*^{TEV}*/fmi-EGFP; P[CaSpeR]-hs-TEVp/+* (No HS)	8	2399.95	2330 to 2470	1835.06	1727 to 1943	0.25	0.211 to 0.288	48.31	60.62 to 81.97	Fig.2P
dsh^{V26}*/Y; P[acman]-dsh*^{TEV}*/fmi-EGFP; P[CaSpeR]-hs-TEVp/+* (2 hr HS)	8	2096.41	1457 to 2179	1484.57	1305 to 1664	0.30	0.296 to 0.314	31.04	38.32 to 53.85	Fig.2P
dsh^{V26}*/Y; P[acman]-dsh*^{TEV}*/fmi-EGFP; P[CaSpeR]-hs-TEVp/+* (2 hr HS + 18°C)	8	2064.16	1834 to 2294	1336.90	1158 to 1515	0.36	0.342 to 0.387	56.99	48.69 to 68.71	Fig.2P
dsh^{V26} *FRT19A/ ubi-mRFP-nls FRT19A; Ubx-FLP/fmi-EGFP*	6	1644.59	1083 to 1884	949.51	686.9 to 1212	0.43	0.429 to 0.4379	34.78	25.31 to 55.55	Fig.2P

Stbm-EGFP before and after cleavage of Dsh^{TEV}

Genotype	n	Total fluorescent mean intensity	Total mean intensity confidence intervals	Stable amount	Stable amount 95% confidence intervals	y[max]	y[max] with 95% confidence intervals	t[0.5] Half-life	Half-life with 95% confidence Intervals	Figure
w^{1118}*; P[acman]-stbm-EGFP stbm*^{6}*/+*	9	5041.42	4656 to 5427	2878.16	2555 to 3201	0.44	0.339 to 0.539	80.39	60.79 to 118.7	Fig.2R
dsh^{V26}*/Y; P[acman]-dsh*^{TEV}*/P[acman]-stbm-EGFP stbm*^{6} (2 hr HS)	6	4725.57	4134 to 5318	2857.97	1862 to 4061	0.41	0.353 to 0.459	33.98	0.297 to 0.333	Fig.2R
dsh^{V26}*/Y; P[acman]-dsh*^{TEV}*/P[acman]-stbm-EGFP stbm*^{6} (2 hr HS + 18°C)	6	4419.35	3962 to 4876	2711.56	2184 to 3207	0.40	0.367 to 0.429	52.24	41.71 to 69.89	Fig.2R
dsh^{V26}*/Y; P[acman]-dsh*^{TEV}*/P[acman]-stbm-EGFP stbm*^{6}*; P[CaSpeR]-hs-TEVp/+* (No HS)	7	4732.28	3962 to 4876	2722.73	2168 to 3243	0.44	0.362 to 0.503	65.32	53.34 to 84.21	Fig.2R
dsh^{V26}*/Y; P[acman]-dsh*^{TEV}*/P[acman]-stbm-EGFP stbm*^{6}*; P[CaSpeR]-hs-TEVp/+* (2 hr HS)	6	4386.05	4136 to 5329	2655.81	1438 to 3662	0.41	0.362 to 4.450	73.71	62.34 to 90.15	Fig.2R
dsh^{V26}*/Y; P[acman]-dsh*^{TEV}*/P[acman]-stbm-EGFP stbm*^{6}*; P[CaSpeR]-hs-TEVp/+* (2 hr HS + 18°C)	6	4005.26	3708 to 5064	2379.36	1772 to 3058	0.42	0.403 to 0.431	86.84	69.55 to 115.6	Fig.2R
dsh^{V26} *FRT19A/ ubi-mRFP-nls FRT19A; Ubx-FLP/P[acman]-stbm-EGFP stbm*^{6}	6	3241.62	2331 to 4152	1754.66	994 to 2715	0.47	0.424 to 0.514	41.55	34.72 to 51.74	Fig.2R

EGFP-Dgo before and after Dsh^{TEV} cleavage

Genotype	n	Total fluorescent mean intensity	Total mean intensity confidence intervals	Stable amount	Stable amount 95% confidence intervals	y[max]	y[max] with 95% confidence intervals	t[0.5] Half-life	Half-life with 95% confidence Intervals	Figure
w^{1118}*; P[acman-EGFP-dgo dgo*^{380}*/+*	5	1246.17	1084 to 1408	739.64	618.5 to 860.8	0.42	0.364 to 0.477	45.80	39.27 to 54.94	Fig.S2S
dsh^{V26}*/Y; P[acman]-dsh*^{TEV}*/P[acman]-EGFP-dgo dgo*^{380}	5	1193.01	1010 to 1376	625.18	417.3 to 833.1	0.49	0.450 to 0.518	36.13	29.84 to 45.78	Fig.S2S
dsh^{V26}*/Y; P[acman]-dsh*^{TEV}*/P[acman]EGFP-dgo dgo*^{380}*; P[CaSpeR]-hs-TEVp/+* (2 hr HS)	4	1010.81	664.8 to 1357	434.53	238.6 to 630.5	0.58	0.493 to 0.722	46.10	39.64 to 55.07	Fig.S2S

EGFP-Pk in wild-type Dsh and *dsh*^{V26} null tissue

Genotype	n	Total fluorescent mean intensity	Total mean intensity confidence intervals	Stable amount	Stable amount 95% confidence intervals	y[max]	y[max] with 95% confidence intervals	t[0.5] Half-life	Half-life with 95% confidence Intervals	Figure
w^{1118}*; EGFP-pk/+*	5	1166.00	173.5 to 1468	607.30	422.2 to 792.4	0.49	0.537 to 0.586	54.71	47.84 to 63.88	Fig.S2T
dsh^{V26} *FRT19A/ubi-nls-RFP FRT19A; Ubx-FLP/P[acman]EGFP-pk*	6	867.60	733 to 1002	274.26	187.1 to 361.4	0.69	0.662 to 0.727	59.83	52.1 to 70.24	Fig.S2T

Dsh-EGFP after Tom70-HA-vhhGFP acute knockdown with or without blocking Dynamin-dependent endocytosis

Genotype	n	Total fluorescent mean intensity	Total mean intensity confidence intervals	Stable amount	Stable amount 95% confidence intervals	y[max]	y[max] with 95% confidence intervals	t[0.5] Half-life	Half-life with 95% confidence Intervals	Figure
Ubx-FLP, dsh^{V26}*/Y; P[acman]-dsh-EGFP FRT40/P[CaSpeR]-hs-Tom70-HA-vhhGFP FRT40* (No HS)	5	1844.65	1579.6 to 2109.7	1006.81	156.2 to 1257	0.46	0.419 to 0.587	45.77	28.4 to 117.8	Fig.4E
Ubx-FLP, dsh^{V26}*/Y; P[acman]-dsh-EGFP FRT40/P[CaSpeR]-hs-Tom70-HA-vhhGFP FRT40* (2 hr HS)	7	1495.44	1077.4 to 1913.4	590.08	389.7 to 790.4	0.61	0.583 to 633	36.93	26.4 to 27.1	Fig.4E
dsh^{1}*, shi*^{ts1}*, hs-FLP/Y; P[acman]-dsh-EGFP FRT40/hs-Tom70-HA-vhhGFP4 FRT40* (2 hr HS)	5	1977.66	2274.7 to 1680.7	1028.77	777.6 to 1280	0.49	0.407 to 0.532	79.19	62.33 to 108.5	Fig.4E

EGFP-Pk in the same cell as *dsh*^{V26} or in the neighbouring wild-type cell

Genotype	n	Total fluorescent mean intensity	Total mean intensity confidence intervals	Stable amount	Stable amount 95% confidence intervals	y[max]	y[max] with 95% confidence intervals	t[0.5] Half-life	Half-life with 95% confidence Intervals	Figure
EGFP-pk	9	1796.00	1509 to 2083	498.90	331.4 to 666.5	0.71	0.680 to 0.746	45.01	38.48 to 54.2	Fig.S4I
EGFP-pk dsh^{V26}	7	1612.00	1121 to 2103	237.90	79.24 to 396.5	0.83	0.801 to 0.855	42.62	38.01 to 48.49	Fig.S4I
EGFP-pk dsh^{V26} *clone boundary next to pk*$^{pk-sple13}$	8	999.30	799.7 to 1199	353.40	223 to 483.9	0.68	0.649 to 0.705	25.94	21.75 to 32.11	Fig.S4I
EGFP-pk clone boundary next to dsh^{V26} *pk*$^{pk-sple13}$	9	594.10	468.9 to 719.3	151.30	90.34 to 212.3	0.68	0.652 to 0.707	23.96	20.02 to 29.81	Fig.S4I

Table S2. Quantitative FRAP data. Related to Figures 2, S2, 4, S4.

Genotypes for each FRAP experiment, along with the timepoints at which the pupal wings were imaged are shown, along with the number (n) of wings imaged for each genotype, the total fluorescence intensity, the stable amounts, the y[max] (plateau), the half-life and the 95% confidence intervals for each of the data sets. The figure panel relating to the results is also indicated.

Dsh acute knockdown using TEVp, Tom70-HA-vhhGFP or Rpn10-HA-vhhGFP: Compared stable amount, Fig.S1F					
Unpaired t-tests	**Mean Diff.**	**95% CI of diff.**	**Significant?**	**Summary**	**Adjusted p Value**
Dsh^{TEV} no heat shock ***vs.*** *Dsh*^{TEV} 2 hr heat shock + 1 hr recovery	-0.891	-1.011 to -0.788	Yes	***	≤0.0001
Dsh-EGFP with *Tom70-vhhGFP* no heat shock ***vs.*** *Dsh-EGFP* with *Tom70-vhhGFP* 2 hr heat shock	1.265	-0.303 to 2.833	Yes	***	≤0.0001
Dsh-EGFP with *Rpn10-vhhGFP* no heat shock ***vs.*** *Dsh-EGFP* with *Rpn10-vhhGFP* 90 min heat shock	-0.772	-1.111 to -0.432	Yes	**	0.0032
**Fz mean vector polarity: Compared stable amount, Fig.2M**					
ANOVA, Tukey-Kramer's multiple comparison test	**Mean Diff.**	**95% CI of diff.**	**Significant?**	**Summary**	**Adjusted p Value**
dsh^{TEV} *x TEVp* no heat shock ***vs.*** *dsh*^{TEV} *x TEVp* heat shock	0.322	0.193 to 0.451	Yes	***	≤0.0001
dsh^{TEV} *x TEVp* no heat shock ***vs.*** *dsh*^{TEV} *x TEVp* heat shock + 1 hr recovery 18°C	0.439	0.310 to 0.569	Yes	***	≤0.0001
dsh^{TEV} *x TEVp* no heat shock ***vs.*** *dsh*^{TEV} *x TEVp* heat shock + 2 hr recovery 18°C	0.453	0.324 to 0.582	Yes	***	≤0.0001
dsh^{TEV} x TEVp no heat shock ***vs.*** *dsh*^{TEV} *x TEVp* heat shock + 3 hr recovery 18°C	0.471	0.342 to 0.600	Yes	***	≤0.0001
dsh^{TEV} x TEVp no heat shock ***vs.*** *dsh*^{V26}	0.409	0.280 to 0.538	Yes	***	≤0.0001
**Fmi mean vector polarity: Compared stable amount, Fig.2O**					
ANOVA, Tukey-Kramer's multiple comparison test	**Mean Diff.**	**95% CI of diff.**	**Significant?**	**Summary**	**Adjusted p Value**
dsh^{TEV} *x TEVp* no heat shock ***vs.*** *dsh*^{TEV} *x TEVp* heat shock	0.065	0.017 to 0.193	No	na	0.055
dsh^{TEV} *x TEVp* no heat shock ***vs.*** *dsh*^{TEV} *x TEVp* heat shock + 1 hr recovery 18°C	0.275	0.169 to 0.381	Yes	***	≤0.0001
dsh^{TEV} *x TEVp* no heat shock ***vs.*** *dsh*^{TEV} *x TEVp* heat shock + 2 hr recovery 18°C	0.427	0.321 to 0.533	Yes	***	≤0.0001
dsh^{TEV} x TEVp no heat shock ***vs.*** *dsh*^{TEV} *x TEVp* heat shock + 3 hr recovery 18°C	0.431	0.325 to 0.537	Yes	***	≤0.0001
dsh^{TEV} x TEVp no heat shock ***vs.*** *dsh*^{V26}	0.473	0.351 to 0.595	Yes	***	≤0.0001
**Stbm mean vector polarity: Compared stable amount, Fig.2R**					
ANOVA, Tukey-Kramer's multiple comparison test	**Mean Diff.**	**95% CI of diff.**	**Significant?**	**Summary**	**Adjusted p Value**
dsh^{TEV} *x TEVp* no heat shock ***vs.*** *dsh*^{TEV} *x TEVp* heat shock	-0.020	-0.166 to 0.126	No	ns	>0.9999
dsh^{TEV} *x TEVp* no heat shock ***vs.*** *dsh*^{TEV} *x TEVp* heat shock + 1 hr recovery 18°C	0.369	0.224 to 0.515	Yes	***	≤0.0001
dsh^{TEV} *x TEVp* no heat shock ***vs.*** *dsh*^{TEV} *x TEVp* heat shock + 2 hr recovery 18°C	0.343	0.188 to 0.499	Yes	***	≤0.0001
dsh^{TEV} x TEVp no heat shock ***vs.*** *dsh*^{TEV} *x TEVp* heat shock + 3 hr recovery 18°C	0.334	0.179 to 0.489	Yes	***	≤0.0001
dsh^{TEV} x TEVp no heat shock ***vs.*** *dsh*^{V26}	0.444	0.292 to 0.596	Yes	***	≤0.0001
**Fz-EGFP before and after cleavage of DshTEV (FRAP): Compared stable amount, Fig.2N**					
ANOVA, Tukey-Kramer's multiple comparison test	**Mean Diff.**	**95% CI of diff.**	**Significant?**	**Summary**	**Adjusted p Value**
w^{1118}*; fz-EGFP/+* ***vs.*** *dsh*^{V26} *FRT19A/ubi-mRFP-nls FRT19A; Ubx-FLP/+; fz-EGFP/+*	754.2	328.6 to 1180	Yes	***	≤0.0001
w^{1118}*; fz-EGFP/+* ***vs.*** *dsh*^{V26}*/Y; P[acman]-dsh3xTEV/P[CaSpeR]-hs-TEVp; fz-EGFP/+* (no HS)	222.8	-168.2 to 613.9	No	ns	0.613
dsh^{V26}*/Y; P[acman]-dsh3xTEV/+; fz-EGFP/+* (HS) ***vs.*** *dsh*^{V26}*/Y; P[acman]-dsh3xTEV/P[CaSpeR]-hs-TEVp; fz-EGFP/+* (HS)	338.8	-52.28 to 729.8	Yes	*	0.043
dsh^{V26}*/Y; P[acman]-dsh3xTEV/+; fz-EGFP/+* (HS + 1 hr 18°C) ***vs.*** *dsh*^{V26}*/Y; P[acman]-dsh3xTEV/ P[CaSpeR]-hs-TEVp; fz-EGFP/+* (HS + 1 hr 18°C)	610.2	184.6 to 1036	Yes	**	0.001
**Fmi-EGFP before and after cleavage of DshTEV (FRAP): Compared stable amount, Fig.2P**					
ANOVA, Tukey-Kramer's multiple comparison test	**Mean Diff.**	**95% CI of diff.**	**Significant?**	**Summary**	**Adjusted p Value**
w^{1118}*; fmi-EGFP/+* ***vs.*** *dsh*^{V26} *FRT19A/ubi-mRFP-nls FRT19A; Ubx-FLP/fmi-EGFP*	720.7	424.2 to 1017	Yes	***	≤0.0001
w^{1118}*; fmi-EGFP/+* ***vs.*** *dsh*^{V26}*/Y; P[acman]-dsh*^{TEV}*/fmi-EGFP; P[CaSpeR]-hs-TEVp/+* (no HS)	34.1	-249.8 to 318.1	No	ns	0.997
dsh^{V26}*/Y; P[acman]-dsh*^{TEV}*//fmi-EGFP* (HS) ***vs.*** *dshV26/Y; P[acman]-dsh*^{TEV}*//fmi-EGFP; P[CaSpeR]-hs-TEVp/+* (HS)	185.7	-98.26 to 469.6	No	ns	0.347
dsh^{V26}*/Y; P[acman]-dsh*^{TEV}*//fmi-EGFP* (HS + 1 hr 18°C) ***vs.*** *dsh*^{V26}*/Y; P[acman]-dsh*^{TEV}*/fmi-EGFP; P[CaSpeR]-hs-TEVp/+* (HS + 1 hr 18°C)	350.5	66.54 to 634.4	Yes	**	0.009
**Stbm-EGFP before and after cleavage of DshTEV (FRAP): Compared stable amount, Fig.2R**					
ANOVA, Tukey-Kramer's multiple comparison test	**Mean Diff.**	**95% CI of diff.**	**Significant?**	**Summary**	**Adjusted p Value**
w^{1118}*; P[acman]-stbm-EGFP stbm*^{6}*/+* ***vs.*** *dsh*^{V26} *FRT19A/ubi-mRFP-nls FRT19A; Ubx-FLP/P[acman]-stbm-EGFP stbm*^{6}	1024	12.67 to 2034	Yes	*	0.046
w^{1118}*; P[acman]-stbm-EGFP stbm*^{6}*/+* ***vs.*** *dsh*^{V26}*/Y; P[acman]-dsh*^{TEV}*//P[acman]-stbm-EGFP stbm*^{6}*; P[CaSpeR]-hs-TEVp/+* (no HS)	172.5	-897.2 to 1242	No	ns	0.999
dsh^{V26}*/Y; P[acman]-dsh*^{TEV}*//P[acman]-stbm-EGFP stbm*^{6} (HS) ***vs.*** *dsh*^{V26}*/Y; P[acman]-dsh*^{TEV}*/P[acman]-stbm-EGFP stbm*^{6}*; P[CaSpeR]-hs-TEVp/+* (HS)	411.1	-875.5 to 1698	No	ns	0.949
dsh^{V26}*/Y; P[acman]-dsh*^{TEV}*//P[acman]-stbm-EGFP stbm*^{6} (HS + 1 hr 18°C) ***vs.*** *dsh*^{V26}*/Y; P[acman]-dsh*^{TEV}*/ P[acman]-stbm-EGFP stbm*^{6}*; P[CaSpeR]-hs-TEVp/+* (HS + 1hr 18°C)	280.8	-932.2 to 1494	No	ns	0.990
**Stbm-EGFP before and after cleavage of DshTEV (FRAP): Compared stable amount, Fig.S2A-E**					
**Panel A Western of *DshTEV* acute knockdown**	**Mean Diff.**	**95% CI of diff.**	**Significant?**	**Summary**	**Adjusted p Value**
Dunnett's multiple comparisons test					
Dsh No heat shock **vs.** Dsh Control heat shock	-0.098	-2.40 to 2.2	No	ns	0.993
Dsh No heat shock **vs.** Dsh Control heat shock + 1 hr at 18°C	-0.498	-4.346 to 3.35	No	ns	0.817
Dsh No heat shock **vs.** Dsh 2 hr heat shock	0.474	-0.177 to 1.124	No	ns	0.091
Dsh No heat shock **vs.** Dsh 2 hr heat shock + 1 hr at 18°C	0.910	0.668 to 1.152	Yes	**	0.004
**Panel B Western of DshTEV acute knockdown**					
Dunnett's multiple comparisons test					
Fz No heat shock **vs.** Fz Control heat shock	0.11	-0.757 to 0.978	No	ns	0.824
Fz No heat shock **vs.** Fz Control heat shock + 1 hr at 18°C	0.202	-0.624 to 1.027	No	ns	0.496
Fz No heat shock **vs.** Fz 2 hr heat shock	-0.231	-3.334 to 2.872	No	ns	0.956
Fz No heat shock **vs.** Fz 2 hr heat shock + 1 hr at 18°C	0.279	-1.883 to 2.441	No	ns	0.819
**Panel C Western of DshTEV acute knockdown**					

Dunnett's multiple comparisons test					
Fmi No heat shock **vs.** Fmi Control heat shock	-0.382	-6.111 to 5.347	No	ns	0.969
Fmi No heat shock **vs.** Fmi Control heat shock + 1 hr at 18°C	-1.124	-10.98 to 8.73	No	ns	0.862
Fmi No heat shock **vs.** Fmi 2 hr heat shock	-0.332	-1.65 to 0.986	No	ns	0.478
Fmi No heat shock **vs.** Fmi 2 hr heat shock + 1 hr at 18°C	-0.166	-0.461 to 0.128	No	ns	0.143
**Panel D Western of DshTEV acute knockdown**					
Dunnett's multiple comparisons test					
Stbm No heat shock **vs.** Stbm Control heat shock	0.241	-0.160 to 0.641	No	ns	0.129
Stbm No heat shock **vs.** Stbm Control heat shock + 1 hr at 18°C	0.098	-0.613 to 0.810	No	ns	0.791
Stbm No heat shock **vs.** Stbm 2 hr heat shock	-0.1003	-1.786 to 1.586	No	ns	0.979
Stbm No heat shock **vs.** Stbm 2 hr heat shock + 1 hr at 18°C	0.0733	-0.414 to 0.560	No	ns	0.753
**Panel E Western of DshTEV acute knockdown**					
Dunnett's multiple comparisons test					
Pk No heat shock **vs.** Pk Control heat shock	-0.21	-2.774 to 2.354	No	ns	0.942
Pk No heat shock **vs.** Pk Control heat shock + 1 hr at 18°C	-0.003	-1.866 to 1.861	No	ns	0.999
Pk No heat shock **vs.** Pk 2 hr heat shock	0.105	-1.028 to 1.238	No	ns	0.918
Pk No heat shock **vs.** Pk 2 hr heat shock + 1 hr at 18°C	0.329	-0.749 to 1.406	No	ns	0.377
**Fmi (F) and Fz (G) mean vector polarity values after disruption of Dsh-EGFP using vhhGFP: Compared stable amount, Fig.S2F-G**					
**Panel F acute knockdown Fmi mean vector polarity**					
ANOVA, Tukey-Kramer’s multiple comparison test	**Mean Diff.**	**95% CI of diff.**	**Significant?**	**Summary**	**Adjusted P Value**
dsh-EGFP No heat shock ***vs.*** *dsh-EGFP* 2 hr heat shock	0.039	-0.117 to 0.195	No	ns	0.999
dsh-EGFP No heat shock ***vs.*** *dsh-EGFP* 2 hr heat shock + 1h 18°C	0.055	-0.101 to 0.210	No	ns	0.987
dsh-EGFP Tom70vhh No heat shock ***vs.*** *dsh-EGFP Tom70vhh* 2 hr heat shock	0.134	-0.022 to 0.290	No	ns	0.163
dsh-EGFP Tom70vhh No heat shock ***vs.*** *dsh-EGFP Tom70vhh* 2 hr heat shock + 1 hr 18°C	0.278	0.122 to 0.434	Yes	***	≤0.0001
dsh-EGFP Rpn10vhh No heat shock ***vs.*** *dsh-EGFP Rpn10vhh* 2 hr heat shock	-0.1004	-0.251 to 0.050	No	ns	0.524
dsh-EGFP Rpn10vhh No heat shock ***vs.*** *dsh-EGFP Rpn10vhh* 2 hr heat shock + 1 hr 18°C	0.374	0.218 to 0.530	Yes	***	≤0.0001
**Panel G acute knockdown Fz mean vector polarity**					
ANOVA, Tukey-Kramer’s multiple comparison test	**Mean Diff.**	**95% CI of diff.**	**Significant?**	**Summary**	**Adjusted p Value**
dsh-EGFP No heat shock ***vs.*** *dsh-EGFP* 2 hr heat shock	0.006	-0.139 to 0.151	No	ns	>0.9999
dsh-EGFP No heat shock ***vs.*** *dsh-EGFP* 2 hr heat shock + 1 hr 18°C	0.06	-0.085 to 0.206	No	ns	0.955
dsh-EGFP Tom70vhh No heat shock ***vs.*** *dsh-EGFP Tom70vhh* 2 hr heat shock	0.208	0.106 to 0.450	Yes	**	0.0095
dsh-EGFP Tom70vhh No heat shock ***vs.*** *dsh-EGFP Tom70vhh* 2 hr heat shock + 1 hr 18°C	0.333	0.162 to 0.505	Yes	***	≤0.0001
dsh-EGFP Rpn10vhh No heat shock ***vs.*** *dsh-EGFP Rpn10vhh* 2 hr heat shock	0.331	0.159 to 0.503	Yes	***	≤0.0001
dsh-EGFP Rpn10vhh No heat shock ***vs.*** *dsh-EGFP Rpn10vhh* 2 hr heat shock + 1 hr 18°C	0.431	0.259 to 0.603	Yes	***	≤0.0001
**EGFP-Dgo before and after DshTEV cleavage (FRAP): Compared stable amount, Fig.S2S**					
ANOVA, Tukey-Kramer’s multiple comparison test	**Mean Diff.**	**95% CI of diff.**	**Significant?**	**Summary**	**Adjusted P Value**
w^{1118}; P[acman-EGFP-dgo dgo^{380}/+ ***vs.*** *dsh^{V26}/Y; P[acman]-dsh^{TEV}/P[acman-EGFP-dgo] dgo^{380}* (HS)	114.5	-106.4 to 335.3	No	ns	0.380
w^{1118}; P[acman-EGFP-dgo dgo^{380}/+ ***vs.*** *dsh^{V26}/Y; P[acman]-dsh^{TEV}/P[acman-EGFP-dgo] dgo^{380}; P[CaSpeR]-hs-TEVp/+* (HS)	305.1	69.69 to 540.5	Yes	*	0.012
**EGFP-Pk in wild-type Dsh and dsh null tissue (FRAP): Compared stable amount, Fig.S2T**					
Unpaired t-test	**Mean Diff.**	**95% CI of diff.**	**Significant?**	**Summary**	**Adjusted P Value**
w^{1118}; EGFP-pk/+ ***vs.*** *dsh^{V26} FRT19A/ubi-nls-RFP FRT19A; Ubx-FLP/P[acman]EGFP-pk*	607.3	-493.4 to -172.7	Yes	**	0.001
**Distal propergation of Pk: Compared stable amount, Fig.4C**					
ANOVA, Dunnett’s multiple comparison	**Mean Diff.**	**95% CI of diff.**	**Significant?**	**Summary**	**Adjusted P Value**
Cell inside the clone **vs.** 1st cell outside the clone	0.155	-0.130 to 0.441	No	ns	0.606
Cell inside the clone **vs.** 2nd cell outside the clone	0.287	0.001 to 0.573	Yes	*	0.049
Cell inside the clone **vs.** 3rd cell outside the clone	0.487	0.201 to 0.773	Yes	***	0.0002
Cell inside the clone **vs.** 4th cell outside the clone	0.619	0.333 to 0.905	Yes	***	≤0.0001
Cell inside the clone **vs.** 5th cell outside the clone	0.67	0.385 to 0.956	Yes	***	≤0.0001
**Proximal propergation of Pk: Compared stable amount, Fig.4D**					
ANOVA, Dunnett’s multiple comparison	**Mean Diff.**	**95% CI of diff.**	**Significant?**	**Summary**	**Adjusted P Value**
Cell inside the clone edge **vs.** 1st cell outside the clone	0.706	0.469 to 0.944	Yes	***	≤0.0001
Cell inside the clone edge **vs.** 2nd cell outside the clone	0.742	0.504 to 0.979	Yes	***	≤0.0001
Cell inside the clone edge **vs.** 3rd cell outside the clone	0.795	0.558 to 1.032	Yes	***	≤0.0001
**Dsh-EGFP after Tom70-HA-vhhGFP acute knockdown with or without blocking Dynamin-dependent endocytosis: Compared stable amount, Fig.4E**					
ANOVA, Tukey-Kramer’s multiple comparison test	**Mean Diff.**	**95% CI of diff.**	**Significant?**	**Summary**	**Adjusted p Value**
UbxFLP, dsh^{V26}/Y; P[acman]-dsh-EGFP FRT40/P[CaSpeR]-hs-Tom70-HA-vhhGFP FRT40 (No Tom70vhh) ***vs.*** *the cell neighbouring the knockdown clone cells*	416.7	97.29 to 736.2	Yes	*	0.011
UbxFLP, dsh^{V26}/Y; P[acman]-dsh-EGFP FRT40/P[CaSpeR]-hs-Tom70-HA-vhhGFP FRT40 (Cell neighbouring cell knockdown clone) ***vs.*** *the cell neighbouring the knockdown clone cells in a shi^{ts1} background*	-438.7	-758.1 to -119.2	Yes	**	0.008
**Quantitation of Dsh-EGFP and Dsh levels with or without Tom70vhh or Rpn10vhh: Compared stable amount, Fig.S4A**					
Unpaired t-test	**Mean Diff.**	**95% CI of diff.**	**Significant?**	**Summary**	**Adjusted P Value**
Dsh-EGFP Tom70vhh No heat shock ***vs.*** *Dsh-EGFP Tom70vhh* 2 hr heat shock	1.579	-0.288 to 3.447	No	ns	0.079
Dsh Tom70vhh No heat shock ***vs.*** *Dsh Tom70vhh* 2 hr heat shock	1.381	-0.915 to 3.677	No	ns	0.17
Dsh-EGFP Rpn10vhh No heat shock ***vs.*** *Dsh-EGFP Rpn10vhh* 90 min heat shock	-0.444	-0.781 to -0.108	Yes	*	0.022
Dsh Rpn10vhh No heat shock ***vs.*** *Dsh Rpn10vhh* 90 min heat shock	0.034	-0.649 to 0.718	No	ns	0.896
**Dsh-EGFP clones in Tom70-vhh knockdown tissue: Compared stable amount, Fig.S4B**					

Paired t-test	Mean Diff.	95% CI of diff.	Significant?	Summary	Adjusted P Value
Proximodistal membrane localised Pk in cells outside of the clone **vs.** *At the proximal edge of cells at the proximal edge of Dsh-EGFP clones*	4.925	-1.4 to 11.25	No	ns	0.108
***EGFP-Pk* in the same cell as d*sh*^{V26} or in the neighbouring wild-type cell: Compared stable amount, Fig.S4I**					
ANOVA, Sidak's multiple comparisons test	**Mean Diff.**	**95% CI of diff.**	**Significant?**	**Summary**	**Adjusted P Value**
(2nd column) *EGFP-pk* in a wild-type cell next to *pk*$^{pk-sple13}$ *dsh*^{V26} cells n=9 **vs.** (4th column) *EGFP-pk* in a wild-type wing n=9	-347.6	-536.4 to -158.8	Yes	***	0.0002
(2nd column) *EGFP-pk* in a wild-type cell next to *pk*$^{pk-sple13}$ *dsh*^{V26} cells n=9 **vs.** (1st column) *EGFP-pk in a dsh*^{V26} mutant cell next to *pk*$^{pk-sple13}$ *dsh* wild-type cells n=8	-202.1	-397.1 to -7.171	Yes	*	0.04
(1st column) *EGFP-pk* in a *dsh*^{V26} mutant cell next to *pk*$^{pk-sple13}$ mutant *dsh* wild-type cells n=8 **vs.** (4th column) EGFP-pk in a wild-type wing n=9	115.5	-97.15 to 328.2	No	ns	0.209
**Pk membrane intensity on and near *dsh*^{V26} clones: Compared stable amount, Fig.S4J**					
ANOVA, Tukey-Kramer's multiple comparison test	**Mean Diff.**	**95% CI of diff.**	**Significant?**	**Summary**	**Adjusted P Value**
outside *dsh*^{V26} tissue ***vs.*** Distal edge of the clone	9.175	0.700 to 17.65	Yes	*	0.034
outside *dsh*^{V26} tissue **vs.** One distal cell away form the clone	7.09	-0.137 to 14.32	No	ns	0.055
outside *dsh*^{V26} tissue **vs.** Two distal cell away form the clone	5.101	-0.371 to 10.57	No	ns	0.068
outside *dsh*^{V26} tissue **vs.** Three distal cell away form the clone	2.07	-3.25 to 7.385	No	ns	0.664

Table S3. Detailed statistical comparisons. Related to Figures 2, 4, S2, S4.

Detailed results for the statistical tests carried out on the data shown in the figures are shown. The statistical test (either ANOVA or t-test is indicated), as well as the names of the post hoc tests. These include the Dunnett's multiple comparisons test (for comparing the control to the other experimental conditions), and the Šídák's multiple comparisons test (to compare pre-selected pairs of conditions within an experiment). The table also shows the mean difference and 95% confidence intervals. Stars indicate statistical significance *$P \leq 0.05$ **$P \leq 0.01$ and ***$P \leq 0.001$. The figure panel relating to each statistical test is also indicated.
